# Hamiltonian simulation algorithms for near-term quantum hardware

**DOI:** 10.1038/s41467-021-25196-0

**Published:** 2021-08-17

**Authors:** Laura Clinton, Johannes Bausch, Toby Cubitt

**Affiliations:** 1 PhaseCraft Ltd., London, UK; 2 Department of Computer Science, University College London, London, UK; 3 CQIF, DAMTP, University of Cambridge, Cambridge, UK

**Keywords:** Quantum information, Quantum simulation

## Abstract

The quantum circuit model is the de-facto way of designing quantum algorithms. Yet any level of abstraction away from the underlying hardware incurs overhead. In this work, we develop quantum algorithms for Hamiltonian simulation "one level below” the circuit model, exploiting the underlying control over qubit interactions available in most quantum hardware and deriving analytic circuit identities for synthesising multi-qubit evolutions from two-qubit interactions. We then analyse the impact of these techniques under the standard error model where errors occur per gate, and an error model with a constant error rate per unit time. To quantify the benefits of this approach, we apply it to time-dynamics simulation of the 2D spin Fermi-Hubbard model. Combined with new error bounds for Trotter product formulas tailored to the non-asymptotic regime and an analysis of error propagation, we find that e.g. for a 5 × 5 Fermi-Hubbard lattice we reduce the circuit depth from 1, 243, 586 using the best previous fermion encoding and error bounds in the literature, to 3, 209 in the per-gate error model, or the circuit-depth-equivalent to 259 in the per-time error model. This brings Hamiltonian simulation, previously beyond reach of current hardware for non-trivial examples, significantly closer to being feasible in the NISQ era.

## Abstract (web summary)

The way quantum simulation algorithms are translated into specific hardware implementations often translates into additional overhead. Here, the authors improve the efficiency of Hamiltonian simulation using a method that allows efficient synthesis of multi-qubit evolutions from two-qubit interactions.

## Introduction

Quantum computing is on the cusp of entering the era in which quantum hardware can no longer be simulated effectively classically, even on the world’s biggest supercomputers^1–5^. Google recently achieved the first so-called "quantum supremacy” milestone demonstrating this^6^. While reaching this milestone is an impressive experimental physics achievement, the very definition of this goal allows it to be a demonstration that has no useful practical applications^7^. The recent Google results are of exactly this nature. By far the most important question for quantum computing now is to determine whether there are useful applications of this class of noisy, intermediate-scale quantum (NISQ) hardware^8^.

However, current quantum hardware is still extremely limited, with ≈50 qubits capable of implementing quantum circuits up to a gate depth of ≈20^6^. This is far too limited to run useful instances of even the simplest textbook quantum algorithms, let alone implement the error correction and fault tolerance required for large-scale quantum computations. Estimates of the number of qubits and gates required to run Shor’s algorithm on integers that cannot readily be factored on classical computers place it—and related number-theoretic algorithms—well into the regime of requiring a fully scalable, fault-tolerant quantum computer^9,10^. Studies of practically relevant combinatorial problems tell a similar story for capitalising on the quadratic speedup of Grover’s algorithm^11^. Quantum computers are naturally well suited for simulation of quantum many-body systems^12,13^—a task that is notoriously difficult on classical computers. Quantum simulation is likely to be one of the first practical applications of quantum computing. But, while the number of qubits required to run interesting quantum simulations may be lower than for other applications, careful studies of the gate counts required for a quantum chemistry simulation of molecules that are not easily tractible classically^14^, or for simple condensed matter models^15^, remain far beyond current hardware.

With severely resource-constrained hardware such as this, squeezing every ounce of performance out of it is crucial. The quantum circuit model is the standard way to design quantum algorithms, and quantum gates and circuits provide a highly convenient abstraction of quantum hardware. Circuits sit at a significantly lower level of abstraction than even assembly code in classical computing. But any layer of abstraction sacrifices some overhead for the sake of convenience. The quantum circuit model is no exception.

In the underlying hardware, quantum gates are typically implemented by controlling interactions between qubits. E.g. by changing voltages to bring superconducting qubits in and out of resonance, or by laser pulses to manipulate the internal states of trapped ions. By restricting to a fixed set of standard gates, the circuit model abstracts away the full capabilities of the underlying hardware. In the NISQ era, it is not clear this sacrifice is justified. The Solovay-Kitaev theorem tells us that the overhead of any particular choice of universal gate set is at most poly-logarithmic^16,17^. But when the available circuit depth is limited to ≈20, even a constant factor improvement could make the difference between being able to run an algorithm on current hardware, and being beyond the reach of foreseeable hardware.

The advantages of designing quantum algorithms "one level below” the circuit model become particularly acute in the case of Hamiltonian time-dynamics simulation. To simulate evolution under a many-body Hamiltonian *H* = ∑_〈*i*, *j*〉_*h*_*ij*_, the basic Trotterization algorithm^13,18^ repeatedly time-evolves the system under each individual interaction *h*_*ij*_ for a small time-step *δ*,

1 $${{{{{{\rm{e}}}}}}}^{-{{{{{\rm{i}}}}}}HT}\simeq \mathop{\prod }\limits_{n=0}^{T/\delta }\left(\mathop{\prod}\limits_{\langle i,j\rangle }{{{{{{\rm{e}}}}}}}^{-{{{{{\rm{i}}}}}}{h}_{ij}\delta }\right).$$

To achieve good precision, *δ* must be small. In the circuit model, each ${{{{{{\rm{e}}}}}}}^{-{{{{{\rm{i}}}}}}{h}_{ij}\delta }$ Trotter step necessarily requires at least one quantum gate to implement. Thus the required circuit depth—and hence the total run-time—is at least *T*/*δ*. Contrast this with the run-time if we were able to implement ${{{{{{\rm{e}}}}}}}^{-{{{{{\rm{i}}}}}}{h}_{ij}\delta }$ directly in time *δ*. The total run-time would then be *T*, which improves on the circuit-model algorithm by a factor of 1/*δ*. This is "only” a constant factor improvement, in line with the Solovay-Kitaev theorem. But this "constant” can be very large; indeed, it diverges to *∞* as the precision of the algorithm increases.

It is unrealistic to assume the hardware can implement ${{{{{{\rm{e}}}}}}}^{-{{{{{\rm{i}}}}}}{h}_{ij}\delta }$ for any desired interaction *h*_*ij*_ and any time *δ*. Furthermore, the available interactions are typically limited to at most a handful of specific types, determined by the underlying physics of the device’s qubit and quantum gate implementations. And these interactions cannot be switched on and off arbitrarily fast, placing a limit on the smallest achievable value of *δ*. There are also experimental challenges associated with implementing gates with small *δ* with the same fidelities as those with *δ* ≈ O(1).

A major criticism of analogue computation (classical and quantum) is that it cannot cope with errors and noise. The "N” in NISQ stands for "noisy”; errors and noise will be a significant factor in all foreseeable quantum hardware. But near-term hardware has few resources to spare even on basic error correction, let alone fault tolerance. Indeed, near-term hardware may not always have the necessary capabilities. E.g. the intermediate measurements required for active error correction are not possible in all superconducting circuit hardware [ref. ^19^, Sec. II].

Algorithms that cope well with errors and noise, and still give reasonable results without active error correction or fault tolerance, are thus critical for NISQ applications.

Designing algorithms "one level below” the circuit model can also in some cases reduce the impact of errors and noise during the algorithm. Again, this benefit is particularly acute in Hamiltonian simulation algorithms. If an error occurs on a qubit in a quantum circuit, a two-qubit gate acting on the faulty qubit can spread the error to a second qubit. In the absence of any error correction or fault tolerance, errors can spread to an additional qubit with each two-qubit gate applied, so that after circuit depth *n* the error can spread to all *n* qubits.

In the circuit model, each ${{{{{{\rm{e}}}}}}}^{-{{{{{\rm{i}}}}}}{h}_{ij}\delta }$ Trotter step requires at least one two-qubit gate. So a single error can be spread throughout the quantum computer after simulating time-evolution for time as short as *δn*. However, if a two-qubit interaction ${{{{{{\rm{e}}}}}}}^{-{{{{{\rm{i}}}}}}{h}_{ij}\delta }$ is implemented directly, one would intuitively expect it to only "spread the error” by a small amount *δ* for each such time-step. Thus we might expect it to take time *O*(*n*) before the error can propagate to all *n* qubits—a factor of 1/*δ* improvement. Another way of viewing this is that, in the circuit model, the Lieb-Robinson velocity^20^ at which effects propagate in the system is always *O*(1), regardless of what unitary dynamics is being implemented by the overall circuit. In contrast, the Trotterized Hamiltonian evolution has the same Lieb-Robinson velocity as the dynamics being simulated: *O*(1/*δ*) in the same units.

The Fermi-Hubbard model is believed to capture, in a simplified toy model, key aspects of high-temperature superconductors, which are still less well understood theoretically than their low-temperature brethren. Its Hamiltonian is given by a combination of on-site and hopping terms:

2 $${H}_{{{{{{\rm{FH}}}}}}}	:=\mathop{\sum }\limits_{i=1}^{N}{h}_{{{{{{\rm{on-site}}}}}}}^{(i)}\,+\mathop{\sum}\limits_{i < j,\sigma }{h}_{{{{{{\rm{hopping}}}}}}}^{(i,j,\sigma )}\\ 	:=u\mathop{\sum }\limits_{i=1}^{N}{a}_{i\uparrow }^{{{\dagger}} }{a}_{i\uparrow }{a}_{i\downarrow }^{{{\dagger}} }{a}_{i\downarrow }+v\mathop{\sum}\limits_{i < j,\sigma }\left({a}_{i\sigma }^{{{\dagger}} }{a}_{j\sigma }+{a}_{j\sigma }^{{{\dagger}} }{a}_{i\sigma }\right).$$

describing electrons with spin *σ* = *↑* or *↓* hopping between neighbouring sites on a lattice, with an on-site interaction between opposite-spin electrons at the same site. The Fermi-Hubbard model serves as a particularly good test-bed for NISQ Hamiltonian simulation algorithms for a number of reasons [ref. ^21^, Sec. IV], beyond the fact that it is a scientifically interesting model in its own right:

1. The Fermi-Hubbard model was a famous, well-studied condensed matter model long before quantum computing was proposed. It is therefore less open to the criticism of being an artificial problem tailored to fit the algorithm.
2. It is a fermionic model, which poses particular challenges for simulation on (qubit-based) quantum computers. Most of the proposed practical applications of quantum simulation involve fermionic systems, either in quantum chemistry or materials science. So achieving quantum simulation of fermionic models is an important step on the path to practical quantum computing applications.
3. There have been over three decades of research developing ever-more-sophisticated classical simulations of Fermi-Hubbard-model physics^22^. This gives clear benchmarks against which to compare quantum algorithms. And it reduces the likelihood of there being efficient classical algorithms, which have not been discovered because little interest or effort has been devoted to the model.

The state-of-the-art quantum circuit-model algorithm for simulating the time dynamics of the 2D Fermi-Hubbard model on an 8 × 8 lattice requires ≈10^7^ Toffoli gates [ref. ^15^, Sec. C: Tb. 2]. This includes the overhead for fault tolerance, which is necessary for the algorithm to achieve reasonable precision with the gate fidelities available in current and near-term hardware. But it additionally incorporates performing phase estimation, which is a significant extra contribution to the gate count. Thus, although this result is indicative of the scale required for standard circuit-model Hamiltonian simulation, a direct comparison of this result with time-dynamics simulation would be unfair.

To establish a fair benchmark, using available Trotter error bounds from the literature^23^ with the best previous choice of fermion encoding in the literature^24^, we calculate that one could achieve a Fermi-Hubbard time-dynamics simulation on a 5 × 5 square lattice, up to time *T* = 7 and to within 10% accuracy, using 50 qubits and 1,243, 586 standard two-qubit gates. This estimate assumes the effects of decoherence and errors in the circuit can be neglected, which is certainly over-optimistic.

Our results rely on developing more sophisticated techniques for synthesising many-body interactions out of the underlying one- and two-qubit interactions available in the quantum hardware (see Results). This gives us access to ${{{{{{\rm{e}}}}}}}^{-{{{{{\rm{i}}}}}}{h}_{ij}\delta }$ for more general interactions *h*_*ij*_. We then quantify the type of gains discussed here under two precisely defined error models, which correspond to different assumptions about the hardware. By using the aforementioned techniques to synthesise local Trotter steps, exploiting a recent fermion encoding specifically designed for this type of algorithm^25^, deriving tighter error bounds on the higher-order Trotter expansions that account for all constant factors, and carefully analysing analytically and numerically the impact and rate of spread of errors in the resulting algorithm, we improve on this by multiple orders of magnitude even in the presence of decoherence. For example, we show that a 5 × 5 Fermi-Hubbard time-dynamics simulation up to time *T* = 7 can be performed to 10% accuracy in what we refer to as a per-gate error model with ≈50 qubits and the equivalent of circuit depth 72,308. This is a conservative estimate and based on analytic Trotter error bounds that we derive in this paper. Using numerical extrapolation of Trotter errors, a circuit depth of 3209 can be reached. In the second error model, which we refer to as a per-time error model, we prove rigorously that the same simulation is achievable in a circuit-depth-equivalent run-time of 1686; numerical error computations bring this down to 259. In the per-time model, for some parameter regimes we are also able to exploit the inherent partial error-detection properties of local fermionic encodings to enable error mitigation strategies to reduce the resource cost. This brings Hamiltonian simulation, previously beyond reach of current hardware for non-trivial examples, significantly closer to being feasible in the NISQ era.

## Results and discussion

### Circuit error models

We consider two error models for quantum computation in this work. The first error model assumes that noise occurs at a constant rate per gate, independent of the time it takes to implement that gate. This is the standard error model in quantum computation theory, in which the cost of a computation is proportional to its circuit depth. We refer to this model as the per-gate error model. The second error model assumes that noise occurs at a constant rate per unit time. This is the traditional model of errors in physics, where dissipative noise is more commonly modelled by continuous-time master equations, which translates to the per-time error model. In this model, the errors accumulate proportionately to the time the interactions in the system are switched on, thus with the total pulse lengths. We refer to this as the per-time error model We emphasise that these error models are not fundamentally about execution time, but about an error budget required to execute a particular circuit. While it is clear that deeper circuits experience more decoherence, how much each gate contributes to it can be analysed from two different perspectives. The two error models we study correspond to two difference models of how noise scales in quantum hardware.

Which of these more accurately models errors in practice is hardware dependent. For example, in NMR experiments, the per-time model is common^26–28^. The per-time model is not without basis in more recent quantum hardware, too. Recent work has developed and experimentally tested duration-scaled two-qubit gates using Qiskit Pulse and IBM devices^29,30^. In ref. ^30^ the authors experimentally observe braiding of Majorana zero modes using and IBM device and parameterised two-qubit gates. They also find a relationship between relative gate errors and the duration of these parameterised gates, which is further validated in ref. ^29^. The authors of ref. ^29^ explicitly attribute the reduction in error—seen using these duration-scaled gates in place of CNOT gates—to the shorter schedules of the scaled gates relative to the coherence time.

Nonetheless, the standard per-gate error model is also very relevant to current quantum hardware hardware. Therefore, throughout this paper we carry out full error analyses of all our algorithms in both of these error models.

Both of these error models are idealisations. Both are reasonable from a theoretical perspective and supported by certain experiments. Analysing both error models allows different algorithm implementations to be compared fairly under different error regimes. In particular, analysing both of these error models gives a more stringent test of new techniques than considering only the "standard error model” of quantum computation, which corresponds to the per-gate model.

We show that in both error models, significant gains can be achieved using our new techniques.

In our analysis, for simplicity we treat single-qubit gates as a free resource in both error models. There are three reasons for making this simplification, First, single-qubit gates can typically be implemented with an order-of-magnitude higher fidelity in hardware, so contribute significantly less to the error budget than two-qubit gates. Second, they do not propagate errors to the same extent as two-qubit gates (cf. only costing T gates in studies of fault-tolerant quantum computation). Third, any quantum circuit can be decomposed with at most a single layer of single-qubit gates between each layer of two-qubit gates. Thus including single-qubit gates in the per-gate error model changes the absolute numbers by a constant factor ≤2 in the worst case. Nor does it significantly affect comparisons between different algorithm designs. This is particularly true of product-formula simulation algorithms, where the algorithms are composed of the same layers of gates repeated over and over.

Additionally, there is a benefit to utilising our synthesis techniques regardless of error model. Decomposing the simulation into gates of the form ${{{{{{\rm{e}}}}}}}^{-{{{{{\rm{i}}}}}}{h}_{ij}\delta }$ using these methods allows us to exploit the underlying error-detection properties of fermionic encodings, as explained in Supplementary Methods and demonstrated in Fig. 2 (see below).

Tables 1 and 2 compare these results, showing how the combination of sub-circuit algorithms, recent advances in fermion encodings (VC = Verstraete-Cirac encoding^24^, compact = encoding reported in ref. ^25^), and tighter Trotter bounds (both analytic and numeric) successively reduce the run-time of the simulation algorithm (${{{{{{\mathcal{T}}}}}}}_{{{{{{\rm{cost}}}}}}}=$ circuit depth for per-gate error model, or sum of pulse lengths for per-time error model).

**Table 1:** Per-gate run-times.

Fermion encoding	Trotter bounds	Standard decomposition	Sub-circuit decomposition
VC	Ref. ^23^, Prop. F.4.	1,243,586	977,103
	Analytic	121,478	95,447
	Numeric	5391	4236
Compact	Analytic	98,339	72,308
	Numeric	4364	3209

A comparison of the run-time ${{{{{{\mathcal{T}}}}}}}_{{{{{{\rm{cost}}}}}}}$ for lattice size *L* × *L* with *L* = 5, overall simulation time *T* = 7 and target Trotter error *ϵ*_target_ = 0.1, with Λ = 5 fermions and coupling strengths ∣*u*∣, ∣*v*∣ ≤ *r* = 1. Obtained by minimising over product formulas up to 4th order. ${{{{{{\mathcal{T}}}}}}}_{{{{{{\rm{cost}}}}}}}=$ circuit depth for per-gate error model. In either gate decomposition case—standard and sub-circuit—we account single-qubit rotations as a free resource as explained in the Introduction; the value of ${{{{{{\mathcal{T}}}}}}}_{{{{{{\rm{cost}}}}}}}$ depends only on the two-qubit gates/interactions. Two-qubit unitaries are counted by unit time per gate in the per-gate error model. Here compact and VC denote the choice of fermionic encoding.

**Table 2:** Per-time run-times.

Fermion encoding	Trotter bounds	Standard decomposition	Sub-circuit decomposition
VC	Ref. ^23^, Prop. F.4.	976,710	59,830
	Analytic	95,409	17,100
	Numeric	4234	1669
compact	Analytic	77,236	1686
	Numeric	3428	259

A comparison of the run-time ${{{{{{\mathcal{T}}}}}}}_{{{{{{\rm{cost}}}}}}}$ for lattice size *L* × *L* with *L* = 5, overall simulation time *T* = 7 and target Trotter error *ϵ*_target_ = 0.1, with Λ = 5 fermions and coupling strengths ∣*u*∣, ∣*v*∣ ≤ *r* = 1. Obtained by minimising over product formulas up to 4th order. ${{{{{{\mathcal{T}}}}}}}_{{{{{{\rm{cost}}}}}}}={{{{{{\mathcal{T}}}}}}}_{{{{{{\rm{cost}}}}}}}({{{{{{\mathcal{P}}}}}}}_{p}{({\delta }_{0})}^{T/{\delta }_{0}})$ for per-time error model. In either gate decomposition case—standard and sub-circuit—we account single-qubit rotations as a free resource; the value of ${{{{{{\mathcal{T}}}}}}}_{{{{{{\rm{cost}}}}}}}$ depends only on the two-qubit gates/interactions. Two-qubit unitaries are counted by their respective pulse lengths. Here compact and VC denote the choice of fermionic encoding.

### Synthesis of encoded Fermi-Hubbard Hamiltonian Trotter layers

To simulate fermionic systems on a quantum computer, one must encode the fermionic Fock space into qubits. There are many encodings in the literature^31^ but we confine our analysis to two: the Verstraete-Cirac (VC) encoding^24^, and the compact encoding recently introduced in ref. ^25^. We have selected these two encodings as they minimise the maximum Pauli weight of the encoded interactions, which is a key factor in the efficiency of Trotter-based algorithms and of our sub-circuit techniques: weight-4 (VC) and weight-3 (compact), respectively. By comparison, the classic Jordan-Wigner transformation^31^ results in a maximum Pauli weight that scales as as ${{{{{\rm{O}}}}}}\left(L\right)$ with the lattice size *L*; the Bravyi-Kitaev encoding^32^ has interaction terms of weight $O({{{{\mathrm{log}}}}}\,L)$; and the Bravyi-Kitaev superfast encoding^32^ results in weight-8 interactions.

Under the compact encoding, the fermionic operators in Eq. (2) are mapped to operators on qubits arranged on two stacked square grids of qubits (one corresponding to the spin up, and one to the spin down sector, as shown in Supplementary Fig. 1d), augmented by a face-centred ancilla in a checkerboard pattern, with an enumeration explained in Supplementary Fig. 1a. The on-site, horizontal and vertical local terms in the Fermi-Hubbard Hamiltonian Eq. (2) are mapped under this encoding to qubit operators as follows:

3 $${h}_{{{{{{\rm{on-site}}}}}}}^{(i)}\to \frac{u}{4}\left({\mathbb{1}}-{Z}_{i\uparrow }\right)\left({\mathbb{1}}-{Z}_{i\downarrow }\right)$$

4 $${h}_{{{{{{\rm{hopping,hor}}}}}}}^{(i,j,\sigma )}\to \frac{v}{2}\left({X}_{i,\sigma }{X}_{j,\sigma }{Y}_{{f}_{ij}^{\prime},\sigma }+{Y}_{i,\sigma }{Y}_{j,\sigma }{Y}_{{f}_{ij}^{\prime},\sigma }\right)$$

5 $${h}_{{{{{{\rm{hopping,vert}}}}}}}^{(i,j,\sigma )}\to \frac{v}{2}{(-1)}^{g(i,j)}\left({X}_{i,\sigma }{X}_{j,\sigma }{X}_{{f}_{ij}^{\prime},\sigma }+{Y}_{i,\sigma }{Y}_{j,\sigma }{X}_{{f}_{ij}^{\prime},\sigma }\right),$$

where qubit ${f}_{ij}^{\prime}$ is the face-centered ancilla closest to vertex (*i*, *j*), and *g*(*i*, *j*) indicates an associated sign choice in the encoding, as explained in ref. ^25^.

If the VC encoding is used, the fermionic operators in Eq. (2) are mapped to qubits arranged on two stacked square grids of qubits (again with one corresponding to spin up, the other to spin down, as shown in Supplementary Fig. 2, augmented by an ancilla qubit for each data qubit and with an enumeration explained in Supplementary Fig. 2a. In this case the on-site, horizontal and vertical local terms are mapped to

6 $${h}_{{{{{{\rm{on-site}}}}}}}^{(i)}\to \frac{u}{4}\left({\mathbb{1}}-{Z}_{i\uparrow }\right)\left({\mathbb{1}}-{Z}_{i\downarrow }\right)$$

7 $${h}_{{{{{{\rm{hopping,hor}}}}}}}^{(i,j,\sigma )}\to \frac{v}{2}\left({X}_{i,\sigma }{Z}_{i^{\prime} ,\sigma }{X}_{j,\sigma }+{Y}_{i,\sigma }{Z}_{i^{\prime} ,\sigma }{Y}_{j,\sigma }\right)$$

8 $${h}_{{{{{{\rm{hopping,vert}}}}}}}^{(i,j,\sigma )}\to \frac{v}{2}\left({X}_{i,\sigma }{Y}_{i^{\prime} ,\sigma }{Y}_{j,\sigma }{X}_{j^{\prime} ,\sigma }-{Y}_{i,\sigma }{Y}_{i^{\prime} ,\sigma }{X}_{j,\sigma }{X}_{j^{\prime} ,\sigma }\right),$$

where $i^{\prime}$ indicates the ancilla qubit associated with qubit *i*.

In both encodings, we partition the resulting Hamiltonian *H*—a sum of on-site, horizontal and vertical qubit interaction terms on the augmented square lattice—into *M* = 5 layers *H* = *H*_1_ + *H*_2_ + *H*_3_ + *H*_4_ + *H*_5_, as shown in Supplementary Figs. 1 and 2. The Hamiltonians for each layer do not commute with one another. Each layer is a sum of mutually-commuting local terms acting on disjoint subsets of the qubits. For instance, ${H}_{5}={\sum }_{i}{h}_{{{{{{\rm{on-site}}}}}}}^{(i)}$ is a sum of all the two-local, non-overlapping, on-site terms.

The Trotter product formula ${{{{{{\mathcal{P}}}}}}}_{p}(T,\delta )$ comprises local unitaries, corresponding to the local interaction terms that make up the five layers of Hamiltonians that we decomposed the Fermi-Hubbard Hamiltonian into.

In order to implement each step of the product formula as a sequence of gates, we would ideally simply execute all two-, three- (for the compact encoding) or four-local (for the VC encoding) interactions necessary for the time evolution directly within the quantum computer. Yet this is an unrealistic assumption, as the quantum device is more likely to feature a very restricted set of one- and two-qubit interactions.

As outlined in the introduction, we assume in our model that arbitrary single-qubit unitaries are available, and that we have access to the continuous family of gates $\{\exp ({{{{{\rm{i}}}}}}tZ\otimes Z)\}$ for arbitrary values of *t*. In contrast, the gates we wish to implement all have the form $\exp ({{{{{\rm{i}}}}}}\delta {Z}^{\otimes k})$ for *k* = 3 or 4. (Or different products of *k* Pauli operators, but these are all equivalent up to local unitaries, which we are assuming are available.)

It is well known that a unitary describing the evolution under any *k*-local Pauli interaction can be straightforwardly decomposed into CNOT gates and single-qubit rotations [ref. ^18^,Sec. 4.7.3]. For instance, we can decompose evolution under a 3-local Pauli as

9 $${{{{{{\rm{e}}}}}}}^{{{{{{\rm{i}}}}}}\delta {Z}_{1}{Z}_{2}{Z}_{3}}={{{{{{\rm{e}}}}}}}^{-{{{{{\rm{i}}}}}}\pi /4{Z}_{1}{X}_{2}}{{{{{{\rm{e}}}}}}}^{{{{{{\rm{i}}}}}}\delta {Y}_{2}{Z}_{3}}{{{{{{\rm{e}}}}}}}^{{{{{{\rm{i}}}}}}\pi /4{Z}_{1}{X}_{2}},$$

where we then further decompose the remaining 2-local evolutions in Eq. (9) using the exact same method as

10 $${{{{{{\rm{e}}}}}}}^{{{{{{\rm{i}}}}}}\delta {Y}_{2}{Z}_{3}}={{{{{{\rm{e}}}}}}}^{-{{{{{\rm{i}}}}}}\pi /4{Y}_{2}{X}_{3}}{{{{{{\rm{e}}}}}}}^{{{{{{\rm{i}}}}}}\delta {Y}_{3}}{{{{{{\rm{e}}}}}}}^{{{{{{\rm{i}}}}}}\pi /4{Y}_{2}{X}_{3}}.$$

This effectively corresponds to decomposing ${{{{{{\rm{e}}}}}}}^{{{{{{\rm{i}}}}}}\delta {Z}_{1}{Z}_{2}{Z}_{3}}$ into CNOT gates and single-qubit rotations, as ${{{{{{\rm{e}}}}}}}^{\pm {{{{{\rm{i}}}}}}\pi /4{Z}_{i}{Z}_{j}}$ is equivalent to a CNOT gate up to single-qubit rotations. To generate evolution under any *k*-local Pauli interaction we can simply iterate this procedure, which yields a constant overhead ∝ 2(*k* − 1) × *π*/4.

Can we do better? Even optimised variants of Solovay-Kitaev to decompose multi-qubit gates—beyond introducing an additional error—generally yield gate sequences multiple orders of magnitude larger, as e.g. demonstrated in ref. ^33^. While more recent results conjecture that an arbitrary three-qubit gate can be implemented with at most eight O(1) two-local entangling gates^34^, this is still worse than the conjugation method for the particular case of a rank one Pauli interaction that we are concerned with.

For small pulse times *δ*, the existing decompositions are thus inadequate, as they all introduce a gate cost Ω(1) + O(*δ*). In this paper, we develop a series of analytic pulse sequence identities (see Supplementary Lemmas 7 and 8 in Supplementary Methods, which allow us to decompose the three-qubit and four-qubit gates as approximately The approximations in Eqs. (11) and (12) are shown to first order in *δ*. Exact analytic expressions, which also hold for *δ* ≥ 1, are derived in Supplementary Methods. The constants in Eq. (12) have been rounded to the third significant figure.

11 $${{{{{{\rm{e}}}}}}}^{{{{{{\rm{i}}}}}}\delta {Z}_{1}{Z}_{2}{Z}_{3}}\approx\,	 {{{{{{\rm{e}}}}}}}^{-{{{{{\rm{i}}}}}}\sqrt{\delta /2}{Z}_{1}{X}_{2}}{{{{{{\rm{e}}}}}}}^{{{{{{\rm{i}}}}}}\sqrt{\delta /2}{Y}_{2}{Z}_{3}}\\ 	\times {{{{{{\rm{e}}}}}}}^{{{{{{\rm{i}}}}}}\sqrt{\delta /2}{Z}_{1}{X}_{2}}{{{{{{\rm{e}}}}}}}^{-{{{{{\rm{i}}}}}}\sqrt{\delta /2}{Y}_{2}{Z}_{3}},$$

12 $${{{{{{\rm{e}}}}}}}^{{{{{{\rm{i}}}}}}\delta {Z}_{1}{Z}_{2}{Z}_{3}{Z}_{4}}\approx\,	 {{{{{{\rm{e}}}}}}}^{-{{{{{\rm{i}}}}}}0.22{\delta }^{2/3}{Y}_{2}{Z}_{3}{Z}_{4}}{{{{{{\rm{e}}}}}}}^{-{{{{{\rm{i}}}}}}1.13{\delta }^{1/3}{Z}_{1}{X}_{2}}{{{{{{\rm{e}}}}}}}^{{{{{{\rm{i}}}}}}0.44{\delta }^{2/3}{Y}_{2}{Z}_{3}{Z}_{4}}\\ 	\times {{{{{{\rm{e}}}}}}}^{{{{{{\rm{i}}}}}}1.13{\delta }^{1/3}{Z}_{1}{X}_{2}}{{{{{{\rm{e}}}}}}}^{-{{{{{\rm{i}}}}}}0.22{\delta }^{2/3}{Y}_{2}{Z}_{3}{Z}_{4}}.$$

In reality we use the exact versions of these decompositions, which we also note are still exact for *δ* ≥ 1. The depth-5 decomposition in Eq. (12) yields the shortest overall run-time when breaking down higher-weight interactions in a recursive fashion, assuming that the remaining three-local gates are decomposed using an expression similar to Eq. (11). We also carry out numerical studies that indicate that these decompositions are likely to be optimal. (See Supplementary Methods). These circuit decompositions allow us to establish that, for a weight-k interaction term, there exists a pulse sequence which implements the evolution operator for time *δ* with an overhead ∝ *δ*^1/(*k*−1)^, achieved by recursively applying these decompositions. While we have only made reference to interactions of the form *Z*^⊗*k*^, we remark that this is sufficient as we can obtain any other interaction term of the same weight, for example *ZXZ*, by conjugating *Z*^⊗*k*^ by single-qubit rotations, *H* and *SHS*^†^ in this example (where *H* is a Hadamard and *S* a phase gate).

**Fig. 1:**
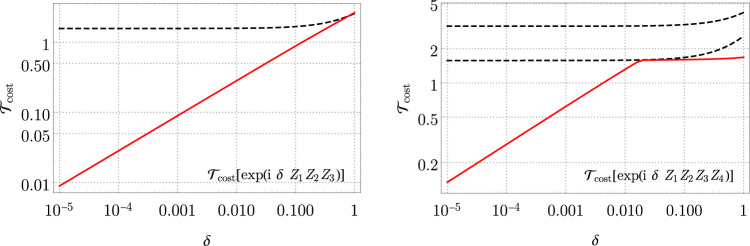
Gate decomposition cost ${{{{{{\mathcal{T}}}}}}}_{{{{{{\rm{cost}}}}}}}$ for decomposing $\exp ({{{{{\rm{i}}}}}}\delta {Z}^{\otimes 3})$ (left) and $\exp ({{{{{\rm{i}}}}}}\delta {Z}^{\otimes 4})$ (right), for *δ* ∈ [10^−5^, 1]. The lower dashed line is the cost obtained by conjugation decomposition, *π*/2 + *δ*. The upper dashed line is the cost for a once-nested conjgation, *π* + *δ*. Decomposing the four-local gate with an outer depth-5 and an inner depth 4 formula according to Eqs. (11) and (12) only saturates the lower conjugation cost bound.

For the interactions required for our Fermi-Hubbard simulation, the overhead of decomposing short-pulse gates with this analytic decomposition is $\propto \sqrt{\delta }$ for any weight-3 interaction term, and ∝*δ*^1/3^ for weight-4. The asymptotic run-time is thus ${{{{{\rm{O}}}}}}(T{\delta }_{0}^{w})$ for *w* = −1/2 (compact encoding) or *w* = − 2/3 (VC encoding). We show the exact scaling for *k* = 3 and *k* = 4 in Fig. 1, as compared to the standard conjugation method.

### Tighter error bounds for Trotter product formulas

There are by now a number of sophisticated quantum algorithms for Hamiltonian simulation, achieving optimal asymptotic scaling in some or all parameters^35–37^. Recently^38^, have shown that previous error bounds on Trotter product formulae were over-pessimistic. They derived new bounds showing that the older, simpler, product-formula algorithms achieve almost the same asymptotic scaling as the more sophisticated algorithms.

**Fig. 2:**
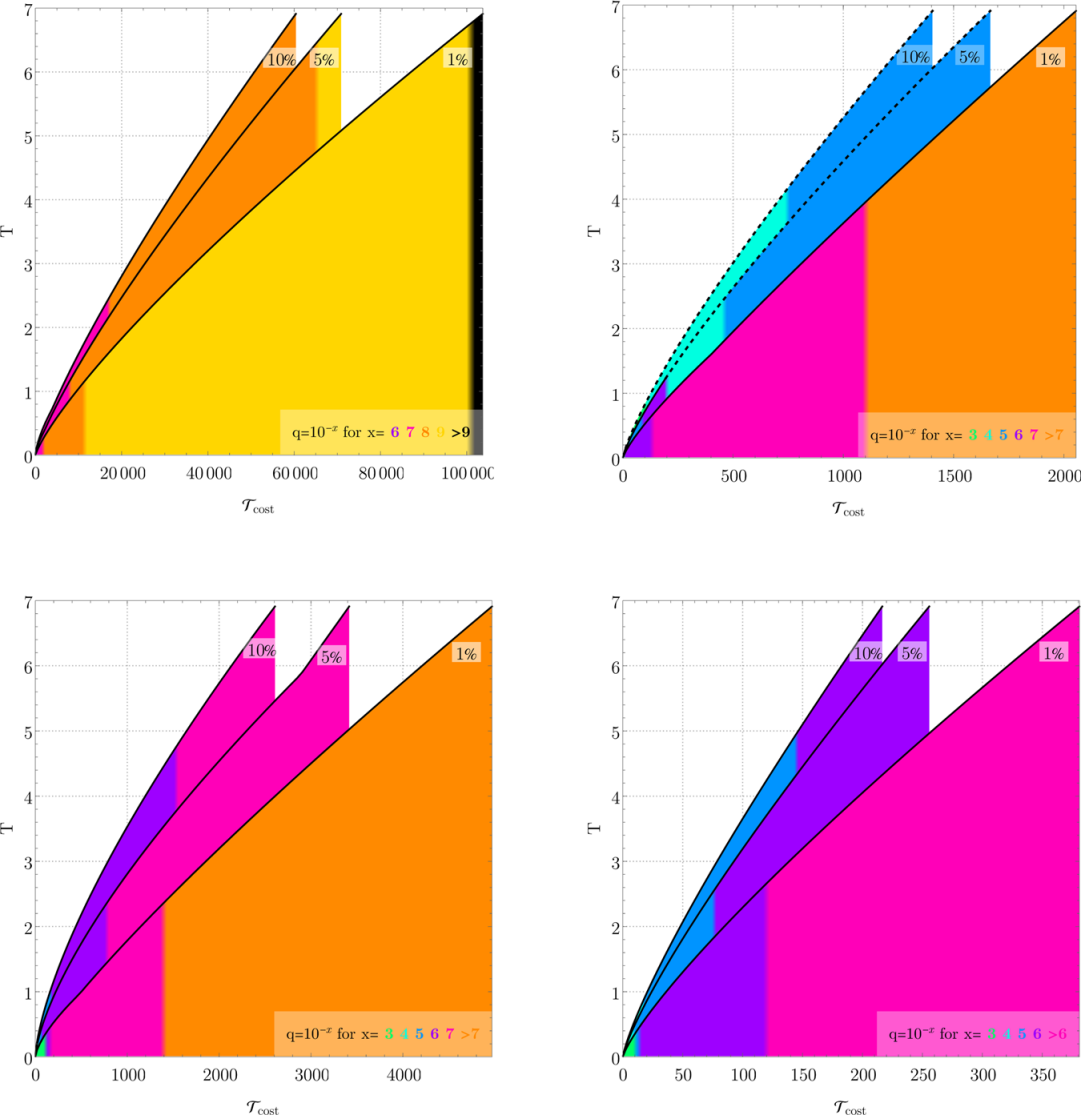
Target simulation time *T* vs cost ${{{{{{\mathcal{T}}}}}}}_{{{{{{\rm{cost}}}}}}}$ for a 5 × 5 lattice FH Hamiltonian *H*_FH_ from Supplementary Equation (139) using encoding of ref. ^25^. Per-gate (left column) and per-time (right column) error models. The three lines represent 1, 5 and 10% Trotter error *ϵ* given in Supplementary Eq. (43), minimised over formula order *p* ∈ {1, 2, 4, 6}. Analytic Trotter bounds (top row), get *δ*_0_ from Supplementary Corollaries 16 and 22 and Supplementary Theorem 20; numerical bounds (bottom row) by numerical extrapolation (see Supplementary Methods). Colours indicate achievable *T* for a given noise parameter *q*, keeping Trotter and depolarising errors below the 1, 5 or 10% bound, accordingly. E.g. the purple section of the bottom right 1% plot indicates that all *T* in that range needs *q* = 10^−6^, with Trotter and decoherence error below 1%. Dashed lines indicate where error mitigation from ref. ^25^ can reduce the noise requirements. Additional lattice sizes and details shown in Supplementary Figs. 16–19.

For near-term hardware, achieving good asymptotic scaling is almost irrelevant; what matters is minimising the actual circuit depth for the particular target system being simulated. Similarly, in the NISQ regime we do not have the necessary resources to implement full active error correction and fault tolerance. But we can still consider ways of minimising the output error probability for the specific computation being carried out. Simple product-formula algorithms allow good control of error propagation in the absence of active error correction and fault tolerance. Furthermore, combining product-formula algorithms with our circuit decompositions allows us to exploit the error-detection properties of fermionic encodings. We can use this to relax the effective noise rates required for accurate simulations, especially if we are willing to allow the simulation to include some degree of simulated natural noise. This is explained further the Supplementary Methods and the results of this technique are shown in Fig. 2.

For these reasons, we choose to implement the time-evolution operator $U(T):=\exp (-{{{{{\rm{i}}}}}}TH)$ by employing Trotter product formulae $U(T)=:{{{{{{\mathcal{P}}}}}}}_{p}{(\delta )}^{T/\delta }+{{{{{{\mathcal{R}}}}}}}_{p}(T,\delta )$. Here, ${{{{{{\mathcal{R}}}}}}}_{p}\left(T,\delta \right)$ denotes the error term remaining from the approximate decomposition into a product of individual terms, defined directly as ${{{{{{\mathcal{R}}}}}}}_{p}\left(T,\delta \right):=U(T)-{{{{{{\mathcal{P}}}}}}}_{p}{\left(\delta \right)}^{T/\delta }$. This includes the simple first-order formula^13^

13 $${{{{{{\mathcal{P}}}}}}}_{1}{\left(\delta \right)}^{T/\delta }:=\mathop{\prod }\limits_{n=1}^{T/\delta }\mathop{\prod }\limits_{i=1}^{M}{{{{{{\rm{e}}}}}}}^{-{{{{{\rm{i}}}}}}{H}_{i}\delta },$$

as well as higher-order variants^38–40^

14 $${{{{{{\mathcal{P}}}}}}}_{2}\left(\delta \right):=\mathop{\prod }\limits_{j=1}^{M}{{{{{{\rm{e}}}}}}}^{-{{{{{\rm{i}}}}}}{H}_{j}\delta /2}\mathop{\prod }\limits_{j=M}^{1}{{{{{{\rm{e}}}}}}}^{-{{{{{\rm{i}}}}}}{H}_{j}\delta /2},$$

15 $${{{{{{\mathcal{P}}}}}}}_{2k}\left(\delta \right):={{{{{{\mathcal{P}}}}}}}_{2k-2}{\left({a}_{k}\delta \right)}^{2}{{{{{{\mathcal{P}}}}}}}_{2k-2}\left((1-4{a}_{k})\delta \right){{{{{{\mathcal{P}}}}}}}_{2k-2}{\left({a}_{k}\delta \right)}^{2}$$

for $k\in {\mathbb{N}}$, where the coefficients are given by ${a}_{k}:=1/\left(4-{4}^{1/\left(2k-1\right)}\right)$. It is easy to see that, while for higher-order formulas not all pulse times equal *δ*, they still asymptotically scale as Θ(*δ*). The product formula ${{{{{{\mathcal{P}}}}}}}_{p}{\left(\delta \right)}^{T/\delta }$ then approximates a time evolution under *U*(*δ*)^*T*/*δ*^ ≈ *U*(*T*), and it describes the sequence of local unitaries to be implemented as a quantum circuit.

Choosing the Trotter step *δ* small means that corrections for every factor in this formula come in at ${{{{{\rm{O}}}}}}\left({\delta }^{p+1}\right)$ for $p\in \{1,2k:k\in {\mathbb{N}}\}$. Since we have to perform *T*/*δ* many rounds, the overall error scales roughly as ${{{{{\rm{O}}}}}}\left(T{\delta }^{p}\right)$. Yet this rough estimate is insufficient if we need to calculate the largest-possible *δ* for our Hamiltonian simulation.

The Hamiltonian dynamics remain entirely within one fermion number sector, as *H*_FH_ commutes with the total fermion number operator. Let Λ denote the number of fermions present in the simulation, such that ∥*H*_*i*_∣_Λ fermions_∥ ≤ Λ as shown in Supplementary Theorem 23. Let *M* = 5 denote the number of non-commuting Trotter layers, and set ${\epsilon }_{p}(T,\delta ):=\parallel {{{{{{\mathcal{R}}}}}}}_{p}(T,\delta )\parallel$, and as shorthand *ϵ*_*p*_(*δ*): = *ϵ*_*p*_(*δ*, *δ*), so that *ϵ*_*p*_(*T*, *δ*) = *T*/*δ* × *ϵ*_*p*_(*δ*).

To obtain a bound on ${{{{{{\mathcal{P}}}}}}}_{p}\left(\delta \right)$, we apply the variation of constants formula [ref. ^41^,Th, 4.9] to ${{{{{{\mathcal{R}}}}}}}_{p}(\delta )$, with the condition that ${{{{{{\mathcal{P}}}}}}}_{p}\left(0\right)={\mathbb{1}}$, which always holds. As in [ref. ^38^, sec. 3.2], for *δ* ≥ 0, we obtain

16 $${{{{{{\mathcal{P}}}}}}}_{p}\left(\delta \right)=U\left(\delta \right)+{{{{{{\mathcal{R}}}}}}}_{p}\left(\delta \right)={{{{{{\rm{e}}}}}}}^{-{{{{{\rm{i}}}}}}\delta H}+\int\nolimits_{0}^{\delta }{{{{{{\rm{e}}}}}}}^{-{{{{{\rm{i}}}}}}\left(\delta -\tau \right)H}{R}_{p}\left(\tau \right)d\tau $$

where the integrand ${R}_{p}\left(\tau \right)$ is defined as

17 $${R}_{p}\left(\tau \right):=\frac{d}{d\tau }{{{{{{\mathcal{P}}}}}}}_{p}\left(\tau \right)-\left(-{{{{{\rm{i}}}}}}H\right){{{{{{\mathcal{P}}}}}}}_{p}\left(\tau \right).$$

Now, if ${{{{{{\mathcal{P}}}}}}}_{p}\left(\delta \right)$ is accurate up to *p*th order—meaning that ${{{{{{\mathcal{R}}}}}}}_{p}\left(\delta \right)={{{{{\rm{O}}}}}}\left({\delta }^{p+1}\right)$—it holds that the integrand ${R}_{p}\left(\delta \right)={{{{{\rm{O}}}}}}\left({\delta }^{p}\right)$. This allows us to restrict its partial derivatives for all 0 ≤ *j* ≤ *p* − 1 to ${\partial }_{\tau }^{j}{R}_{p}\left(0\right)=0$. For full details see Supplementary Lemma 13 and [ref. ^38^, Order Conditions].

Then, following ref. ^38^, we perform a Taylor expansion of ${R}_{p}\left(\tau \right)$ around *τ* = 0, simplifying the error bound ${\epsilon }_{p}(\delta )\equiv \parallel {{{{{{\mathcal{R}}}}}}}_{p}(\delta )\parallel$ to

18 $${\epsilon }_{p}(\delta )=\left\Vert \int\nolimits_{0}^{\delta }{{{{{{\rm{e}}}}}}}^{-{{{{{\rm{i}}}}}}\left(\delta -\tau \right)H}{R}_{p}\left(\tau \right){{{{{\rm{d}}}}}}\tau \right\Vert \le \int\nolimits_{0}^{\delta }\parallel {R}_{p}\left(\tau \right)\parallel {{{{{\rm{d}}}}}}\tau $$

19 $$=\int\nolimits_{0}^{\delta }\left(\parallel {R}_{p}\left(0\right)\parallel +\parallel {R}_{p}^{\prime}\left(0\right)\parallel \tau +\ldots +\right.$$

20 $$\left.\parallel {R}_{p}^{\left(p-1\right)}\left(0\right)\parallel \frac{{\tau }^{p-1}}{\left(p-1\right)!}+\parallel {S}_{p}\left(\tau ,0\right)\parallel \right){{{{{\rm{d}}}}}}\tau .$$

Here we use the aforementioned order condition that for all 0 ≤ *j* ≤ *p* − 1 the partial derivatives satisfy ${\partial }_{\tau }^{j}{R}_{p}\left(0\right)=0$, leaving all but the *p*^th^ or higher remainder terms—${S}_{p}\left(\tau ,0\right)$—equal to zero. Thus

21 $${\epsilon }_{p}(\delta )	\le \int\nolimits_{0}^{\delta }\parallel {S}_{p}\left(\tau ,0\right)\parallel {{{{{\rm{d}}}}}}\tau \\ 	=p\int\nolimits_{0}^{\delta }\int\nolimits_{0}^{1}{\left(1-x\right)}^{p-1}\parallel {R}_{p}^{\left(p\right)}\left(x\tau \right)\parallel \frac{{\tau }^{p}}{p!}{{{{{\rm{d}}}}}}x{{{{{\rm{d}}}}}}\tau ,$$

where we used the integral representation for the Taylor remainder ${S}_{p}\left(\tau ,0\right)$.

Motivated by this, we look for simple bounds on the *p*th derivative of the integrand $\parallel {R}_{p}\left(\tau \right)\parallel$. At this point our work diverges from ref. ^38^ by focusing on obtaining bounds on $\parallel {R}_{p}\left(\tau \right)\parallel$, which have the tightest constants for NISQ-era system sizes, but which now are not optimal in system size. (See Supplementary Fig. 6 and Supplementary Lemmas 14 and 19 in Supplementary Methods for details.) We derive the following explicit error bounds (see Supplementary Theorem 15 and Supplementary Corollary 16):

22 $${\epsilon }_{p}(\delta )\le {\delta }^{p+1}{M}^{p+1}{{{\Lambda }}}^{p+1}{G}_{p}$$

where

23 $${G}_{p}:=\times \left\{\begin{array}{ll}\hskip-6pc1&p=1\hfill\\ \frac{2}{\left(p+1\right)!}{\left(\frac{10}{3}\right)}^{\left(p+1\right)\left(p/2-1\right)}&p=2k,k\ge 1,\end{array}\right.$$

and

24 $${\epsilon }_{p}\left(\delta \right)\le \frac{2{\delta }^{p+1}{M}^{p+1}{{{\Lambda }}}^{p+1}}{\left(p+1\right)!}{H}_{p}^{p+1}$$

where

25 $${H}_{p}:=\mathop{\prod }\limits_{i=1}^{p/2-1}\frac{4+{4}^{1/\left(2i+1\right)}}{\left|4-{4}^{1/\left(2i+1\right)}\right|}.$$

The above expressions hold for generic Trotter formulae. Using Supplementary Lemma 19 we can exploit commutation relations for the specific Hamiltonian at hand (whose structure determines *N* and *n*, see Supplementary Methods). This yields the bound (see Supplementary Theorem 20):

26 $${\epsilon }_{p}\left(\delta \right)\le {C}_{1}\frac{T{\delta }^{p}}{\left(p+1\right)!}+{C}_{2}\frac{T}{\delta }\int\nolimits_{0}^{\delta }p\int\nolimits_{0}^{1}{\left(1-x\right)}^{p-1}\frac{x{\tau }^{p+1}}{p!}{{{{{{\rm{e}}}}}}}^{x\tau N{B}_{p}}{{{{{\rm{d}}}}}}x{{{{{\rm{d}}}}}}\tau $$

where

27 $${C}_{1}:=\;	np{B}_{p}^{2}{{{\Lambda }}}^{p-1}N{\left(M{H}_{p}-{B}_{p}+{B}_{p}\left(\frac{N}{{{\Lambda }}}\right)\right)}^{p-1}\\ 	\times \left({\left({S}_{p}M\right)}^{2}-\left({S}_{p}M\right)\right),$$

28 $${C}_{2}:=n{B}_{p}^{2}{\left(M{H}_{p}{{\Lambda }}\right)}^{p}N\left({\left({S}_{p}M\right)}^{2}-\left({S}_{p}M\right)\right),$$

and

29 $${B}_{p}:=\left\{\begin{array}{ll}\hskip-5pc 1&p=1\hfill\\ \hskip-5pc\frac{1}{2}&p=2\hfill\\ \frac{1}{2}\mathop{\prod }\nolimits_{i = 2}^{k}(1-4{a}_{i})&p=2k,k\ge 2.\end{array}\right.$$

These analytic error bounds are then combined with a Taylor-of-Taylor method, by which we expand the Taylor coefficient ${R}_{p}^{(p)}$ in Eq. (21) itself in terms of a power series to some higher order *q* > *p*, with corresponding series coefficients ${R}_{p}^{(q)}$, and a corresponding remainder-of-remainder error term *ϵ*_*p*,*q*+1_. The tightest error expression we obtain is (see Supplementary Corollary 22 in Supplementary Methods)

30 $${\epsilon }_{p}(\delta )\le \mathop{\sum }\limits_{l=p}^{q}\frac{{\delta }^{l+1}{{{\Lambda }}}^{l+1}}{(l+1)!}f(p,M,l)+{\epsilon }_{p,q+1}(\delta ),$$

where the *f*(*p*, *M*, *l*) are exactly calculated coefficients (using a computer algebra package) that exploit cancellations between the *M* non-commuting Trotter layers, for a product formula of order *p* and series expansion order *l* (given in Supplementary Table 1). The series’ remainder *ϵ*_*p*,*q*+1_ therein is then derived from the analytic bounds in Eq. (26) (see Supplementary Methods for technical details).

Henceforth, we will assume the tightest choice of *ϵ*_*p*_(*δ*) among all the derived error expressions and choice of *p* ∈ {1, 2, 4}. In order to guarantee a target error bound *ϵ*_*p*_(*T*, *δ*) ≤ *ϵ*_target_, we invert these explicitly derived error bounds and obtain a maximum possible Trotter step *δ*_0_ = *δ*_0_(*ϵ*_target_).

### Benchmarking the sub-circuit-model

How significant is the improvement of the measures set out in previous sections, as benchmarked against state-of-the-art results from literature? A first comparison is in terms of exact asymptotic bounds (which we derive in Supplementary Corollaries 25 and 26), in terms of the number of non-commuting Trotter layers *M*, fermion number Λ, simulation time *T* and target error *ϵ*_target_:

Standard circuit synthesis:

31 $${{{{{{\mathcal{T}}}}}}}_{{{{{{\rm{cost}}}}}}}\left({{{{{{\mathcal{P}}}}}}}_{p}{(\delta )}^{T/\delta }\right)={{{{{\rm{O}}}}}}\left({M}^{2+\frac{1}{p}}{{{\Lambda }}}^{1+\frac{1}{p}}{T}^{1+\frac{1}{p}}{\epsilon }_{{{{{{\rm{target}}}}}}}^{-\frac{1}{p}}\right),$$

Sub-circuit synthesis:

32 $${{{{{{\mathcal{T}}}}}}}_{{{{{{\rm{cost}}}}}}}\left({{{{{{\mathcal{P}}}}}}}_{p}{(\delta )}^{T/\delta }\right)={{{{{\rm{O}}}}}}\left({M}^{\frac{3}{2}+\frac{1}{2p}}{{{\Lambda }}}^{\frac{1}{2}+\frac{1}{2p}}{T}^{1+\frac{1}{2p}}{\epsilon }_{{{{{{\rm{target}}}}}}}^{-\frac{1}{2p}}\right).$$

Here we write ${{{{{{\mathcal{T}}}}}}}_{{{{{{\rm{cost}}}}}}}$ for the "run-time” of the quantum circuits—i.e. the sum of pulse times of all gates within the circuit (See Supplementary Definition 3 for a detailed discussion of the cost model we employ).

Beyond asymptotic scaling, and in order to establish a more comprehensive benchmark that takes into account potentially large but hidden constant factors, we employ our tighter Trotter error bounds that account for all constant factors, and concretely target a 5 × 5 Fermi-Hubbard Hamiltonian for overall simulation time *T* = 7 (which is roughly the Lieb-Robinson time required for the "causality-cone” to spread across the whole lattice, and for correlations to potentially build up between any pair of sites), in the sector of Λ = 5 fermions, and coupling strengths ∣*u*∣, ∣*v*∣ ≤ *r* = 1 as given in Eq. (2). For this system, we choose the optimal Trotter product-formula order *p* that yields the lowest overall run-time, while still achieving a target error of *ϵ*_target_ = 0.1.

The results are given in Tables 1 and 2, where we emphasise that in order to maintain a fair comparison, we always account single-qubit gates as a free resource, for the reasons discussed in the Introduction, and two-qubit gates are either accounted at one unit of time per gate in the per-gate error model (making the run-time equal the circuit depth), or accounted at their pulse length for the per-time error model.

Our Trotter error bounds yield an order-of-magnitude improvement as compared to [ref. ^23^, Prop F.4]. And even for existing gate decompositions by conjugation, the recently published lower-weight compact encoding yields a small but significant improvement. The most striking advantage comes from utilising the sub-circuit sequence decompositions developed in this paper, in particular in conjunction with the lower-weight compact fermionic encoding.

Overall, the combination of Trotter error bounds, numerics, compact fermion encoding and sub-circuit-model algorithm design, allows us to improve the run-time of the simulation algorithm from 976,710 to 259—an improvement of more than three orders of magnitude over that obtainable using the previous state-of-the-art methods, and a further improvement over results in the pre-existing literature^15^.

### Sub-circuit algorithms on noisy hardware

As ours is a study of quantum simulation on near-term hardware, we cannot neglect decoherence errors that inevitably occur throughout the simulation. To address this concern, we assume an iid noise model described by the qubit depolarising channel

33 $${{{{{{\mathcal{N}}}}}}}_{q}(\rho )=(1-q)\rho +\frac{q}{3}\left(\right.X\rho X+Y\rho Y+Z\rho Z\left)\right.$$

applied to each individual qubit in the circuit, and after each gate layer in the Trotter product formula, such that the bit, phase, and combined bit-phase-flip probability *q* is proportional to the elapsed time of the preceding layer. While this standard error model is simplistic, it is a surprisingly good match to the errors seen in some hardware^6^.

Within this setting, a simple analytic decoherence error bound can readily be derived (see Supplementary Methods), by calculating the probability that zero errors appear throughout the circuit. If *V* denotes the volume of the circuit (defined as ${{{{{{\mathcal{T}}}}}}}_{{{{{{\rm{cost}}}}}}}\times {L}^{2}$), then the depolarising noise parameter $q \, < \, 1-{(1-{\epsilon }_{{{{{{\rm{target}}}}}}})}^{1/V}$—i.e. it needs to shrink exponentially quickly with the circuit’s volume. We emphasise that this is likely a crude overestimate. As briefly discussed at the start, one of the major advantages of sub-circuit circuits is that, under a short-pulse gate, an error is only weakly propagated due to the reduced Lieb-Robinson velocity (discussed further in ref. ^42^).

Yet irrespective of this overestimate, can we derive a tighter error bound by other means? In ref. ^42^, the authors analyse how noise on the physical qubits translates to errors in the fermionic code space. To first order and in the compact encoding, all of {*X*, *Y*, *Z*} errors on the face, and {*X*, *Y*} on the vertex qubits can be detected. *Z* errors on the vertex qubits result in an undetectable error, as evident from the form of *h*_on-site_ from Supplementary Equation (140). It is shown in [ref. ^42^, Sec. 3.2] that this *Z* error corresponds to fermionic phase noise in the simulated Fermi-Hubbard model.

It is therefore a natural extension to the notion of simulation to allow for some errors to occur, if they correspond to physical noise in the fermionic space. And indeed, as discussed more extensively in [ref. ^42^, Sec. 2.4], phase noise is a natural setting for many fermionic condensed matter systems coupled to a phonon bath^43–49^ and [ref. ^50^, Ch. 6.1&eq. 6.17].

How can we exploit the encoding’s error mapping properties? Under the assumption that *X*, *Y* and *Z* errors occur uniformly across all qubits, as assumed in Eq. (33), each Pauli error occurs with probability *q*/3. We further assume that we can measure all stabilizers (including a global parity operator) once at the end of the entire circuit, which can be done by dovetailing a negligible depth 4 circuit to the end of our simulation (see Supplementary Methods for more details). We then numerically simulate a stochastic noise model for the circuit derived from aforementioned Trotter formula for a specific target error *ϵ*_target_, for a Fermi-Hubbard Hamiltonian on an *L* × *L* lattice for *L* ∈ {3, 5, 10}.

Whenever an error occurs, we keep track of the syndrome violations they induce (including potential cancellations that happen with previous syndromes), using results from ref. ^42^ on how Pauli errors translate to error syndromes with respect to the fermion encoding’s stabilizers (summarised in Supplementary Table 2). We then bin the resulting circuit runs into the following categories:

1. Detectable error: at least one syndrome remains triggered, even though some may have canceled throughout the simulation,
2. Undetectable phase noise: no syndrome was ever violated, and the only errors are *Z* errors on the vertex qubits which map to fermionic phase noise, and
3. Undetectable non-phase noise: syndromes were at some point violated, but they all canceled.
4. Errors not happening in between Trotter layers: naturally, not all errors happen in between Trotter layers, so this category encompasses all those cases where errors happen in between gates in the gate decomposition.

This categorisation allows us to calculate the maximum depolarising noise parameter *q* to be able to run a simulation for time $T=\lfloor \sqrt{2}L\rfloor$ with target Trotter error *ϵ*_t_ ≤ *ϵ*_target_ ∈ {1%, 5%, 10%}, where we allow the resulting undetectable non-phase noise and the errors not happening in between Trotter layers errors to also saturate this error bound, i.e. *ϵ*_s_ ≤ *ϵ*_target_. The overall error is thus guaranteed to stay below a propagated error probability of ${({\epsilon }_{{{{{{\rm{t}}}}}}}^{2}+{\epsilon }_{s}^{2})}^{1/2}\in \{1.5 \% ,7.1 \% ,15 \% \}$, respectively.

In order to achieve these decoherence error bounds, one needs to postselect "good” runs and discard ones where errors have occurred, as determined from the single final measurement of all stabilizers of the compact encoding. The required overhead due to the postselected runs is mild, and shown in Supplementary Fig. 20.

We plot the resulting simulation cost vs. target simulation time in Fig. 2 and Supplementary Figs. 16–19 where we colour the graphs according to the depolarising noise rate required to achieve the target error bound. For instance, in the tightest per-time error model (bottom right plot in Fig. 2), a depolarising noise parameter *q* = 10^−5^ allows simulating a 5 × 5 FH Hamiltonian for time *T* ≈ 5, while satisfying a 15% error bound, the required circuit-depth-equivalent is ${{{{{{\mathcal{T}}}}}}}_{{{{{{\rm{cost}}}}}}}\approx 140$—and for time *T* ≈ 2.5 for a 7.1% error bound, for ${{{{{{\mathcal{T}}}}}}}_{{{{{{\rm{cost}}}}}}}\approx 70$.

In this work, we have derived a method for designing quantum algorithms "one level below” the circuit model, by designing analytic sub-circuit identities to decompose the algorithm into. As a concrete example, we applied these techniques to the task of simulating time-dynamics of the spin Fermi-Hubbard Hamiltonian on a square lattice. Together with Trotter product formulae error bounds applied to the recent compact fermionic encoding, we estimate these techniques provide a three orders of magnitude reduction in circuit-depth-equivalent. The authors of ref. ^51^ have recently extended their work on error bounds in ref. ^51^, beyond their results in ref. ^38^. We have not yet incorporated their new bounds into our analysis, and this may give further improvements over our analytic error bounds.

Naturally, any real world implementation on actual quantum hardware will allow and require further optimisations; for instance, all errors displayed within this paper are in terms of operator norm, which indicates the worst-case error deviation for any simulation. However, when simulating time-dynamics starting from a specific initial configuration and a distinct final measurement setup, a lower error rate results. We have accounted for this in a crude way, by analysing simulation of the Fermi-Hubbard model dynamics with initial states of bounded fermion number. But the error bounds—even the numerical ones—are certainly pessimistic for any specific computation. Furthermore, while we already utilise numerical simulations of Trotter errors, more sophisticated techniques such as Richardson extrapolation for varying Trotter step sizes might show promise in improving our findings further.

It is conceivable that other algorithms that require small unitary rotations will similarly benefit from designing the algorithms “one level below” the circuit model. Standard circuit decompositions of many interesting quantum algorithms will remain unfeasible on real hardware for some time to come. Whereas our sub-circuit-model algorithms, with their shorter overall run-time requirements and lower error propagation even in the absence of error correction, potentially bring these algorithms and applications within reach of near-term NISQ hardware.

## Supplementary information

Supplementary Information (pdf)

## Data Availability

The code to support the findings in this work is available upon request from the authors.
