# Supplementary material for: Hamiltonian simulation algorithms for near-term quantum hardware
Source: Nat Commun. 2021 Aug 17;12:4989. doi: 10.1038/s41467-021-25196-0 (PMC8371026; doi:10.1038/s41467-021-25196-0)
Supplement: Supplementary file 1 — Supplementary Information (pdf) [file 41467_2021_25196_MOESM1_ESM.pdf]

Supplementary Information

Laura Clinton *[1,2], Johannes Bausch †[1,3], and Toby Cubitt ‡[1]

[1]PhaseCraft Ltd.
[2]Department of Computer Science, University College London
[3]CQIF, DAMTP, University of Cambridge

November 2019

*laura@phasecraft.io
†johannes@phasecraft.io
‡toby@phasecraft.io

Supplementary Figures

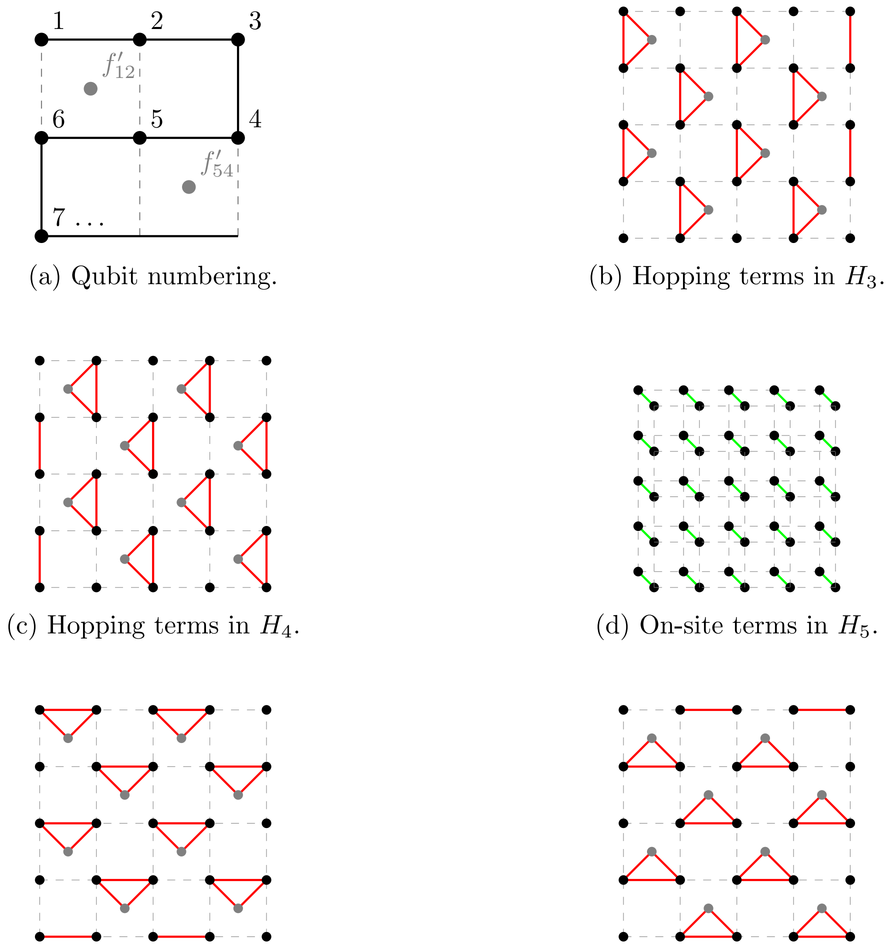

(a) Qubit numbering.

(b) Hopping terms in H_3.

(c) Hopping terms in H_4.

(d) On-site terms in H_5.

(e) Hopping terms in H_1.

(f) Hopping terms in H_2.

Supplementary Figure 1: Compact encoding: qubit enumeration, and five mutually non-commuting interaction layers.

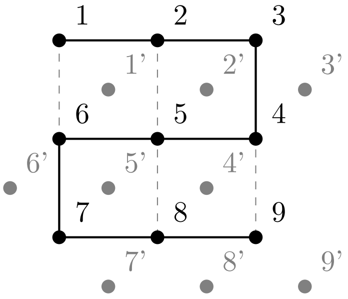

(a) Qubit numbering.

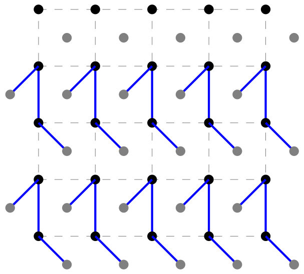

(b) Hopping terms in H_3.

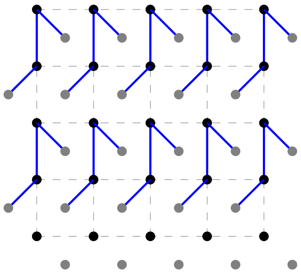

(c) Hopping terms in H_4.

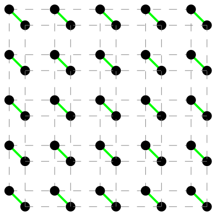

(d) On-site terms in H_5.

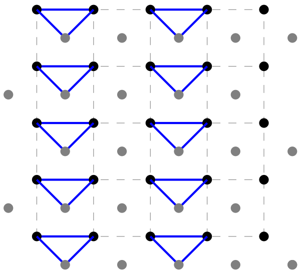

(e) Hopping terms in H_1.

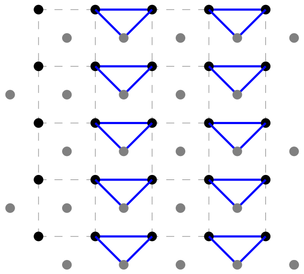

(f) Hopping terms in H_2.

Supplementary Figure 2: VC encoding: qubit enumeration, and five mutually non-commuting interaction layers.

Supplementary Methods

The Sub-Circuit Model and Error Models

In this section we introduce the sub-circuit model, which we employ throughout this paper. We analyse it under two different error models; these respective models are applicable to NISQ devices with differing capabilities. Before defining these we introduce the mathematical definition of the sub-circuit model.

Definition 1 (Sub-circuit Model). Given a set of qubits Q, a set $I \subseteq Q \times Q$ specifying which pairs of qubits may interact, a fixed two qubit interaction Hamiltonian h, and a minimum switching time t_{min}, a sub-circuit pulse-sequence C is a quantum circuit of L pairs of alternating layer types $C = \prod_l^L U_l V_l$ with $U_l = \prod_{i \in Q} u_i^l$ being a layer of arbitrary single qubit unitary gates, and $V_l = \prod_{ij \in \Gamma_l} v_{ij}\left(t_{ij}^l\right)$ being a layer of non-overlapping, variable time, two-qubit unitary gates:

$$v_{ij}(t) = \mathrm{e}^{\mathrm{i}th_{ij}} \tag{1}$$

with the set $\Gamma_l \subseteq I$ containing no overlapping pairs of qubits, and $t \geq t_{min}$. Throughout this paper we assume $h_{ij} = Z_i Z_j$. As all $\sigma_i \sigma_j$ are equivalent to $Z_i Z_j$ up to single qubit rotations this can be left implicit and so we take $h_{ij} = \sigma_i \sigma_j$.

The traditional quantum circuit model measures its run-time in layer count. This also applies in the sub-circuit-model.

Definition 2 (Circuit Depth). Under a per-gate error model the cost of a sub-circuit pulse-sequence C is defined as

$$\mathcal{T}_{\text{cost}}(C) := L, \tag{2}$$

or simply the circuit depth.

However, unlike the traditional quantum circuit model, the sub-circuit-model also allows for a different run-time metric for any given circuit C. Depending on the details of the underlying hardware, it can be appropriate to measure run-time as the total physical duration of the two-qubit interaction layers. This is justified for many implementations: for example superconducting qubits have interaction time scales of $\sim 50 - 500$ns [1], while the single qubit energy spacing is on the order of ~ 5Ghz, which gives a time scale for single qubit gates of ~ 0.2ns.

Definition 3 (Run-time). The physical run-time of a sub-circuit pulse-sequence C is defined as

$$\mathcal{T}_{\text{cost}}(C) := \sum_l^L \max_{ij \in \Gamma_l} \left(t_{ij}^l\right) \tag{3}$$

The run-time is normalised to the physical interaction strength, so that $|h| = 1$.

For both run-time and circuit depth we assume single qubit layers contribute a negligible amount to the total time duration of the circuit and we can cost standard gates according to both metrics as long as they are written in terms of a sub-circuit pulse-sequence. For example, according to Supplementary Definition 3 a CNOT gate has $\mathcal{T}_{\text{cost}} = \pi/4$ as it is equivalent to $e^{-i\frac{\pi}{4}ZZ}$ up to single qubit rotations.

How does this second cost model affect the time complexity of algorithms? I.e., given a circuit C, does $\mathcal{T}_{\text{cost}}(C)$ ever deviate so significantly from C's gate depth count that the circuit would have to be placed in a complexity class lower? Under reasonable assumptions on the shortest pulse time we prove in the following that this is not the case.

Remark 4. Let $\{C_x\}_{x\in\mathbb{N}}$ be a family of quantum circuits ingesting input of size x. Denote with $m(x)$ the circuit depth of C_x; and let $\delta_0 = \delta_0(x) := \min_{l\in[L]} \max_{ij\in\Gamma_l}(\tau_{ij}^t)$ be the shortest layer time pulse present in the circuit C_x, according to Supplementary Definition 3. Then if

$$\delta_0(x) = \begin{cases} \mathrm{O}(1) \\ 1/\operatorname{poly} x \\ 1/\exp(\operatorname{poly} x) \end{cases} \quad \Longrightarrow \quad m(x) = \mathcal{T}_{\text{cost}}(C) \times \begin{cases} \mathrm{O}(1) \\ \mathrm{O}(\operatorname{poly} x) \\ \mathrm{O}(\exp(\operatorname{poly} x)) \end{cases} \tag{4}$$

Furthermore $\mathcal{T}_{\text{cost}}(C) = \mathrm{O}(m(x))$.

Proof. Clear since $m(x) = \mathrm{O}(\mathcal{T}_{\text{cost}}(C)/\delta_0(x))$. The second claim is trivial.

An immediate consequence of using the cost model metric and the overhead of counting gates from Supplementary Remark 4 can be summarised as follows.

Corollary 5. Let $\epsilon > 0$. Any family of short-pulse circuits $\{C_x\}$ with $\delta_0(x) = \mathrm{O}(1)$ can be approximated by a family of circuits $\{\tilde{C}_x\}$ made up of gates from a fixed universal gate set; and such that $\tilde{C}_x$ approximates C_x in operator norm to precision ϵ in time $\mathrm{O}(\log^4(\mathcal{T}_{\text{cost}}(C_x)/\epsilon))$.

Proof. By Supplementary Remark 4, there are $m(x) = \mathcal{T}_{\text{cost}}(C) \times \mathrm{O}(1)$ layers of gates in C; now apply Solovay-Kitaev to compile it to a universal target gate set.

Indeed, we can take this further and show that complexity classes like BQP are invariant under an exchange of the two metrics "circuit depth" and "$\mathcal{T}_{\text{cost}}$"; if e.g. $\delta_0(x) = 1/\operatorname{poly} x$, then again invoking Solovay-Kitaev lets one upper-bound and approximate any circuit while only introducing an at most poly-logarithmic overhead in circuit depth. However, a stronger result than this is already known, independent of any lower bound on pulse times, which we cite here for completeness.

Remark 6 (Poulin et. al. [2]). A computational model based on simulating a local Hamiltonian with arbitrarily-quickly varying local terms is equivalent to the standard circuit model.

Sub-Circuit Synthesis of Multi-Qubit Interactions

Analytic Pulse Sequence Identities

In this section we introduce the analytic pulse sequence identities we use to decompose local Trotter steps $\mathrm{e}^{-\mathrm{i}\delta h}$. Their recursive application allows us to establish, that for a k-qubit Pauli interaction h, there exists sub-circuit pulse-sequence $C := \prod_l^L U_l V_l$ which implements the evolution operator $\mathrm{e}^{-\mathrm{i}\delta h}$. Most importantly, for any target time $\delta \geq 0$ the run-time of that circuit is bounded as

$$\mathcal{T}_{\mathrm{cost}}\left(C\right) \leq \mathcal{O}\left(\delta^{\frac{1}{k-1}}\right), \tag{5}$$

according to the notion of run-time established in Supplementary Definition 3.

For $k = 2^n + 1$ where $n \in \mathbb{Z}$ as noted by [3], this can be done inexactly using a well know identity from Lie algebra. For Hermitian operators A and B we have

$$\mathrm{e}^{-\mathrm{i}tB}\mathrm{e}^{-\mathrm{i}tA}\mathrm{e}^{\mathrm{i}tB}\mathrm{e}^{\mathrm{i}tA} = \mathrm{e}^{t^2[A,B]} + \mathcal{O}\left(t^3\right). \tag{6}$$

We make this exact for all $t \in [0, 2\pi]$ for anti-commuting Pauli interactions in Supplementary Lemma 7, and use Supplementary Lemma 8 to extend it to all $k \in \mathbb{Z}$.

Lemma 7 (Depth 4 Decomposition). Let $U(t) = \mathrm{e}^{\mathrm{i}tH}$ be the time-evolution operator for time t under a Hamiltonian $H = \frac{1}{2\mathrm{i}}[h_1, h_2]$, where h_1 and h_2 anti-commute and both square to identity. For $0 \leq \delta \leq \pi/2$ or $\pi \leq \delta \leq 3\pi/2$, $U(t)$ can be decomposed as

$$U(t) = \mathrm{e}^{\mathrm{i}t_1 h_1}\mathrm{e}^{\mathrm{i}t_2 h_2}\mathrm{e}^{\mathrm{i}t_2 h_1}\mathrm{e}^{\mathrm{i}t_1 h_2} \tag{7}$$

with pulse times t_1, t_2 given by

$$t_1 = \frac{1}{2}\tan^{-1}\left(\frac{1}{\sin(t)+\cos(t)},\ \pm\frac{\sqrt{\sin(2t)}}{\sin(t)+\cos(t)}\right) + \pi c \tag{8}$$

$$t_2 = \frac{1}{2}\tan^{-1}\left(\cos(t)-\sin(t),\ \mp\sqrt{\sin(2t)}\right) + \pi c, \tag{9}$$

where $c \in \mathbb{Z}$, and corresponding signs are taken in the two expressions.

For $\pi/2 \leq t \leq \pi$ or $3\pi/2 \leq t \leq 2\pi$, $U(t)$ can be decomposed as

$$U(t) = \mathrm{e}^{\mathrm{i}t_1 h_1}\mathrm{e}^{\mathrm{i}t_2 h_2}\mathrm{e}^{-\mathrm{i}t_2 h_1}\mathrm{e}^{-\mathrm{i}t_1 h_2} \tag{10}$$

with pulse times t_1, t_2 given by

$$t_1 = \frac{1}{2}\tan^{-1}\left(\frac{1}{\cos(t)-\sin(t)},\ \pm\frac{\sqrt{-\sin(2t)}}{\cos(t)-\sin(t)}\right) + \pi c \tag{11}$$

$$t_2 = \frac{1}{2}\tan^{-1}\left(\sin(t)+\cos(t),\ \pm\sqrt{-\sin(2t)}\right) + \pi c, \tag{12}$$

where $c \in \mathbb{Z}$, and corresponding signs are taken in the two expressions.

Proof. Follows similarly to Supplementary Lemma 27.

Lemma 8 (Depth 5 Decomposition). Let $U(t)$ be the time-evolution operator for time t under a Hamiltonian $H = \frac{1}{2\mathrm{i}}[h_1, h_2]$. If h_1 and h_2 anti-commute and both square to identity, then $U(t)$ can be decomposed as

$$U(t) = \mathrm{e}^{\mathrm{i}t_1 h_2}\mathrm{e}^{-\mathrm{i}\phi h_1}\mathrm{e}^{\mathrm{i}t_2 h_2}\mathrm{e}^{\mathrm{i}\phi h_1}\mathrm{e}^{\mathrm{i}t_1 h_2} \tag{13}$$

with pulse times t_1, t_2, ϕ given by

$$t_1 = \frac{1}{2}\tan^{-1}\left(\pm\sqrt{2}\sec(t)\csc(2\phi)\sqrt{\cos(2t) - \cos(4\phi)},\ -2\tan(t)\cot(2\phi)\right) + \pi c \tag{14}$$

$$t_2 = \tan^{-1}\left(\pm\frac{\csc(2\phi)\sqrt{\cos(2t) - \cos(4\phi)}}{\sqrt{2}},\ \sin(t)\csc(2\phi)\right) + 2\pi c \tag{15}$$

where $c \in \mathbb{Z}$, and corresponding signs are taken in the two expressions.

Proof. Follows similarly to Supplementary Lemma 27.

Pulse-Time Bounds on Analytic Decompositions

In our later analysis we apply these methods to the interactions in the Fermi-Hubbard Hamiltonian. Depending on the fermionic encoding used, these interaction terms are at most 3-local or 4-local. Supplementary Figure 3 depicts exactly how Supplementary Lemmas 7 and 8 are used to decompose 3-local and 4-local interactions of the form $Z^{\otimes k}$.

We establish bounds on the run-time (Supplementary Definition 3) of these circuits. The exact run-time of the circuit C_a – defined in Supplementary Figure 3a – follows directly from Supplementary Definition 3 as

$$\mathcal{T}_{\text{cost}}(C_a(t)) = 2|t_1^a(t)| + 2|t_2^a(t)|. \tag{16}$$

We have labelled the functions $t_i(t)$ from Supplementary Lemma 7 as $t_i^a(t)$ in order to distinguish them from those given in Supplementary Lemma 8, which are now labelled $t_i^b(t)$. This is to avoid confusion when using both identities in the one circuit, such as in circuit C_b where we use Supplementary Lemma 7 to decompose the remaining 3-local gates.

The exact run-time of the circuit C_b – defined in Supplementary Figure 3b – is left in terms of $\mathcal{T}_{\text{cost}}(C_a)$ and again follows directly from Supplementary Definition 3 as

$$\mathcal{T}_{\text{cost}}(C_b(t,\phi)) = 2\,\mathcal{T}_{\text{cost}}(C_a(t_1^b(t,\phi))) + \mathcal{T}_{\text{cost}}(C_a(t_2^b(t,\phi))) + 2|\phi|. \tag{17}$$

Supplementary Lemmas 9 and 10 bound these two functions and determine the optimal choice of the free pulse-time ϕ. Inserting these bounds into the above $\mathcal{T}_{\text{cost}}$ expressions gives

$$\mathcal{T}_{\text{cost}}(C(t)) \leq \begin{cases} 2\sqrt{2t} & C = C_a \\ 7\sqrt[3]{t} & C = C_b \end{cases}. \tag{18}$$

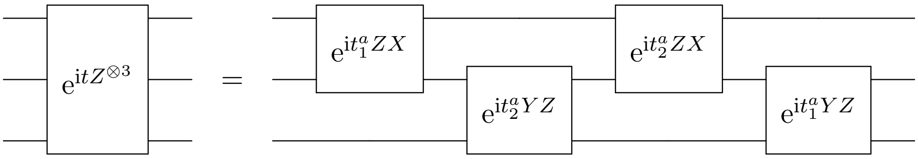

(a) Circuit C_a. The pulse times $t_i^a(t)$ are defined in Supplementary Lemma 7 and the run-time is bounded as $\mathcal{T}_{\text{cost}}(C_a(t)) \leq 2\sqrt{2t}$.

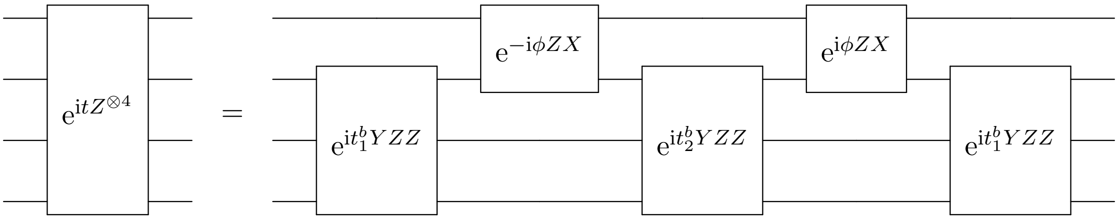

(b) Circuit C_b. The pulse times $t_i^b(t)$ are defined in Supplementary Lemma 8. The three local gates are further decomposed using Supplementary Lemma 7, though this is not shown here. If $\phi = \left(\frac{1}{4}(3+2\sqrt{2})t\right)^{1/3}$ then the run-time is bounded as $\mathcal{T}_{\text{cost}}(C_b(t)) \leq 7\sqrt[3]{t}$.

Supplementary Figure 3: The definitions of circuits $C_a(t)$ and $C_b(t)$ – which respectively generate evolution under a three and four local Pauli interaction for target time $t \geq 0$.

As $Z^{\otimes k}$ is equivalent to any k-local Pauli term up to single qubit rotations, these bounds hold for any three or four local Pauli interactions.

Lemma 9. Let H be as in Supplementary Lemma 7. For $0 \leq t \leq \pi/2$, the pulse times t_i in Supplementary Lemma 7 can be bounded by

$$|t_1| \leq \sqrt{\frac{t}{2}} \tag{19}$$

$$|t_2| + |t_1| \leq \sqrt{2t}. \tag{20}$$

Proof. Choosing the negative $t_1(t)$ and corresponding $t_2(t)$ solution from Supplementary Lemma 7 and Taylor expanding about $t = 0$ gives

$$t_1(t) = -\sqrt{\frac{t}{2}} + R_1(t) \tag{21}$$

$$t_2(t) = \sqrt{\frac{t}{2}} + R_2(t). \tag{22}$$

Basic calculus shows that t_1 is always negative and t_2 is always positive for $0 \leq t \leq \pi/2$, thus

$$|t_2| + |t_1| = t_2 - t_1 = \sqrt{2t} + R_{12}(t). \tag{23}$$

Then it can be shown that the Taylor remainders R_1 and R_{12} are positive and negative, respectively, giving the stated bounds.

Lemma 10. Let H be as in Supplementary Lemma 8. For $0 \le t \le t_c$ and $\phi = (ct)^{1/3}$, the pulse times t_i in Supplementary Lemma 8 can be bounded by

$$2\sqrt{2|t_2|} + 4\sqrt{2|t_1|} + 2|\phi| \le 3(6+4\sqrt{2})^{1/3} t^{1/3} \tag{24}$$
$$\le 7t^{1/3}, \tag{25}$$

where $c = \frac{1}{4}(3+2\sqrt{2})$ and $t_c \sim 0.33$.

Proof. This follows similarly to Supplementary Lemma 9. We choose the positive branch of the $\pm$ solutions for pulse times with $t_1(t)$ and $t_2(t)$ given in Supplementary Lemma 8, and freely set $\phi = (ct)^{1/3}$ for some positive constant $c \in \mathbb{R}$. Within the range $0 \le t \le t_c$ we have real pulse times $t_1 \le 0$ and $t_2 \ge 0$. We can then Taylor expand the following about $t = 0$ to find

$$2\sqrt{2|t_2|} + 4\sqrt{2|t_1|} + 2|\phi| = 2\sqrt{2t_2} + 4\sqrt{-2t_1} + 2(ct)^{1/3} \tag{26}$$
$$= \frac{2\left(\sqrt{c}+\sqrt{2}+1\right)}{\sqrt[6]{c}} t^{1/3} + R(t). \tag{27}$$

Choosing c to minimise the first term in this expansion, and again showing that $R \le 0$, leads to the stated result

$$2\sqrt{2|t_2|} + 4\sqrt{2|t_1|} + 2|\phi| \le 3(6+4\sqrt{2})^{1/3} t^{1/3} \tag{28}$$
$$\le 7t^{1/3} \tag{29}$$

where $\phi = (ct)^{1/3}$ and $c = \frac{1}{4}(3+2\sqrt{2})$. This is valid only with the region $0 \le t \le t_c$ where $t_c \approx 0.33$.

Theorem 11. For a set of qubits Q, a set $I \subseteq Q \times Q$ specifying which pairs of qubits may interact, and a fixed two qubit interaction Hamiltonian h_{ij}, if H is a k-body Pauli Hamiltonian then the following holds:

For all t there exists a quantum circuit of L pairs of alternating layer types $C = \prod_l^L U_l V_l$ with $U_l = \prod_{i\in Q} u_i^l$ being a layer of arbitrary single qubit unitary gates, and $V_l = \prod_{ij\in\Gamma_l} v_{ij}\left(t_{ij}^l\right)$ being a layer of non-overlapping, variable time, two-qubit unitary gates $v_{ij}(t) = \mathrm{e}^{\mathrm{i}th_{ij}}$ with the set $\Gamma_l \subseteq I$ containing no overlapping pairs of qubits such that $C = \mathrm{e}^{\mathrm{i}tH}$ and

$$\mathcal{T}_{\text{cost}}(C) \le \mathrm{O}\left(|t|^{\frac{1}{k-1}}\right), \tag{30}$$

where

$$\mathcal{T}_{\text{cost}}(C) := \sum_l^L \max_{ij\in\Gamma_l}\left(t_{ij}^l\right). \tag{31}$$

Proof. The proof of this claim follows from first noting that for any $t < 0$ one can conjugate $\mathrm{e}^{-\mathrm{i}tH}$ by a single Pauli operator which anti-commutes with H in order to obtain $\mathrm{e}^{\mathrm{i}tH}$. Therefore we can consider w.l.o.g. we can take $t > 0$ as we have done up until now.

The sub-circuit C which implements $\mathrm{e}^{\mathrm{i}tH}$ is constructed recursively using the Depth 5 decomposition. We note that the Depth 5 decomposition has an important feature. The free choice of ϕ allows us to avoid incurring a fixed root overhead with every iterative application of this decomposition. That is when using it to decompose any $\mathrm{e}^{\mathrm{i}tZ^{\otimes k}}$, we can always choose h_1 as a 2-local interaction and h_2 as a $(k-1)$-local interaction. We can choose $\phi \propto t^{\frac{1}{k-1}}$ and a similar analysis as in Supplementary Lemma 10 will show that this leaves the remaining pulse-times as $t_i \propto t^{1-\frac{1}{k-1}}$. This can be iterated to decompose the remaining gates, all of the form of evolution under $(k-1)$-local interactions for times $\propto t^{1-\frac{1}{k-1}}$. At each iteration we choose to h_1 as a 2-local interaction and $\phi \propto t^{\frac{1}{k-1}}$. Hence after $k-2$ iterations we will have established the claim that $\mathcal{T}_{\mathrm{cost}}(C) \leq \mathrm{O}\left(|t|^{\frac{1}{k-1}}\right)$.

Optimality

An obvious question to ask at this point is whether the proposed decompositions are optimal, in the sense that they minimise the total run-time $\mathcal{T}_{\mathrm{cost}}$ while reproducing the target gate h exactly. A closely related question is then whether relaxing the condition that we want to simulate the target gate without any error allows us to reduce the scaling of $\mathcal{T}_{\mathrm{cost}}$ with regards to the target time δ.

In this section we perform a series of numerical studies which indicate that the exact decompositions described in this section are indeed optimal within some parameter bounds, and that relaxing the goal to approximate implementations gives no benefit.

The setup is precisely as outlined in before: for $U_{\mathrm{target}} = \exp(\mathrm{i}TZ^{\otimes k})$ for some locality $k > 1$ and time $T > 0$, we iterate over all possible gate sequences of width k and length n, the set of which we call $U_{n,k}$. For each sequence $U \in U_{n,k}$, we perform a grid search over all parameter tuples $(t_1, \ldots, t_n) \in [-\pi/2, \pi/2]^n$ and $\delta \in [0, \pi/10]$, and calculate the parameter tuple $(\epsilon(U), \mathcal{T}_{\mathrm{cost}})$, where $\mathcal{T}_{\mathrm{cost}}$ is given in Supplementary Definition 3, and

$$\epsilon(U) := \|U - U_{\mathrm{target}}\|_2 \,. \tag{32}$$

The results are binned into brackets over $(\delta, \mathcal{T}_{\mathrm{cost}}) \in [\pi/10, n\pi/2]$ and their minimum within each bracket is taken. This procedure yields two outcomes:

1. For each target time δ and each target error $\epsilon > 0$, it yields the smallest $\mathcal{T}_{\mathrm{cost}}$, depth n circuit with error less than ϵ, and

2. for each target time δ and each $\mathcal{T}_{\mathrm{cost}}$, the smallest error possible with any depth n gate decomposition and total pulse time less than $\mathcal{T}_{\mathrm{cost}}$.

This algorithm scales exponentially both in k and n, and polynomial in the number of grid search subdivisions. The following optimisations were performed.

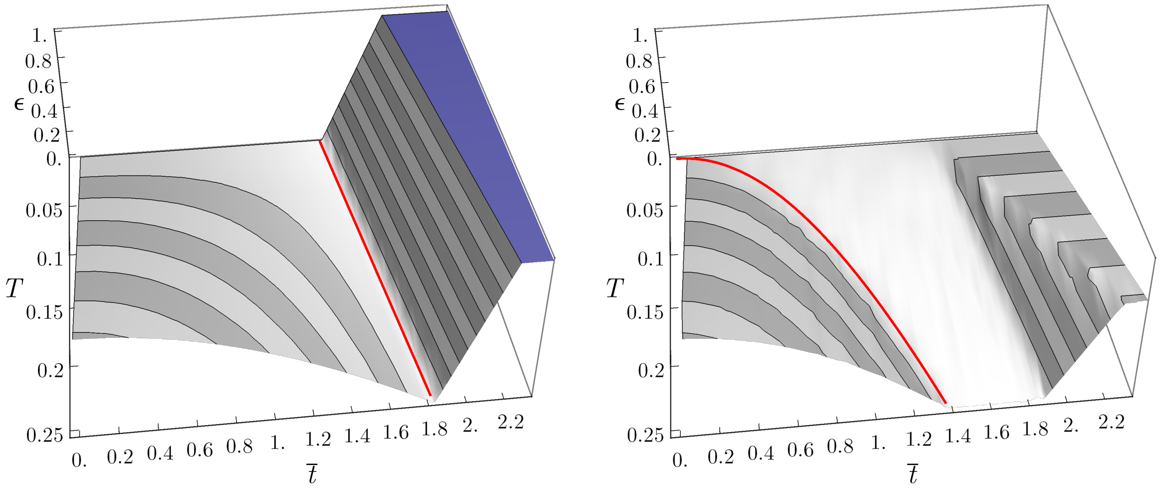

Supplementary Figure 4: Numerical calculation of gate decomposition errors of the $U_{\text{target}} = \exp(\mathrm{i}TZ^{\otimes 3})$ gate, with a pulse sequence of depth 3 (left) and depth 4 (right). Plotted in red are the optimal analytical decompositions given by CNOT conjugation and Supplementary Lemma 7, respectively.

1. We remove duplicate gate sequences under permutations of the qubits (since U_{target} is permutation symmetric).
2. We restrict ourselves to two-local Pauli gates, since any one-local gate can always be absorbed by conjugations, and
3. We remove mirror-symmetric sequences (since Paulis are self-adjoint).
4. For $n > 4$ we switch to performing a random sampling algorithm instead of grid search, since the number of grid points becomes too large.

Results for $k = 3$ and $n = 3, 4, 5$ are plotted in Supplementary Figures 4 and 5. As can be seen (plotted as red line), for $n = 3$ the optimal zero-error decomposition has $\mathcal{T}_{\text{cost}} = \pi + \delta$ from CNOT conjugation. For $n = 4$, the optimal decomposition is given by the implicitly-defined solution in Supplementary Lemma 7, with a $\mathcal{T}_{\text{cost}} \propto \sqrt{\delta}$ dependence. For the depth 5 sequences, it appears that the same optimality as for depth 4 holds. In contrast to $n = 3$ and $n = 4$, there is now a zero error solution for all $\mathcal{T}_{\text{cost}}$ greater than the optimum threshold.

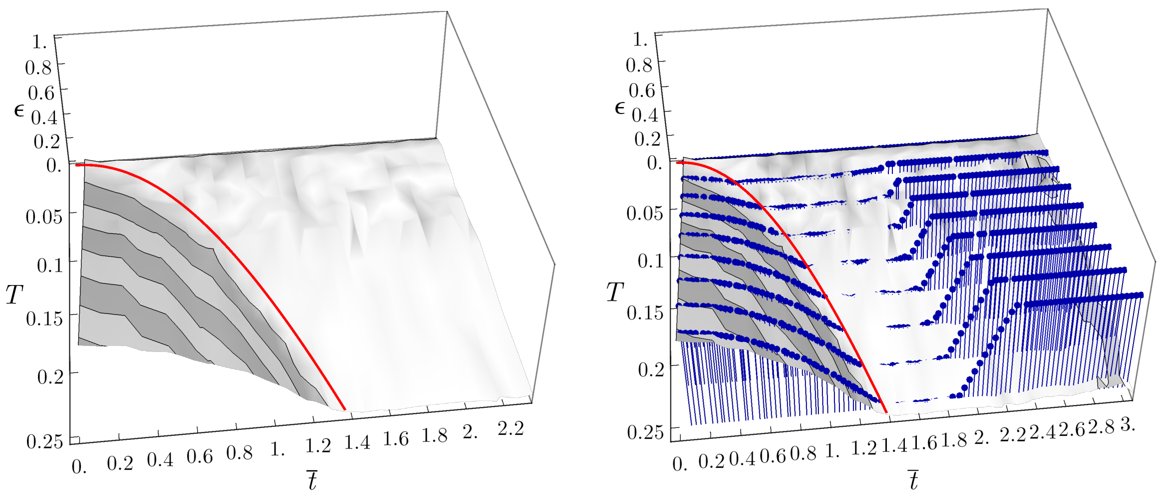

Supplementary Figure 5: Numerical calculation of gate decomposition errors of the $U_{\text{target}} = \exp(iTZ^{\otimes 3})$ gate, with a pulse sequence of depth 5. Plotted in red is the optimal analytical decompositions given for a depth 4 sequence in Supplementary Lemma 7; the blue lines are an overlay over the optimal depth 4 sequences from Supplementary Figure 4.

Suzuki-Trotter Formulae Error Bounds

Existing Trotter Bounds

Trotter error bounds have seen a spate of dramatic and very exciting improvements in the past few years [4, 5, 6]. However, among these recent improvements we could not find a bound that was exactly suited to our purpose.

We wanted bounds which took into account the commutation relations between interactions in the Hamiltonian, as we know this leads to tighter error bounds [5] [4]. However, we needed exact constants in the error bound when applied to 2D lattice Hamiltonians, such as the 2D Fermi-Hubbard model. For this reason we could not directly apply the results of [5] which only explicitly obtains constants for 1D lattice Hamiltonians.

Additionally, we needed to be able to straightforwardly compute the bound for any higher order Trotter formula. This ruled out using the commutator bounds of [4] as they become difficult to compute at higher orders. Furthermore these bounds require each Trotter layer to consist of a single interaction, meaning we wouldn't be able to exploit the result of Supplementary Theorem 23.

We followed the notation and adapted the methods of [5] to derive bounds that meet the above criteria. Additionally we incorporate our own novel methods to tighten our bounds in Supplementary Corollaries 16 and 22 and Supplementary Theorem 23.

The authors of [5] have recently extended their work further in [6]. We have not yet seen whether they will further tighten our analysis, though we are keen to do this in future work.

Hamiltonian Simulation by Trotterisation

In this section we derive our bounds for Trotter error. The standard approach to implementing time-evolution under a local Hamiltonian $H = \sum_i h_i$ on a quantum computer is to "Trotterise" the time evolution operator $U(T) = e^{-iHT}$. Assuming that the Hamiltonian breaks up into M mutually non-commuting layers $H = \sum_{i=1}^{M} H_i$ – i.e. such that $\forall i \neq j\, [H_i, H_j] \neq 0$ – Trotterizing in its basic form means expanding

$$U(T) := \mathrm{e}^{-\mathrm{i}HT} = \prod_{n=1}^{T/\delta} \prod_{i=1}^{M} \mathrm{e}^{-\mathrm{i}H_i\delta} + \mathcal{R}_1(T,\delta) = \mathcal{P}_1(\delta)^{T/\delta} + \mathcal{R}_1(T,\delta) \tag{33}$$

and then implementing the approximation $\mathcal{P}_1(\delta)^{T/\delta}$ as a quantum circuit. Here $\mathcal{R}_1(T,\delta)$ denotes the error term remaining from the approximate decomposition into a product of individual terms. $R_1(T,\delta) := U(T) - P_1(\delta)^{T/\delta}$ is simply defined as the difference to the exact evolution $U(T)$. For M mutually non-commuting layers of interactions H_i, we must perform M sequential layers per Trotter step.

Supplementary Equation (33) is an example of a first-order product formula, and is derived from the Baker-Campbell-Hausdorff identity

$$\mathrm{e}^{A+B} = \mathrm{e}^{A}\mathrm{e}^{B}\mathrm{e}^{[A,B]/2}\cdots \quad \text{and} \quad \mathrm{e}^{A+B} = \mathrm{e}^{(\delta A+\delta B)/\delta} = \left[\mathrm{e}^{\delta A+\delta B}\right]^{1/\delta}. \tag{34}$$

Choosing δ small in Supplementary Equation (33) means that corrections for every factor in this formula come in at $\mathrm{O}\left(\delta^2\right)$ i.e. in the form of a commutator, and since we have to perform $1/\delta$ many rounds of the sequence $\mathrm{e}^{\delta A}\mathrm{e}^{\delta B}$ the overall error scales roughly as $\mathrm{O}(\delta)$.

Since its introduction in [7], there have been a series of improvements, yielding higher-order expansions with more favourable error scaling. For a historical overview of the use of Suzuki-Trotter formulas in the context of Hamiltonian simulation, we direct the reader to the extensive overview given in [8, sec. 2.2.1]. In the following, we discuss the most recent developments for higher order product formulas, and analyse whether they yield an improved overall time and error scaling with respect to our introduced cost model.

To obtain higher-order expansions, Suzuki et. al. derived an iterative expression for product formulas in [9, 10]. For the $(2k)^{\text{th}}$ order, it reads [5]

$$\mathcal{P}_2(\delta) := \prod_{j=1}^{M} \mathrm{e}^{-\mathrm{i}H_j\delta/2} \prod_{j=M}^{1} \mathrm{e}^{-\mathrm{i}H_j\delta/2}, \tag{35}$$

$$\mathcal{P}_{2k}(\delta) := \mathcal{P}_{2k-2}(a_k\delta)^2\, \mathcal{P}_{2k-2}((1-4a_k)\delta)\, \mathcal{P}_{2k-2}(a_k\delta)^2\,, \tag{36}$$

where the coefficients are given by $a_k := 1/\left(4 - 4^{1/(2k-1)}\right)$. The product limits indicate in which order the product is to be taken. The terms in the product run from right to left, as gates in a circuit would be applied, so that $\prod_{j=1}^{L} A_j = A_L \cdots A_1$.

Error Analysis of Higher-Order Formulae

We need an expression for the error $\mathcal{R}_p(T,\delta)$ arising from approximating the exact evolution $U(T)$ by a pth order product formula $\mathcal{P}_p(\delta)$ repeated T/δ times. As a first step, we bring the latter into the form:

$$\mathcal{P}_p(\delta) := \prod_{j=1}^{S} \mathcal{P}_{p,j}(\delta) = \mathcal{P}_{p,S}(\delta)\dots\mathcal{P}_{p,2}(\delta)\,\mathcal{P}_{p,1}(\delta)\,, \tag{37}$$

$$\mathcal{P}_{p,j}(\delta) := \prod_{i=1}^{M} U_{ij}(\delta) \quad \text{where} \quad U_{ij}(\delta) := \mathrm{e}^{-\mathrm{i}\delta b_{ji} H_i}. \tag{38}$$

As before, M denotes the number of non-commuting *layers* of interactions in the local Hamiltonian. $S = S_p$ is the number of *stages*; the number of $\mathcal{P}_{p,j}(\delta)$ in a pth order decomposition from Supplementary Equation (35) or Supplementary Equation (36). Here we note that we count a single stage as either $\prod_{i=1}^{M} U_{ij}(\delta)$ or $\prod_{i=M}^{1} U_{ij}(\delta)$, so that a second order formula is composed of 2 stages.

Lemma 12. For a pth-order decomposition with $p=1$ or $p=2k$, $k\geq 1$, we have $\sum_{j=1}^{S} b_{ji}(p) = 1$ for all $i = 1,\dots,M$. Furthermore, the Trotter coefficients b_{ji} satisfy

$$\max_{ij}\{|b_{ji}|\} \leq B_p \leq \begin{cases} 1 & p=1 \\ \frac{1}{2}\left(\frac{2}{3}\right)^{k-1} & p=2k,\ k\geq 1 \end{cases} \tag{39}$$

where

$$B_p := \begin{cases} 1 & p=1 \\ \frac{1}{2} & p=2 \\ \frac{1}{2}\prod_{i=2}^{k}(1-4a_i) & p=2k,\ k\geq 2. \end{cases} \tag{40}$$

Proof. The first claim is obviously true for the first order formula in Supplementary Equation (33). For higher orders, by [5, Th. 3] and Supplementary Equation (33), we have that the first derivative

$$\frac{\partial}{\partial x}\mathcal{P}_p(x)\bigg|_{x=0} = -\mathrm{i}\sum_{i=1}^{M} H_i. \tag{41}$$

Similarly, from Supplementary Equations (35) and (36), we have that

$$\frac{\partial}{\partial x}\mathcal{P}_p(x)\bigg|_{x=0} = \frac{\partial}{\partial x}\prod_{j=1}^{S}\prod_{i=1}^{M} U_{ij}(x)\bigg|_{x=0} = -\mathrm{i}\sum_{j=1}^{S}\sum_{i=1}^{M} b_{ji}H_i. \tag{42}$$

Equating both expressions for the first derivative of $\mathcal{P}_p(x)$ at $x=0$ and realising that they have to hold for any H_i yields the claim.

The second claim is again obviously true for a first order expansion, and follows immediately from Supplementary Equation (35) for $p = 2$. Expanding Supplementary Equation (36) for $\mathcal{P}_{2k}(\delta)$ all the way down to a product of $\mathcal{P}_2$ terms, the argument of each of the resulting factors will be a product of $k-1$ terms of $a_{k'}$ or $1-4a_{k'}$ for $k' \leq k$. We further note that for $k \geq 2$, $|a_k| \leq |1-4a_k|$, as well as $|a_k| \leq 1/2$ and $|1-4a_k| \leq 2/3$, which can be shown easily. The b_{ji} can thus be upper-bounded by B_p, which in turn is upper-bounded by $(1/2)(2/3)^{k-1}$ – where the final factor of $(1/2)$ is obtained from the definition of $\mathcal{P}_2$.

Since we are working with a fixed product formula order p for the remainder of this section, we will drop the order subscript in the following and write $\mathcal{P}_p = \mathcal{P}$, $\mathcal{R}_p = \mathcal{R}$ for simplicity. Assuming $\|H_i\| \leq \Lambda$ for all $i = 1, \ldots, M$, and setting the error

$$\epsilon_p(T,\delta) := \|\mathcal{R}(T,\delta)\| = \|U(T) - \mathcal{P}(\delta)^{T/\delta}\|, \tag{43}$$

we can derive an expression for the pth order error term. First, note that approximation errors in circuits accumulate at most linearly in Supplementary Equation (43). Thus it suffices to analyse a single δ step of the approximation, i.e. $U(\delta) = \mathcal{P}(\delta) + \mathcal{R}(\delta,\delta)$. Then

$$\epsilon_p(\delta) := \epsilon_p(\delta,\delta) = \|U(\delta) - \mathcal{P}(\delta)\| \tag{44}$$

so that

$$\epsilon_p(T,\delta) \leq \frac{T}{\delta}\epsilon_p(\delta). \tag{45}$$

We will denote $\epsilon_p(\delta)$ simply by ϵ in the following.

To obtain a bound on $\mathcal{P}(\delta)$, we apply the variation of constants formula with the condition that $\mathcal{P}(0) = I$, which always holds. As in [5, sec. 3.2], for $\delta \geq 0$, we obtain

$$\mathcal{P}(\delta) = U(\delta) + \mathcal{R}(\delta) = \mathrm{e}^{-\mathrm{i}\delta H} + \int_0^\delta \mathrm{e}^{-\mathrm{i}(\delta-\tau)H} R(\tau)\, d\tau \tag{46}$$

where the integrand $R(\tau)$ is defined as

$$R(\tau) := \frac{d}{d\tau}\mathcal{P}(\tau) - (-\mathrm{i}H)\mathcal{P}(\tau). \tag{47}$$

Now, if $\mathcal{P}(\delta)$ is accurate up to p^{th} order – meaning that $\mathcal{R}(\delta) = \mathrm{O}\left(\delta^{p+1}\right)$ – it holds that the integrand $R(\delta) = \mathrm{O}(\delta^p)$. This allows us to restrict its partial derivatives, as the following shows.

Lemma 13. For a product formula accurate up to p^{th} order – i.e. for which $R(\delta) = \mathrm{O}(\delta^p)$ – the partial derivatives $\partial_\tau^j R(0) = 0$ for all $0 \leq j \leq p-1$.

Proof. We note that $R(\delta)$ is analytic, which means that we can expand it as a Taylor series $R(\delta) = \sum_{j=0}^\infty a_j \delta^i$. We proceed by induction. If $a_0 \neq 0$, then clearly $R(0) \neq 0$, which

contradicts the assumption that $R(\delta)$ is accurate up to p^{th} order. Now assume for induction that $\forall j < j' < p-1 : a_j = 0$ and $a_{j'} \neq 0$. Then

$$\frac{R(\delta)}{T^{j'}} = a_{j'} + \sum_{i=1}^{\infty} a_{i+j'} T^i \xrightarrow{\delta \to 0} a_{j'} \neq 0, \tag{48}$$

which again contradicts that $R(0) = \mathrm{O}(\delta^p)$. The claim follows.

Performing a Taylor expansion of $R(\tau)$ around $\tau = 0$, the error bound ϵ given in Supplementary Equation (44) simplifies to

$$\epsilon = \left\| \int_0^\delta \mathrm{e}^{-\mathrm{i}(\delta-\tau)H} R(\tau)\, \mathrm{d}\tau \right\| \leq \int_0^\delta \|R(\tau)\| \mathrm{d}\tau \tag{49}$$

$$= \int_0^\delta \left(\|R(0)\| + \|R'(0)\| \tau + \ldots + \|R^{(p-1)}(0)\| \frac{\tau^{p-1}}{(p-1)!} + \|S(\tau, 0)\| \right) \mathrm{d}\tau, \tag{50}$$

Further by Supplementary Lemma 13 all but the pth or higher remainder terms $S(\tau, 0)$ equal zero, so

$$\epsilon \leq \int_0^\delta \|S(\tau, 0)\| \mathrm{d}\tau = p \int_0^\delta \int_0^1 (1-x)^{p-1} \|R^{(p)}(x\tau)\| \frac{\tau^p}{p!} \mathrm{d}x \mathrm{d}\tau, \tag{51}$$

where we used the integral representation for the Taylor remainder $S(\tau, 0)$.

Motivated by this, we look for a simple expression for the pth derivative of the integrand $R(\tau)$, which capture this in the following technical lemma.

Lemma 14. For a product formula accurate to p^{th} order, having $S = S_p$ stages for M non-commuting Hamiltonian layers with the upper-bound $\|H_i\| \leq \Lambda$, the error term $R(\tau)$ satisfies

$$\left\| \frac{\partial^p}{\partial \tau^p} R(\tau) \right\| \leq (SM)^{p+1} \Lambda^{p+1} \begin{cases} 2 & p = 1 \\ \frac{1}{2^p} \left(\frac{2}{3}\right)^{(p+1)(p/2-1)} & p = 2k \text{ for } k \geq 1. \end{cases} \tag{52}$$

Proof. We first express $\mathcal{P}(\tau)$ from Supplementary Equations (37) and (38) with a joint index set $\Sigma = [S] \times [M]$ as

$$\mathcal{P}(\tau) = \prod_{j=1}^{S} \prod_{i=1}^{M} U_{ij}(\tau) = \prod_{I \in \Sigma} U_I(\tau). \tag{53}$$

Then the $(p+1)^{\text{th}}$ derivative of this with respect to τ is

$$\mathcal{P}^{(p+1)}(\tau) = \sum_{\alpha\colon |\alpha| = p+1} \binom{p+1}{\alpha} \prod_I U_I^{(\alpha_I)}(\tau) \tag{54}$$

where α is a multiindex on Σ, and $|\alpha| = \sum_{I\in\Sigma} \alpha_I$. Following standard convention, the multinomial coefficient for a multiindex is defined as

$$\binom{p+1}{\alpha} = \frac{(p+1)!}{\alpha!} = \frac{(p+1)!}{\prod_{I\in\Sigma} \alpha_I!}. \tag{55}$$

We can similarly express H with the same index set σ, and as a derivative of U via

$$H = \sum_{i=1}^{S} H_i = \sum_{j=1}^{S}\sum_{i=1}^{M} b_{ji} H_i = \mathrm{i}\sum_{j=1}^{S}\sum_{i=1}^{M} U_{ij}^{(1)}(0) = \mathrm{i}\sum_{I\in\Sigma} U_I^{(1)}(0) \tag{56}$$

where we used the fact that $\sum_{j=1}^{S} b_{ji} = 1$ by Supplementary Lemma 12, and the exponential expression of U_I from Supplementary Equation (38).

Now we can combine Supplementary Equations (54) and (56) as in Supplementary Equation (51) to obtain the pth derivative of the integrand $R(\tau)$:

$$R^{(p)}(\tau) = \sum_{\alpha:\,|\alpha|=p+1} \binom{p+1}{\alpha} \prod_I U_I^{(\alpha_I)}(\tau) - \sum_I U_I^{(1)}(0) \sum_{\beta:\,|\beta|=p} \binom{p}{\beta} \prod_I U_I^{(\beta_I)}(\tau). \tag{57}$$

Noting that $\|U_I^{(\beta_I)}(\tau)\| = \|U_I^{(\beta_I)}(0)\|$, and further $U_I^{(x)}(0)\,U_I^{(y)}(0) = U_I^{(x+y)}(0)$, we have

$$\sum_J \left\|U_J^{(1)}(0)\right\| \sum_{\beta:\,|\beta|=p} \binom{p}{\beta} \prod_I \left\|U_I^{(\beta_I)}(0)\right\| = \sum_{\beta:\,|\beta|=p+1} \binom{p+1}{\beta} \prod_I \left\|U_I^{(\beta_I)}(0)\right\|. \tag{58}$$

We can therefore bound the norm of $R^{(p)}$ as follows:

$$\left\|R^{(p)}(\tau)\right\| \le \sum_{\alpha:\,|\alpha|=p+1} \binom{p+1}{\alpha} \prod_I \left\|U_I^{(\alpha_I)}(0)\right\| \tag{59}$$

$$+ \sum_I \left\|U_I^{(1)}(0)\right\| \sum_{\beta:\,|\beta|=p} \binom{p}{\beta} \prod_I \left\|U_I^{(\beta_I)}(0)\right\| \tag{60}$$

$$= 2 \sum_{\alpha:\,|\alpha|=p+1} \binom{p+1}{\alpha} \prod_I \left\|U_I^{(\alpha_I)}(0)\right\| \tag{61}$$

$$= 2 \sum_{\alpha:\,|\alpha|=p+1} \binom{p+1}{\alpha} \prod_{j=1}^{S}\prod_{i=1}^{M} |b_{ji}|^{\alpha_{ij}} \|H_i\|^{\alpha_{ij}}. \tag{62}$$

By Supplementary Lemma 12, we know that $|b_{ji}| = 1$ when $p = 1$ and $|b_{ji}| \le (2/3)^{p/2-1}/2$ for all j, i when $p = 2k$ for $k \ge 1$. Hence for $p = 1$

$$\left\|R^{(1)}(\tau)\right\| \le 2\,(SM)^2\,\Lambda^2, \tag{63}$$

and for $p = 2k$ for $k \ge 1$

$$\left\|R^{(p)}(\tau)\right\| \le 2 \sum_{\alpha:\,|\alpha|=p+1} \binom{p+1}{\alpha} \left[\left(\frac{2}{3}\right)^{p/2-1} \frac{\Lambda}{2}\right]^{|\alpha|} =: C_p(S,M) \left(\frac{2}{3}\right)^{(p+1)(p/2-1)} \frac{\Lambda^{p+1}}{2^p}, \tag{64}$$

where $C_p(S, M)$ is the sum of the multinomial coefficients of length $p \in \mathbb{N}$; a simple expression can be obtained by reversing the multinomial theorem, since

$$\sum_{\alpha:\, |\alpha|+p+1} \binom{p+1}{\alpha} = \Big(\underbrace{1+1+\ldots+1}_{|\Sigma|\text{ terms}}\Big)^{p+1} = |\Sigma|^{p+1} = (SM)^{p+1}\,. \tag{65}$$

To obtain the final error bounds, we combine Supplementary Lemma 14 with the integral representation in Supplementary Equation (51).

Theorem 15 (Trotter Error). For a pth order product formula $\mathcal{P}_p$ for $p = 1$ or $p = 2k$, $k \geq 1$, with the same setup as in Supplementary Lemma 14, a bound on the approximation error for the exact evolution $U(T)$ with T/δ rounds of the product formula $\mathcal{P}_p(\delta)$ is given by

$$\epsilon_p(T, \delta) \leq \frac{T}{\delta}\delta^{p+1}M^{p+1}\Lambda^{p+1} \times \begin{cases} 1 & p = 1 \\ \dfrac{2}{(p+1)!}\left(\dfrac{10}{3}\right)^{(p+1)(p/2-1)} & p = 2k,\ k \geq 1. \end{cases} \tag{66}$$

Proof. We can use the bound on $R^{(p)}$ derived in Supplementary Lemma 14 and perform the integration over τ and x in Supplementary Equation (51), to obtain

$$\epsilon \leq \|R^{(p)}\| \int_0^\delta p \int_0^1 (1-x)^{p-1}\frac{\tau^p}{p!}\mathrm{d}x\mathrm{d}\tau = \frac{\delta^{p+1}}{(p+1)!}\|R^{(p)}\|. \tag{67}$$

By Supplementary Lemma 13, for Trotter formulae of order $p = 1$ we have precisely one stage, i.e. $S = 1$, and $b_{ji} = 1$ for all i, j. This, together with Supplementary Lemma 14 and Supplementary Equation (44), yields the first bound.

The number of stages in higher order formulae can be upper-bounded by Supplementary Equations (35) and (36), giving $S_p \leq 2 \times 5^{p/2-1}$. Together with Supplementary Lemma 14 and Supplementary Equation (44), this yields the second bound.

We remark that tighter bounds than the ones in Supplementary Theorem 15 are achievable for any given product formula, where the form of its coefficients b_{ji} are explicitly available and not merely bounded as in Supplementary Lemma 12. Summing up these stage times exactly is therefore an immediate way to obtain an improved error bound. Furthermore, the triangle inequality on $\|R^{(p)}(\tau)\|$ in the proof of Supplementary Lemma 14 is a crude overestimate: it looses information about (i). terms that could cancel between the two multi-index sums, and (ii.) any commutation relations between the individual Trotter stages.

In the following subsection, we will provide a tighter error analysis, featuring more optimal but less clean analytical expressions which we can nonetheless evaluate efficiently numerically.

Explicit Summation of Trotter Stage Coefficients

For the recursive Suzuki-Trotter formula in Supplementary Equation (36) we can immediately improve the error bound by summing the stage coefficients b_{ij} up exactly, instead of bounding them as in Supplementary Lemma 12.

Corollary 16 (Trotter Error). For the recursive product formula in Supplementary Equation (36) and $p = 2k$ for $k \geq 1$,

$$\epsilon_p(T,\delta) \leq \frac{2T\delta^p M^{p+1}\Lambda^{p+1}}{(p+1)!} H_p^{p+1} \quad \text{where} \quad H_p := \prod_{i=1}^{p/2-1} \frac{4+4^{1/(2i+1)}}{\left|4-4^{1/(2i+1)}\right|}. \tag{68}$$

Proof. This follows from explicitly summing up the magnitudes of all the b_{ji}'s obtained by solving the recursive definition of the product formula, which can easily be verified to satisfy $\sum_{ij} |b_{ij}(p)| = MH_p$. Then from Supplementary Lemma 14,

$$\left\|R^{(p)}(\tau)\right\| \leq 2\Lambda^{p+1} \sum_{\alpha:\,|\alpha|=p+1} \binom{p+1}{\alpha} \prod_{j=1}^{S}\prod_{i=1}^{M} \left|b_{ji}^{\alpha_{ij}}\right| = 2\Lambda^{p+1}\left(\sum_{j=1}^{S}\sum_{i=1}^{M}|b_{ji}|\right)^{p+1}, \tag{69}$$

and the claim follows as before.

For later reference, we note that it is straightforward to generalise the error bound in Supplementary Corollary 16 for the case of a *higher* derivative $R^{(q)}$, $q \geq p$, but still for a pth order formula: the bound simply reads

$$\epsilon_{p,q}(T,\delta) \leq \frac{2T\delta^q M^{q+1}\Lambda^{q+1}}{(q+1)!} H_p^{q+1}. \tag{70}$$

Commutator Bounds

Our analysis thus far has completely neglected the underlying structure of the Hamiltonian. In this subsection we establish commutator bounds which are easily applicable to D-dimensional lattice Hamiltonians.

We begin with the following technical lemmas.

Lemma 17. For a product formula accurate to p^{th} order, having $S = S_p$ stages for M non-commuting Hamiltonian layers with the upper-bound $\|H_i\| = \Lambda_I$, the error term $R(\tau)$ satisfies

$$\left\|\frac{\partial^p}{\partial\tau^p}R(\tau)\right\| \leq \sum_J \sum_{\beta:\,|\beta|=p} \binom{p}{\beta} \sum_{I=J+1}^{SM} (B_p\Lambda)^{p-\beta_I} \left\|\left[U_J^{(1)}(0)\;, U_I^{(\beta_I)}(\tau)\right]\right\|. \tag{71}$$

Proof. As shown in Supplementary Lemma 14,

$$R^{(p)}(\tau) = \sum_{\alpha:\,|\alpha|=p+1} \binom{p+1}{\alpha} \prod_I U_I^{(\alpha_I)}(\tau) - \sum_J U_J^{(1)}(0) \sum_{\beta:\,|\beta|=p} \binom{p}{\beta} \prod_I U_I^{(\beta_I)}(\tau). \tag{72}$$

We begin by commuting every $U_J^{(1)}(0)$ past $\prod_{I=J+1}^{SM} U_I^{(\beta_I)}(\tau)$. Consider this for some fixed J in the sum of over J. That is consider rewriting a particular summand from the second term above to obtain

$$U_J^{(1)}(0) \sum_{\beta:\,|\beta|=p} \binom{p}{\beta} \prod_I U_I^{(\beta_I)}(\tau) \tag{73}$$

$$= \sum_{\beta:\,|\beta|=p} \binom{p}{\beta} U_J^{(1)}(0) \left(U_{sm}^{(\beta_{sm})}(\tau) \ldots U_{J+1}^{(\beta_{J+1})}(\tau) \right) \left(U_J^{(\beta_J)}(\tau) \ldots U_1^{(\beta_1)}(\tau) \right) \tag{74}$$

$$= \sum_{\beta:\,|\beta|=p} \binom{p}{\beta} \left(U_{sm}^{(\beta_{sm})}(\tau) \ldots U_{J+1}^{(\beta_{J+1})}(\tau) \right) \left(U_J^{(\beta_J+1)}(\tau) \ldots U_1^{(\beta_1)}(\tau) \right) \tag{75}$$

$$+ \sum_{\beta:\,|\beta|=p} \binom{p}{\beta} \left[U_J^{(1)}(0) \ , \prod_{I=J+1}^{SM} U_I^{(\beta_I)}(\tau) \right] \prod_{I=1}^{J} U_I^{(\beta_I)}(\tau). \tag{76}$$

Now, by inserting this into the full expression for $R^{(p)}(\tau)$, we obtain

$$R^{(p)}(\tau) = \sum_{\alpha:\,|\alpha|=p+1} \binom{p+1}{\alpha} \prod_I U_I^{(\alpha_I)}(\tau) - \sum_{\beta:\,|\beta|=p+1} \binom{p+1}{\beta} \prod_I U_I^{(\beta_I)}(\tau) \tag{77}$$

$$- \sum_J \sum_{\beta:\,|\beta|=p} \binom{p}{\beta} \left[U_J^{(1)}(0) \ , \prod_{I=J+1}^{SM} U_I^{(\beta_I)}(\tau) \right] \prod_{I=1}^{J} U_I^{(\beta_I)}(\tau) \tag{78}$$

$$= - \sum_J \sum_{\beta:\,|\beta|=p} \binom{p}{\beta} \left[U_J^{(1)}(0) \ , \prod_{I=J+1}^{SM} U_I^{(\beta_I)}(\tau) \right] \prod_{I=1}^{J} U_I^{(\beta_I)}(\tau) \tag{79}$$

$$= - \sum_J \sum_{\beta:\,|\beta|=p} \binom{p}{\beta} \sum_{I=J+1}^{SM} \prod_{K=I+1}^{SM} U_K^{(\beta_K)} \left[U_J^{(1)}(0) \ , U_I^{(\beta_I)}(\tau) \right] \prod_{K=1}^{I-1} U_K^{(\beta_K)}. \tag{80}$$

Taking the norm of this expression gives

$$\left\| \frac{\partial^p}{\partial \tau^p} R(\tau) \right\| \leq \sum_J \sum_{\beta:\,|\beta|=p} \binom{p}{\beta} \sum_{I=J+1}^{SM} (B_p \Lambda)^{\sum_{K=I+1}^{SM} \beta_K} \left\| \left[U_J^{(1)}(0) \ , U_I^{(\beta_I)}(\tau) \right] \right\| (B_p \Lambda)^{\sum_{K=1}^{I-1} \beta_K} \tag{81}$$

$$= \sum_J \sum_{\beta:\,|\beta|=p} \binom{p}{\beta} \sum_{I=J+1}^{SM} (B_p \Lambda)^{p-\beta_I} \left\| \left[U_J^{(1)}(0) \ , U_I^{(\beta_I)}(\tau) \right] \right\|. \tag{82}$$

This completes the proof.

Lemma 18. If every pair of Hamiltonians can be written as $H_I = \sum_{i=1}^N h_i^I$ and $H_J = \sum_{i=1}^N h_i^J$, where for any i we have $\|h_i^I\| = \|h_i^J\| = 1$ and for any fixed term h^J there are at most n terms in H_I which do not commute with that specific term, then

$$\left\| \left[U_J^{(1)}(0) \ , U_I^{(\beta_I)}(0) \right] \right\| \leq 2n\beta_I N^{\beta_I} B_p^{\beta_I+1}. \tag{83}$$

Proof. First note that

$$U_J^{(1)}(0) = -\mathrm{i}b_J H_J = -\mathrm{i}b_J \sum_{i=1}^{N} h_i^J \tag{84}$$

and

$$U_I^{(\beta_I)}(0) = (-\mathrm{i}b_I H_I)^{\beta_I} = (-\mathrm{i}b_I)^{\beta_I} \left(\sum_{i=1}^{N} h_i^I\right)^{\beta_I}. \tag{85}$$

Consider a fixed term in $U_J^{(1)}(0)$ such as $-\mathrm{i}b_J h^J$, where we have dropped the subscript i. As there are N of these, we can bound the norm of the commutator as follows

$$\left\| \left[U_J^{(1)}(0), U_I^{(\beta_I)}(0)\right] \right\| \leq N B_p \left\| \left[h^J, U_I^{(\beta_I)}(0)\right] \right\|. \tag{86}$$

Where we have also used the triangle inequality and the fact that $b_J \leq B_p$.

Now consider fully expanding the $U_I^{(\beta_I)}$ so that it is a sum of N^{β_I} norm-1 Hamiltonians with coefficients upper-bounded by $(B_p)^{\beta_I}$. As only n of the N normalised Hamiltonians do not commute with h^J, the number of Hamiltonians in the expanded $U_I^{(\beta_I)}$ which do not commute with h^J can be upper-bounded by $n\beta_I N^{\beta_I - 1}$. Here we have assumed that if any of the n non-commuting terms appear at any point in the expansion (the n), then that term will not commute with h^J regardless of whatever other terms appear (the N^{β_I-1}). We can over-count by repeating this for each term expanded (the β_I). This gives

$$\left\| \left[U_J^{(1)}(0), U_I^{(\beta_I)}(0)\right] \right\| \leq 2n\beta_I N^{\beta_I} B_p^{\beta_I+1}. \tag{87}$$

The extra factor of 2 comes from bounding the commutators of the norm 1 Hamiltonians via triangle inequality.

Lemma 19. If every pair of Hamiltonians can be written as $H_I = \sum_{i=1}^{N} h_i^I$ and $H_J = \sum_{i=1}^{N} h_i^J$, where all $\|h_i^I\| = \|h_i^J\| = 1$, and if additionally for any fixed term h^J there are at most n terms h^I which do not commute with h^J, then

$$\left\| \frac{\partial^p}{\partial \tau^p} R(\tau) \right\| \leq npB_p^{p+1}\Lambda^{p-1}N\left((SM-1)+\frac{N}{\Lambda}\right)^{p-1}\left((SM)^2-(SM)\right) \tag{88}$$

$$+ n\tau B_p^{p+2}\Lambda^p N \mathrm{e}^{\tau N B_p}\left((SM)^{p+2}-(SM)^{p+1}\right). \tag{89}$$

Proof. We must obtain a simplified form for the bounded commutator appearing in Supplementary Lemma 17. We can sequentially expand this commutator and use the triangle inequality to write it as

$$\left\| \left[U_J^{(1)}(0), U_I^{(\beta_I)}(\tau)\right] \right\| = \left\| \left[U_J^{(1)}(0), U_I^{(\beta_I)}(0)\,\mathrm{e}^{\mathrm{i}\tau b_I H_I}\right] \right\| \tag{90}$$

$$\leq \left\| \left[U_J^{(1)}(0), U_I^{(\beta_I)}(0)\right] \right\| + B_p^{\beta_I}\Lambda^{\beta_I} \left\| \left[U_J^{(1)}(0), \mathrm{e}^{\mathrm{i}\tau b_I H_I}\right] \right\|. \tag{91}$$

We can use Supplementary Lemma 18 to bound the first term. The commutator in the second term can be bounded as follows:

$$\left\|\left[U_J^{(1)}(0), e^{i\tau b_I H_I}\right]\right\| = \left\|\left[U_J^{(1)}(0), I + i\tau b_I H_I + \frac{1}{2!}(i\tau b_I H_I)^2 + \dots\right]\right\| \tag{92}$$

$$\leq \sum_{k=1}^{\infty} \frac{\tau^k}{k!}\left\|\left[U_J^{(1)}(0), U_I^{(k)}(0)\right]\right\| \tag{93}$$

$$\leq \sum_{k=1}^{\infty} \frac{2n\tau^k}{(k-1)!} N^k B_p^{k+1} \tag{94}$$

$$= 2n\tau N B_p^2 e^{N B_p \tau}. \tag{95}$$

Where we have used Supplementary Lemma 18 to bound the norm of the commutator of $U_J^{(1)}(0)$ and $U_I^{(k)}(0)$ by $2nkN^k B_p^{k+1}$ and simplified the resulting expression. The first term can be bounded directly with Supplementary Lemma 18, so we obtain

$$\left\|\left[U_J^{(1)}(0), U_I^{(\beta_I)}(\tau)\right]\right\| \leq 2n\beta_I N^{\beta_I} B_p^{\beta_I+1} + 2n\tau N \Lambda^{\beta_I} B_p^{2+\beta_I} e^{N B_p \tau}. \tag{96}$$

Now by using this to bound the result of Supplementary Lemma 17 we obtain

$$\left\|\frac{\partial^p}{\partial \tau^p} R(\tau)\right\| \leq \sum_J \sum_{\beta:\,|\beta|=p} \binom{p}{\beta} \sum_{I=J+1}^{SM} (B_p \Lambda)^{p-\beta_I} \left\|\left[U_J^{(1)}(0)\ , U_I^{(\beta_I)}(\tau)\right]\right\| \tag{97}$$

$$\leq \sum_J \sum_{\beta:\,|\beta|=p} \binom{p}{\beta} \sum_{I=J+1}^{SM} (B_p \Lambda)^{p-\beta_I} \left(2n\beta_I N^{\beta_I} B_p^{\beta_I+1} + 2n\tau N \Lambda^{\beta_I} B_p^{2+\beta_I} e^{N B_p \tau}\right) \tag{98}$$

$$= \sum_J \sum_{\beta:\,|\beta|=p} \binom{p}{\beta} \sum_{I=J+1}^{SM} \left(2n\beta_I \left(\frac{N}{\Lambda}\right)^{\beta_I} \Lambda^p B_p^{p+1} + 2n\tau N \Lambda^p B_p^{p+2} e^{N B_p \tau}\right). \tag{99}$$

To simplify this expression, we must simplify an expression of the form

$$\sum_{\beta:\,|\beta|=p} \binom{p}{\beta} \beta_I x^{\beta_I} \tag{100}$$

where in our case $x = N/\Lambda$. This can be done by rewriting this expression in terms of a derivative with respect to x and reversing the multinomial theorem, which gives

$$\sum_{\beta:\,|\beta|=p} \binom{p}{\beta} \beta_I x^{\beta_I} = x\frac{d}{dx} \sum_{\beta:\,|\beta|=p} \binom{p}{\beta} x^{\beta_I} \tag{101}$$

$$= x\frac{d}{dx}\Big(\underbrace{1 + \ldots + 1 + x}_{SM \text{ terms}}\Big)^p \tag{102}$$

$$= px\,(SM - 1 + x)^{p-1}. \tag{103}$$

Using this and performing the summation over J and I simplifies the expression for $\left\| R^{(p)}(\tau) \right\|$ to

$$\left\| \frac{\partial^p}{\partial \tau^p} R(\tau) \right\| \leq pnB_p^{p+1} \Lambda^{p-1} N \left(SM - 1 + \frac{N}{\Lambda} \right)^{p-1} \left((SM)^2 - (SM) \right) \tag{104}$$

$$+ \tau n B_p^{p+2} \Lambda^p N \left((SM)^{p+2} - (SM)^{p+1} \right) e^{\tau N B_p}. \tag{105}$$

Now we can use the preceding lemmas to establish a commutator bound for higher order Trotter formulae. Although it is cumbersome looking, it is easy to evaluate.

Theorem 20 (Commutator Error Bound). Let $H = \sum_{i=1}^{M} H_i$ with $\|H_i\| \leq \Lambda$ be a Hamiltonian with M mutually commuting layers $H_I = \sum_{i=1}^{N} h_i^I$. Assume that for any i, $\|h_i^I\| = \|h_i^J\| \leq 1$. Additionally, assume that for any fixed term h^J there exist at most n terms h^I which do not commute with h^J.

Then, for a pth order product formula $\mathcal{P}_p$ with $p = 1$ or $p = 2k$, $k \geq 1$ used to approximate the evolution operator under H, the approximation error for the exact evolution $U(T)$ with T/δ rounds of the product formula $\mathcal{P}_p(\delta)$ is bounded by

$$\epsilon_p(T, \delta) \leq C_1 \frac{T\delta^p}{(p+1)!} + C_2 \frac{T}{\delta} \int_0^\delta p \int_0^1 (1-x)^{p-1} \frac{x\tau^{p+1}}{p!} e^{x\tau N B_p} dx d\tau \tag{106}$$

with

$$C_1 = npB_p^{p+1} \Lambda^{p-1} N \left((SM - 1) + \frac{N}{\Lambda} \right)^{p-1} \left((SM)^2 - (SM) \right) \tag{107}$$

$$C_2 = nB_p^{p+2} \Lambda^p N \left((SM)^{p+2} - (SM)^{p+1} \right). \tag{108}$$

Proof. The error formula for a single Trotter step is given by Supplementary Equation (51) as

$$\epsilon_p(\delta) \leq p \int_0^\delta \int_0^1 (1-x)^{p-1} \|R^{(p)}(x\tau)\| \frac{\tau^p}{p!} dx d\tau. \tag{109}$$

Evaluating this using Supplementary Lemma 19 and then substituting the resultant expression in $\epsilon_p(T, \delta) \leq (T/\delta)\epsilon_p(\delta)$ gives the stated expression.

For later reference, we note that it is straightforward to generalise the error bound in Supplementary Theorem 20, by incorporating similar techniques to Supplementary Corollary 16 in order to sum up the $|b_{ij}|$ exactly, instead of simply bounding them by B_p. Additionally, we can also generalise to the case of a *higher* derivative $R^{(q)}$, $q \geq p$, but still for a pth order formula: with these two generalisations the bound simply reads

$$\epsilon_{p,q}(T, \delta) \leq C_1 \frac{T\delta^q}{(q+1)!} + C_2 \frac{T}{\delta} \int_0^\delta q \int_0^1 (1-x)^{q-1} \frac{x\tau^{q+1}}{q!} e^{x\tau N B_p} dx d\tau \tag{110}$$

with

$$C_1 = nqB_p^2\Lambda^{q-1}N\left(MH_p - B_p + B_p\left(\frac{N}{\Lambda}\right)\right)^{q-1}\left((S_pM)^2 - (S_pM)\right) \tag{111}$$

$$C_2 = nB_p^2\left(MH_p\Lambda\right)^q N\left((S_pM)^2 - (S_pM)\right). \tag{112}$$

A Taylor Bound on the Taylor Bound

Another method to obtain a tighter bound on a Taylor expansion as used on $R(\tau)$ in Supplementary Equation (46) and which can be used together with the more sophisticated commutator-based error bound from Supplementary Theorem 20 derived in the last section, can be obtained by performing a Taylor expansion of the remainder term, and in turn bounding *its* Taylor remainder by some other method [11, Rem. 4].

We first establish the following technical lemma:

Lemma 21 (Taylor Error Bound). Let the setup be as in Supplementary Lemma 14, and let $q > p$. The error term ϵ from Supplementary Equation (43) satisfies

$$\epsilon_p(\delta) \le \sum_{l=p}^{q} \frac{\delta^{l+1}}{(l+1)!}\|R^{(l)}(0)\| + \epsilon_{p,q+1}(\delta) \tag{113}$$

where

$$R(0)^{(l)} = \sum_{\alpha\colon |\alpha|=p+1}\binom{p+1}{\alpha}F(\alpha) + \mathrm{i}H\sum_{\beta\colon |\beta|=p}\binom{p}{\beta}F(\beta), \tag{114}$$

with $H = \sum_{i=1}^M H_i$, and

$$F(\alpha) := \prod_{j=1}^{S}\prod_{i=1}^{M}(-\mathrm{i}b_{ji}H_i)^{\alpha(i,j)}. \tag{115}$$

Proof. The expression for ϵ stems from Taylor-expanding Supplementary Equation (51) to order q instead of p, and integrating over τ. The last term $\epsilon_{q+1,p}$ is then simply the overall remainder S, as before; and we can use Supplementary Equation (70) to obtain a bound on it. The bound on $\|R(0)^{(l)}\|$ is an immediate consequence of Supplementary Equation (57), where we set $\tau = 0$.

This allows us to calculate a numerical bound on $\|R(0)^{(l)}\|$, by bounding $\|H_i\| \le \Lambda$ and allowing terms within the two sums over α and β to cancel. The benefit of this approach is that it is generically applicable to any given Trotter formula, and only depends on the non-commuting layers of H.

We can therefore derive the following bounds:

	$f(p,M,l)$ for $l=\cdot$						
	M	l=p	l=p+1	l=p+2	l=p+3	l=p+4	l=p+5
p=1	2	2	6	14	30	62	126
	3	6	26	90	290	906	2786
	4	12	68	312	1340	5592	22988
	5	20	140	800	4292	22400	115220
p=2	2	3	9	22.75	50	108.344	225.531
	3	13	57	213.25	711.25	2309.47	7283.06
	4	34	198	980.5	4377.5	18926.6	79758
	5	70	510	3141.5	17555	94765.3	499391
p=4	2	4.89745	19.5277	79.5305	442.266	2312.73	11208.3
	3	43.6604	277.994	1880.62	16924.7		
	4	194.476	1719.69	16226.8			
	5	610.187	6926.95	83775.9			

Supplementary Table 1: Trotter error coefficients $f(p,M,l)$ from Supplementary Corollary 22; values rounded to the precision shown.

Corollary 22 (Taylor Error Bound). Let $H=\sum_{i=1}^{M} H_i$ with $\|H_i\|\leq\Lambda$ for all i. Then for ϵ from Supplementary Equation (51), and for a pth Trotter formula, we have

$$\epsilon_p(\delta)\leq\sum_{l=p}^{q}\frac{\delta^{l+1}\Lambda^{l+1}}{(l+1)!}f(p,M,l)+\epsilon_{p,q+1}(\delta), \tag{116}$$

where

$$f(p,M,l)=\left\|\sum_{\alpha\colon|\alpha|=p+1}\binom{p+1}{\alpha}v(\alpha)+\mathrm{i}\sum_{j=1}^{M}|j\rangle\otimes\sum_{\beta\colon|\beta|=p}\binom{p}{\beta}v(\beta)\right\|_1, \tag{117}$$

and

$$v(\alpha):=\bigotimes_{j=1}^{S}\bigotimes_{i=1}^{M}(-\mathrm{i}b_{ji}\,|i\rangle)^{\otimes\alpha_{(i,j)}}. \tag{118}$$

for a basis $\{|1\rangle,\ldots,|M\rangle\}$ of $\mathbb{C}^M$.

Proof. Follows immediately from Supplementary Lemma 21.

A selection of the series coefficients $f(p,M,l)$ can be found in Supplementary Table 1. Supplementary Corollary 22 can then be applied in conjunction with e.g. the commutator error bound given in Supplementary Theorem 20 for the remaining term $\epsilon_{q+1}(\delta,\delta)$.

Spectral Norm of Fermionic Hopping Terms

Let $a^\dagger$ and a be the standard fermionic creation and annihilation operators.

Theorem 23. Let $\Omega = \{ij\}$ be a set of pairs of indices such that no two pairs share an index. Define:

$$H_\Omega = \sum_{ij\in\Omega} h_{ij} \ , \ \ h_{ij} = a_i^\dagger a_j + a_j^\dagger a_i \tag{119}$$

Given a normalized fermionic state $|\psi\rangle$ such that $N|\psi\rangle = n|\psi\rangle$:

$$|\langle\psi| H_\Omega |\psi\rangle| \leq \min(n, M-n, |\Omega|) \tag{120}$$

Where M is the number of fermionic modes. This bound is tight.

Proof. Consider that h_{ij} has eigenvalues in $\{-1,0,1\}$, since $h_{ij}^2 = (N_i - N_j)^2$ which has eigenvalues $\{0,1\}$. Suppose there existed a normalised state $|\psi\rangle$ such that $N|\psi\rangle = n|\psi\rangle$ and $H_\Omega|\psi\rangle = \lambda|\psi\rangle$ where $|\lambda| > \min(n, M-n, |\Omega|)$. Since H_Y, N and all h_{ij} are all mutually commuting, we may choose $|\psi\rangle$ to be an eigenstate of all h_{ij} wlog (by convexity). Then it must be the case that $h_{ij}^2|\psi\rangle = |\psi\rangle$ for at least $|\lambda|$ pairs ij, which implies that in the Fock basis $|\psi\rangle = a|0_i, 1_j, ..\rangle + b|1_i, 0_j, ..\rangle$. Therefore for at least $|\lambda|$ pairs $ij \in \Omega$ we have $\langle\psi|(N_i + N_j)|\psi\rangle = 1$. So $\langle\psi|N|\psi\rangle \geq |\lambda|$ and $M - \langle\psi|N|\psi\rangle \geq |\lambda|$. If $\min(n, M-n, |\Omega|) = n$ then $\langle\psi|N|\psi\rangle > n$ which is a contradiction. If $\min(n, M-n, |\Omega|) = M-n$ then $M - \langle\psi|N|\psi\rangle > M-n$ which is a contradiction. If $\min(n, M-n, |\Omega|) = |\Omega|$ then $|\lambda| > |\Omega|$ which is a contradiction. This proves the bound.

Now we need only show the bound is tight. Consider the following state:

$$\left|\phi_{ij}^\pm\right\rangle = (a_i^\dagger \pm a_j^\dagger)\Gamma_s|0\rangle \tag{121}$$

With Γ_s composed of creation and annihilation operators which do not include i or j. This state is an eigenstate of $h_{ij} = a_i^\dagger a_j + a_j^\dagger a_i$:

$$h_{ij}\left|\phi_{ij}^\pm\right\rangle = \pm\left|\phi_{ij}^\pm\right\rangle \tag{122}$$

Observe:

$$h_{ij}\left|\phi_{ij}^\pm\right\rangle = h_{ij}(a_i^\dagger \pm a_j^\dagger)\Gamma_s|0\rangle \tag{123}$$
$$= (a_i^\dagger a_j a_i^\dagger + a_j^\dagger a_i a_i^\dagger \pm a_i^\dagger a_j a_j^\dagger \pm a_j^\dagger a_i a_j^\dagger)\Gamma_s|0\rangle \tag{124}$$
$$= (a_j^\dagger a_i a_i^\dagger \pm a_i^\dagger a_j a_j^\dagger)\Gamma_s|0\rangle \tag{125}$$
$$= ((-a_j^\dagger a_i^\dagger a_i + a_j^\dagger) \pm (-a_i^\dagger a_j^\dagger a_j + a_i^\dagger))\Gamma_s|0\rangle \tag{126}$$
$$= (a_j^\dagger \pm a_i^\dagger)\Gamma_s|0\rangle \tag{127}$$
$$h_{ij}\left|\phi_{ij}^\pm\right\rangle = \pm(a_i^\dagger \pm a_j^\dagger)\Gamma_s|0\rangle \tag{128}$$

Consider a set of pairs of indices $\omega \subseteq \Omega$. Choose an ordering on ω and define

$$\left|\phi_\omega^b\right\rangle = \prod_{ij\in\omega} (a_i^\dagger + (-1)^{b_{ij}} a_j^\dagger) \left|0\right\rangle \tag{129}$$

with b a bit-string indexed by ij. Note that $N\left|\phi_\omega^b\right\rangle = |\omega|\left|\phi_\omega^b\right\rangle$. We now argue that b can always be chosen such that:

$$H_\Omega \left|\phi_\omega^b\right\rangle = |\omega| \left|\phi_\omega^b\right\rangle . \tag{130}$$

Choose a pair $ij \in \omega$, the state $\left|\phi_\omega^b\right\rangle$ can be expressed as:

$$\left|\phi_\omega^b\right\rangle = (\delta_i a_i^\dagger + (-1)^{b_{ij}} \delta_j a_j^\dagger)\Gamma_s \left|0\right\rangle , \ \delta_i, \delta_j \in \{-1, 1\} \tag{131}$$

With Γ_s composed of creation and annihilation operators which do not include i or j. So

$$\left|\phi_\omega^b\right\rangle = \delta_i \left|\phi_{ij}^{\Delta_{ij}}\right\rangle \ \ , \Delta_{ij} = \delta_i\delta_j(-1)^{b_{ij}}. \tag{132}$$

Let us choose b_{ij} such that $\Delta_{ij} = 1$. Noting that Δ_{ij} is independent of Δ_{pq} when $pq \neq ij$ we can do the same for all other b_{pq}. This gives:

$$H_\Omega \left|\phi_\omega^b\right\rangle = \sum_{ij\in\omega} h_{ij}\delta_i \left|\phi_{ij}^+\right\rangle \tag{133}$$

$$H_\Omega \left|\phi_\omega^b\right\rangle = \sum_{ij\in\omega} \delta_i \left|\phi_{ij}^+\right\rangle \tag{134}$$

$$H_\Omega \left|\phi_\omega^b\right\rangle = \sum_{ij\in\omega} \left|\phi_\omega^b\right\rangle \tag{135}$$

$$H_\Omega \left|\phi_\omega^b\right\rangle = |\omega| \left|\phi_\omega^b\right\rangle \tag{136}$$

Note that $n = |\omega| < |\Omega|$ and $|\Omega| < M/2$ and so the bound is shown to be tight in the case where $\min(n, M-n, |Y|) = n$.

If we consider the case where $\min(n, M-n, |\Omega|) = |\Omega|$, then we may always choose ω such that it is composed of a set of pairs of indices such that no two pairs share an index, and such that $\Omega \subseteq \omega$. In this case, by a similar argument

$$H_\Omega \left|\phi_\omega^b\right\rangle = |\Omega| \left|\phi_\omega^b\right\rangle . \tag{137}$$

Finally, in the case where $\min(n, M-n, |\Omega|) = M-n$ one may choose the particle-hole symmetric state

$$\left|\tilde{\phi}_\omega^b\right\rangle = \prod_{ij\in\omega} (a_i + (-1)^{b_{ij}} a_j) \prod_{k=1}^{M} a_k^\dagger \left|0\right\rangle \tag{138}$$

and a similar argument follows by particle hole symmetry.

Simulating Fermi-Hubbard via Sub-Circuit Algorithms

Overview and Benchmarking of Analysis

In the following sections we primarily adopt the per-time error model and associated metric for costing circuits, Supplementary Definition 3. We first establish asymptotic bounds on the run-time $\mathcal{T}_{\text{cost}}$ of performing a time-dynamics simulation of a 2D spin Fermi-Hubbard Hamiltonian using a pth-order Trotter formula with $M = 5$ Trotter layers, for a target time T and target error ϵ_t. We perform this analysis for both the compact and VC encodings and the results are summarised in Supplementary Corollary 26.

We first want to compare using sub-circuit vs standard circuit decompositions in a per-time error model. We do this in conjunction with our Trotter bounds. To this end we establish the analytic bounds for the same simulation task, first using the standard conjugation method to generate evolution under higher weight interactions using only standard CNOT gates and single qubit rotations as opposed to a sub-circuit pulse sequence. We choose this method as it doesn't introduce any unfair and needless analytic error into the comparison. We decompose the Trotter steps into a standard gate set of CNOTs and single-qubit rotations which are gates of the form $e^{\pm i\pi/4ZZ}$ up to single qubit rotations. We cost this with the same metric using a per-time error model, but do not allow the comparison to contain any gates of the form $e^{\pm i\delta ZZ}$ as this would constitute a sub-circuit gate.

In this comparative analytic expression we still use our Trotter error bounds, so Supplementary Corollary 25 only serves to evaluate the impact of differing Trotter step synthesis methods on the asymptotic scaling of the run-time $\mathcal{T}_{\text{cost}}$ in a per-time error model.

Later in this section we perform a tighter numerical analysis of both our proposal and our standard circuit model comparison. In these numerics we compare our Trotter bounds to readily applicable bounds from the literature [4, Prop. F.4.]. We point out that these bounds do not exploit the underlying structure of the Hamiltonian or make use of the recent advances of [5], [6]. However these bounds contained all constants, were applicable to 2D lattices and could easily be evaluated for arbitrary p and allowed us to make use of Supplementary Theorem 23. We were able to compare our bounds to [5] for the case of a simple 1D lattice and establish that our bounds are preferable for medium system sizes, not in asymptotic limits of system size, as was our intention in reformulating bounds for NISQ applications.

After this comparison in the framework of a per-time error model, we numerically analyse the impact of sub-circuit techniques in the per-gate error model, calculating the circuit depth of sub-circuit Trotter simulations. This is shown in Supplementary Figures 16 and 18.

The Fermi-Hubbard Hamiltonian and Fermionic Encodings

We consider a Fermi-Hubbard model on a 2D lattice of $N = L \times L$ fermionic sites. There is hopping between nearest neighbours only and on-site interactions between fermions of

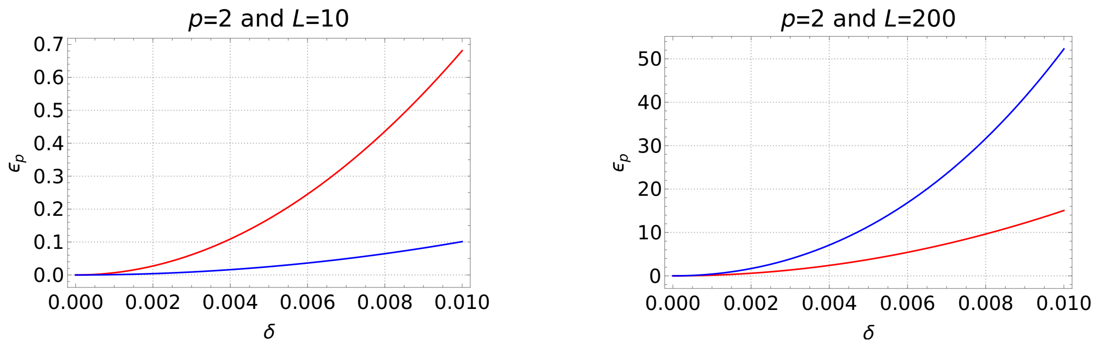

Supplementary Figure 6: A comparison of 2nd order Trotter bounds $\epsilon_p(\delta, L)$ for a 1D lattice Hamiltonian of length L split into even and odd layers of interactions $H = H_{\text{even}} + H_{\text{odd}}$. The bounds are our Supplementary Theorem 20 (blue) and the main result stated in [5] which we've evaluated from [5, Sec. B:eq. 57+58] (red). This illustrates why we have derived Trotter bounds which sacrifice scaling in system size in favour of bounds with smaller constants in term of p. Here we evaluate Supplementary Theorem 20 with $\Lambda = L/2$ as this illustrative example is a generic, not fermionic, lattice Hamiltonian.

opposite spin. In terms of fermionic creation and annihilation operators the Hamiltonian for this system is

$$H_{\text{FH}} := \sum_{i=1}^{N} h_{\text{on-site}}^{(i)} + \sum_{i<j,\sigma} h_{\text{hopping}}^{(i,j,\sigma)} := u \sum_{i=1}^{N} a_{i\uparrow}^{\dagger} a_{i\uparrow} a_{i\downarrow}^{\dagger} a_{i\downarrow} + v \sum_{i<j,\sigma} \left(a_{i\sigma}^{\dagger} a_{j\sigma} + a_{j\sigma}^{\dagger} a_{i\sigma} \right), \quad (139)$$

where $\sigma \in \{\uparrow, \downarrow\}$ and the sum over hopping terms runs over all nearest neighbour fermionic lattice sites i and j. The interaction strengths are u and v and we assume that $v = 1$, and that they are bounded as $|v|, |u| \leq r$. Before we proceed we have to choose how to encode this Hamiltonian in terms of spin operators. The choice of encoding has a significant impact on the run-time of the simulation. There are many encodings in the literature [12] but we will only analyse two, the Verstraete-Cirac (VC) encoding [13], and the recent compact encoding from [14].

We choose our encoding in order to minimise the maximum Pauli weight of the encoded interaction terms. Using the VC and compact encodings this is constant at weight-4 and weight-3 respectively. In comparison the Jordan-Wigner encoding results in a maximum Pauli weight of the encoded interaction terms that scales with the lattice size as $\mathrm{O}(L)$, the Bravyi-Kitaev encoding [15] has interaction terms of weight $O(\log L)$, and the Bravyi-Kitaev superfast encoding [15] results in weight-8.

The encodings require the addition of ancillary qubits as well as two separate lattices encoding spin up and spin down fermions. For VC $4L^2$ qubits are needed to encode L^2 fermionic sites. In contrast compact requires $(L-1)^2$ ancillary qubits and $2L^2$ data qubits.

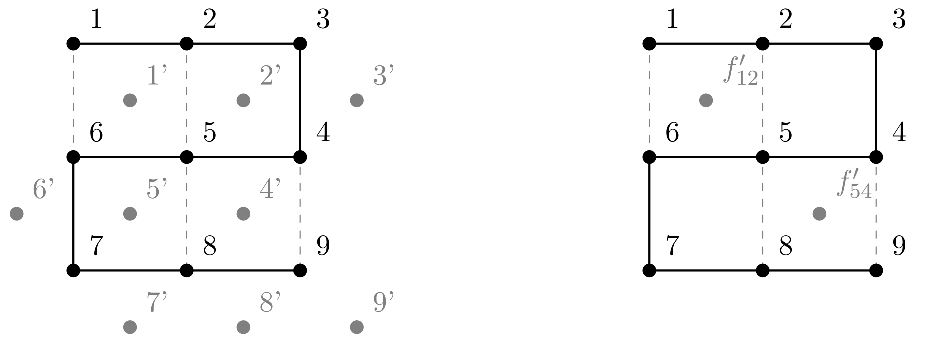

(a) VC Encoding.

(b) compact Encoding.

Supplementary Figure 7: The ordering of qubits for $L = 2$ and the layout of ancillary qubits (grey) for each encoding. This figure only depicts a single spin lattice. Additionally, the first VC ancillary qubit f'_{12} is also labelled by f'_{25}, f'_{56} and f'_{16}. Similarly for the other (VC) ancillary qubit.

The layout of these ancillary qubits are indicated in Supplementary Figure 7. Note that we must also choose an ordering of the lattice sites. This is also indicated in Supplementary Figure 7.

The two encodings map the Fermi-Hubbard Hamiltonian terms to interactions between qubits. In both encodings, on-site interaction terms become

$$h^{(i)}_{\text{on-site}} \to \frac{u}{4} (I - Z_{i\uparrow}) (I - Z_{i\downarrow}) . \tag{140}$$

Only the encoded hopping terms differ. The exact expressions for hopping interactions depend on whether two nearest neighbour fermionic sites are horizontally or vertically connected on the lattice. The horizontally connected hopping terms are encoded as

$$h^{(i,j,\sigma)}_{\text{hopping,hor}} \to \frac{1}{2} \begin{cases} X_{i,\sigma} Z_{i',\sigma} X_{j,\sigma} + Y_{i,\sigma} Z_{i',\sigma} Y_{j,\sigma} & \text{VC} \\ X_{i,\sigma} X_{j,\sigma} Y_{f'_{ij},\sigma} + Y_{i,\sigma} Y_{j,\sigma} Y_{f'_{ij},\sigma} & \text{compact} \end{cases} , \tag{141}$$

while the vertically connected hopping terms are encoded as

$$h^{(i,j,\sigma)}_{\text{hopping,vert}} \to \frac{1}{2} \begin{cases} X_{i,\sigma} Y_{i',\sigma} Y_{j,\sigma} X_{j',\sigma} - Y_{i,\sigma} Y_{i',\sigma} X_{j,\sigma} X_{j',\sigma} & \text{VC} \\ (-1)^{g(i,j)} \left(X_{i,\sigma} X_{j,\sigma} X_{f'_{ij},\sigma} + Y_{i,\sigma} Y_{j,\sigma} X_{f'_{ij},\sigma} \right) & \text{compact} \end{cases} . \tag{142}$$

In this notation i labels the data qubit for lattice site i and σ its spin lattice. Dashed indices such as i' refer to ancillary qubits. These are illustrated in grey in Supplementary Figure 7. In the VC encoding there is an ancillary qubit for every site on each spin lattice. In compact these ancillary qubits are laid out in a checker-board pattern on the faces on each spin lattice. Here f'_{ij} labels the ancillary qubit to i and j. There is also a sign determined by $g(i,j) = 0, 1$. The details of this can be found in [14].

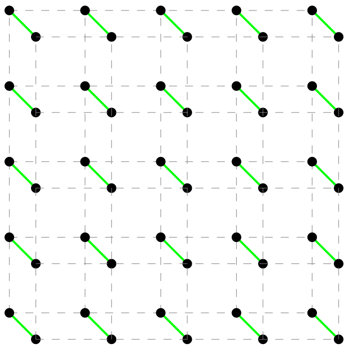

Supplementary Figure 8: The green lines connecting pairs of qubits represent a single on-site interaction term in either encoding $h^{(i)}_{\text{on-site}}$.

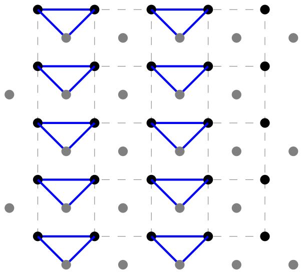

(a) The interactions in H_1.

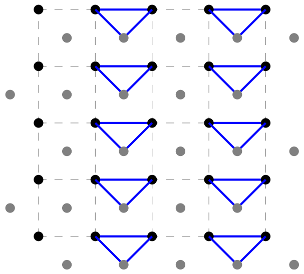

(b) The interactions in H_2

Supplementary Figure 9: The blue lines connecting three qubits represent a single horizontal hopping term in the VC encoding: $h^{(i,j,\sigma)}_{\text{hopping,hor}}$

Choice of Trotter Layers

We group the interactions into 5 Trotter layers. Every pair of interactions within a layer must be disjoint. Under the assumptions of Supplementary Definition 1 all interactions within a single layer can then be implemented in parallel. For both encodings the five layers consist of all on-site interactions H_5, two alternating layers of horizontal hopping interactions H_1 and H_2 and, two alternating layers of vertical hopping interactions H_3 and H_4. Both cases are illustrated in Supplementary Figures 9 to 12 for the case of $L = 5$.

The on-site interaction terms are the same in both cases and do not involve any ancillary qubits. They are shown in Supplementary Figure 8, where the ancillary qubits are consequently not depicted. The hopping terms all act within a single spin lattice. They are shown for the VC encoding in Supplementary Figure 9 and Supplementary Figure 10 for a single spin lattice, and for compact these are shown in Supplementary Figure 11 and Supplementary Figure 12

The alternating horizontal layers and alternating vertical layers are chosen to ensure that all pairs of interactions are disjoint and not just commuting. Note that we could have chosen to lay out the alternating horizontal and vertical layers in the VC encoding in the

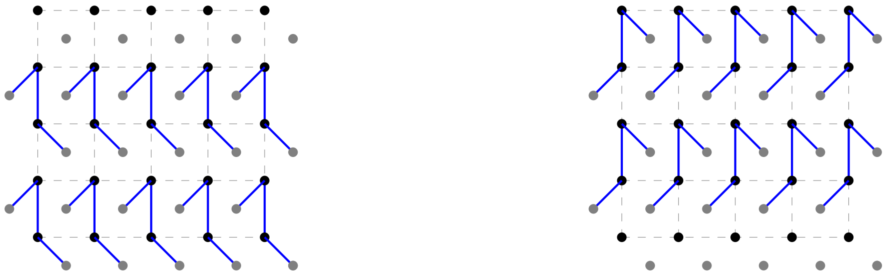

(a) The interactions in H_3. (b) The interactions in H_4.

Supplementary Figure 10: The blue lines connecting four qubits represent a single vertical hopping term in the VC encoding: $h^{(i,j,\sigma)}_{\text{hopping,vert}}$

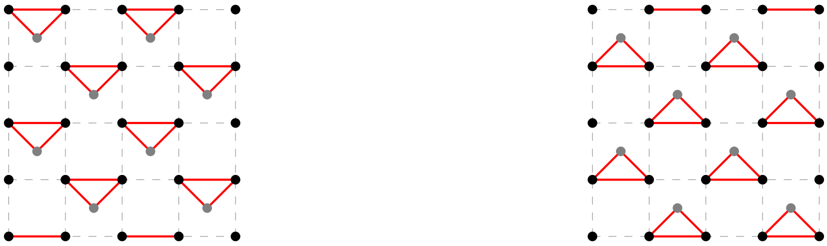

(a) The interactions in H_1. (b) The interactions in H_2.

Supplementary Figure 11: The red lines connecting three qubits represent a single horizontal hopping term in the compact encoding $h^{(i,j,\sigma)}_{\text{hopping,hor}}$.

same fashion as the compact as depicted in Supplementary Figure 11 or Supplementary Figure 12.

These are not the only choices of Trotter layers. In a later Supplementary Method called "Regrouping Interaction Terms" we show that we can implement a pth order product formula with only 3 Trotter layers. We do this for the compact encoding only as it is particularly neat. This is despite grouping the interactions in a way where not all interactions within a layer commute with one another. A combination of this and the previous results still enable us to directly implement each layer without incurring any further analytic error.

The norm of these layers appears in the Trotter bounds we derive. We bound these as $\|H_i\| \leq \Lambda$ for all i. In Supplementary Theorem 23 it is shown that Λ can be related to fermion number and this fact is used to obtain tighter bounds on the Trotter error in the numerics we perform. We confine ourselves to a sector of 5 fermions in all our numerical calculations. We do this both for pragmatic reasons, since the Hilbert space dimension is just large enough to be classically hard, and because for a 5×5 lattice a roughly quarter-filling already implies interesting crossover phenomena appear [16, 17]. We also leave Λ explicit in our analytic bounds so that we can explore different parameter regions in later work.

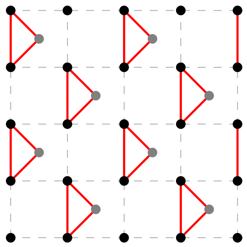

(a) The interactions in H_3.

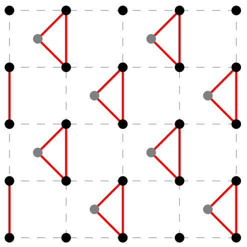

(b) The interactions in H_4.

Supplementary Figure 12: The red lines connecting three qubits represent a single vertical hopping term in the compact encoding: $h^{(i,j,\sigma)}_{\text{hopping,vert}}$.

Analytic Run-Time Bounds for Simulating Fermi Hubbard

Now we can proceed with obtaining analytic bounds on the run-time of this simulation for each encoding. Throughout this section we assume a per-time error model. For the recursive product formula in Supplementary Equation (36) with either $p=1$ or $p=2k$ for $k \geq 1$ and M non-commuting Hamiltonian layers $H=\sum_{i=1}^{M} H_i$, the cost of the simulation in terms of the single most expensive Trotter layer is

$$\mathcal{T}_{\text{cost}}\left(\mathcal{P}_p\left(\delta\right)^{T/\delta}\right) \leq \frac{MT}{\delta} \times \mathcal{T}_{\text{cost}}\left(U_{\max}\left(H, \delta B_p\right)\right) \times \begin{cases} 1 & p=1 \\ 2\times 5^{p/2-1} & p=2k \text{ for } k\geq 1, \end{cases} \tag{143}$$

where $U_{\max}(H,\tau) := \operatorname{argmax}_{U_i}\{\mathcal{T}_{\text{cost}}(U_i)\}$ for $U_i := \exp(\mathrm{i}\tau H_i)$ and B_p given in Supplementary Lemma 12. This follows from the definitions of the product formula in Supplementary Equation (36).

We proceed by obtaining bounds on the run-time of the most costly Trotter layer in each encoding. This expression depends on whether we use our methods (summarised in the main text in Supplementary Equations (11) and (12)) or the conjugation method (see Supplementary Equation (9) in the main text) to implement each Trotter step.

For the VC encoding the most costly layers will be the the vertical hopping layers H_3 and H_4. As they are both a sum of disjoint terms which we assume can be performed in parallel this is simply given by the cost of implementing a single vertical hopping interaction. Remembering that the interaction strengths satisfy $|v|$, $|u| \leq r$, we bound this as

$$\begin{aligned} \mathcal{T}_{\text{cost}}\left(U_{\max}\left(H_{\text{VC}}, \delta B_p\right)\right) &\leq \mathcal{T}_{\text{cost}}\left(\mathrm{e}^{\mathrm{i}B_p\delta\frac{r}{2}(XYYX-YYXX)}\right) && (144)\\ &= 2\,\mathcal{T}_{\text{cost}}\left(\mathrm{e}^{\mathrm{i}B_p\delta\frac{r}{2}Z^{\otimes 4}}\right) && (145)\\ &\leq 2\times\begin{cases} 7(\frac{r}{2}B_p\delta)^{1/3} & \text{sub-circuit} \\ 6\left(\frac{\pi}{4}\right) & \text{standard} \end{cases} && (146) \end{aligned}$$

	$\mathcal{T}_{\text{cost}}\left(U_{\max}\left(H, \delta B_p\right)\right)$	
Encoding	standard	sub-circuit
compact	2π	$4\sqrt{B_p r\delta}$
VC	3π	$12\sqrt[3]{B_p r\delta}$

Supplementary Figure 13: Cost of implementing the highest weight interaction term in the encoded Fermi-Hubbard Hamiltonian in a per-time error model. Decomposing a k-local evolution in terms of the standard CNOT conjugation method has overhead $2(k-1) \times \pi/4$. The overhead associated with sub-circuit synthesis follows from Supplementary Equation (18).

The second simplification follows from both terms in the interaction commuting, thus allowing them to be performed sequentially. The same is true for the compact encoding and so we have

$$\mathcal{T}_{\text{cost}}\left(U_{\max}\left(H_{\text{compact}}, \delta B_p\right)\right) \leq \mathcal{T}_{\text{cost}}\left(\mathrm{e}^{\mathrm{i}B_p\delta\frac{r}{2}(XXY+YYY)}\right) \tag{147}$$

$$= 2\,\mathcal{T}_{\text{cost}}\left(\mathrm{e}^{\mathrm{i}B_p\delta\frac{r}{2}Z^{\otimes 3}}\right) \tag{148}$$

$$\leq 2 \times \begin{cases} 2(2\frac{r}{2}B_p\delta)^{1/2} & \text{sub-circuit} \\ 4\left(\frac{\pi}{4}\right) & \text{standard} \end{cases} \tag{149}$$

The final expressions now depend only on how we decompose local Trotter steps, either in terms of CNOT gates and single qubit rotations or using circuits such as those in Supplementary Figure 3. The concrete bounds on $\mathcal{T}_{\text{cost}}\left(U_{\max}\left(H, \delta B_p\right)\right)$ are summarised and simplified in Supplementary Figure 13.

Substituting the bounds in Supplementary Figure 13 into Supplementary Equation (143) results in run-times $\propto \mathrm{O}(\delta^{-1})$ and $\propto \mathrm{O}(\delta^{\frac{2-k}{k-1}})$, assuming decomposition via Supplementary Equation (9) or Supplementary Equations (11) and (12) in the main text, respectively. As both of these expressions diverge as $\delta \to 0$ it is optimal to maximise δ with respect to an allowable analytic Trotter error ϵ_t. This is captured in the following lemma which uses the simplest bounds on Trotter error established previously.

Lemma 24 (Optimal FH δ). For a target error rate ϵ_t, the maximum Trotter step for a pth formula saturating the error bound in Supplementary Theorem 15 is

$$\delta_0 = \left(\frac{\epsilon_t}{TM^{p+1}\Lambda^{p+1}}\right)^{1/p} \times \begin{cases} 1 & p = 1 \\ beginalign10pt] \left(\frac{(p+1)!}{2}\right)^{1/p}\left(\frac{3}{10}\right)^{p/2-1/2-1/p} & p = 2k \text{ for } k \geq 1. \end{cases} \tag{150}$$

Proof. Follows from Supplementary Theorem 15 by solving for δ.

Now we can obtain the final analytic bounds on the total run-time of simulating the Fermi-Hubbard Hamiltonian for each of these four cases.

Corollary 25 (Standard-circuit Minimised Run-time). If standard synthesis techniques are used to implement local Trotter steps in terms of CNOT gates and single-qubit rotations with an optimal Trotter step size δ_0 saturating Supplementary Lemma 24, the simulation cost for the Fermi-Hubbard Hamiltonian with a pth order Trotter formula with maximum error ϵ_t is as follows

$$\mathcal{T}_{\text{cost}}(\mathcal{P}_p(\delta_0)^{T/\delta_0}) \leq \begin{cases} f_p \; M^{2+\frac{1}{p}}\Lambda^{1+\frac{1}{p}}T^{1+\frac{1}{p}}\epsilon_t^{-1/p} & \text{VC} \\ g_p \; M^{2+\frac{1}{p}}\Lambda^{1+\frac{1}{p}}T^{1+\frac{1}{p}}\epsilon_t^{-1/p} & \text{compact} \end{cases} \tag{151}$$

with

$$f_p = 3\pi \times \begin{cases} 1 & p=1 \\ 2^{\frac{p+1}{2}}3^{-\frac{p}{2}+\frac{1}{p}+\frac{1}{2}}5^{p-\frac{1}{p}-\frac{3}{2}}(p+1)!^{-\frac{1}{p}} & p=2k \text{ for } k\geq 1 \end{cases} \tag{152}$$

and

$$g_p = 2\pi \times \begin{cases} 1 & p=1 \\ 2^{\frac{p+1}{2}}3^{-\frac{p}{2}+\frac{1}{p}+\frac{1}{2}}5^{p-\frac{1}{p}-\frac{3}{2}}(p+1)!^{-\frac{1}{p}} & p=2k \text{ for } k\geq 1. \end{cases} \tag{153}$$

Proof. The proof follows by choosing δ such that the bound obtained in Supplementary Lemma 24 is saturated and substituting this and the respective expressions in Supplementary Figure 13 into Supplementary Equation (143).

Corollary 26 (Sub-Circuit Minimised Run-time). If sub-circuit synthesis techniques are used to implement local Trotter steps with an optimal Trotter step size δ_0 saturating Supplementary Lemma 24, the simulation cost for the Fermi-Hubbard Hamiltonian with a pth order Trotter formula with maximum error ϵ_t is as follows

$$\mathcal{T}_{\text{cost}}(\mathcal{P}_p(\delta_0)^{T/\delta_0}) \leq \begin{cases} f_p \; r^{1/3}M^{5/3+2/(3p)}\Lambda^{2/3+2/(3p)}T^{1+2/(3p)}\epsilon_t^{-2/(3p)} & \text{VC} \\ g_p \; r^{1/2}M^{3/2+1/(2p)}\Lambda^{1/2+1/(2p)}T^{1+1/(2p)}\epsilon_t^{-1/(2p)} & \text{compact} \end{cases} \tag{154}$$

with

$$f_p = 12 \times \begin{cases} 1 & p=1 \\ 2^{\frac{p}{2}}3^{\frac{1}{6}\left(-3p+\frac{4}{p}+4\right)}5^{\frac{1}{6}\left(5p-\frac{4}{p}-8\right)}(p+1)!^{-\frac{2}{3p}} & p=2k \text{ for } k\geq 1 \end{cases} \tag{155}$$

and

$$g_p = 4 \times \begin{cases} 1 & p=1 \\ 2^{\frac{p}{2}-\frac{1}{4}}3^{\frac{1}{4}\left(-2p+\frac{2}{p}+3\right)}5^{\frac{1}{4}\left(3p-\frac{2}{p}-5\right)}(p+1)!^{-\frac{1}{2p}} & p=2k \text{ for } k\geq 1. \end{cases} \tag{156}$$

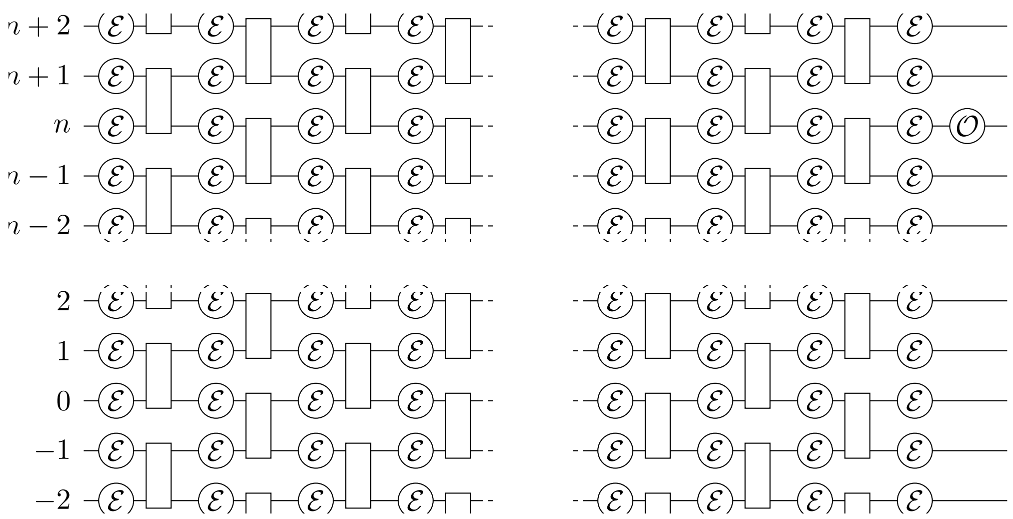

Supplementary Figure 14: Saturated circuit model with intermediate errors $\mathcal{E}$, e.g. depolarizing noise $\mathcal{E} = \mathcal{N}_q$ for some noise parameter p given in Supplementary Equation (157). At the end of the circuit, an observable $\mathcal{O}$ is measured. Drawn is a one-dimensional circuit; naturally, a similar setup can be derived for a circuit on a 2-dimensional qudit lattice, for interactions shown in Supplementary Figures 8 to 12.

Proof. The proof follows by choosing δ such that the bound obtained in Supplementary Lemma 24 is saturated and substituting this and the respective expressions in Supplementary Figure 13 into Supplementary Equation (143).

Trivial Stochastic Error Bound

So far we have only considered the unitary error introduced by approximating the real Hamiltonian evolution with a Trotterized approximation. However, in a near-term quantum device without error correction in place, we expect the simulated evolution to be noisy. We model the noise by interspersing each circuit gate in the product formula by an iid channel $\mathcal{E}$; for simplicity we will assume that $\mathcal{E}$ is a single qubit depolarising channel $\mathcal{E} = \mathcal{N}_q$, defined as

$$\mathcal{N}_q = (1-q)I + q\mathcal{T}, \quad \rho \longmapsto (1-q)\rho + \frac{q}{d}I \tag{157}$$

for a noise parameter $q \in [0, 1]$.[1] Here I denotes the identity channel and $\mathcal{T}$ takes any state to the maximally mixed state $\tau = I/d$.

[1]Strictly speaking $\mathcal{N}_q$ defines a completely positive trace preserving map for all $p \leq 1 + 1/(d^2 - 1)$. We emphasise that the error analysis which follows also works for a more general channel than the depolarising one.

A trivial error bound for a circuit as in Supplementary Figure 14 can then be found by just calculating the probability of no error occuring at all; disregarding the beneficial effects of a causal lightcone behind the observable $\mathcal{O}$, and denoting with $\mathcal{U} := U_{\mathrm{circ}}^\dagger \cdot U_{\mathrm{circ}}$ the clean circuit, and with $\mathcal{U}'$ the circuit saturated with intermediate errors, we get the expression

$$\epsilon = \left|\mathrm{Tr}\left[(\mathcal{U}(\rho) - \mathcal{U}'(\rho))\mathcal{O}\right]\right| \leq 1 - (1-q)^V, \tag{158}$$

where V is the circuit's volume (i.e. the number of $\mathcal{E}$ interspersed in $\mathcal{U}'$). It is clear to see that this error bound asymptotically approaches 1, and does so exponentially quickly. Thus, to stay below a target error rate ϵ_{tar}, a sufficient condition is that

$$1 - (1-q)^V < \epsilon_{\mathrm{tar}} \quad \Longleftrightarrow \quad V < \log\left(\frac{1-\epsilon_{\mathrm{tar}}}{1-q}\right), \tag{159}$$

or alternatively

$$\Longleftrightarrow \quad q < 1 - \sqrt[V]{1-\epsilon_{\mathrm{tar}}}. \tag{160}$$

Instead of assuming that each error channel $\mathcal{E}$ in Supplementary Figure 14 has the same error probability q, we can analyse the case where q is proportional to the pulse length of the preceding or anteceding gate; corresponding relations as given in Supplementary Equations (159) and (160) can readily be derived numerically.

Error Mapping under Fermionic Encodings

In [18], the authors analyse how noise on the physical qubits translates to errors in the fermionic code space. To first order and in the W3 encoding, all of $\{X, Y, Z\}$ errors on the face, and $\{X, Y\}$ on the vertex qubits can be detected. Z errors on the vertex qubits – as evident from the form of $h_{\mathrm{on-site}}$ from Supplementary Equation (140) – result in an undetectable error; as shown in [18, Sec. 3.2], this Z error induces fermionic phase noise.

It is therefore a natural extension to the notion of simulation to allow for some errors occur – if they correspond to physical noise in the fermionic space. And indeed, as discussed more extensively in [18, Sec. 2.4], phase noise is a natural setting for many fermionic condensed matter systems coupled to a phonon bath [19, 20, 21, 22, 23, 24, 25] and [26, Ch. 6.1&eq. 6.17].

We further assume that we can measure all stabilisers (including a global parity operator) once at the end of the entire circuit. We could imagine measuring these stabilisers after each individual gate of the form $\mathrm{e}^{\mathrm{i}\delta h_i}$ – where h_i is any term in the Hamiltonian. However, as every stabiliser commutes with every term in the Hamiltonian the outcome of the stabiliser measurement is unaffected and so we need only measure all stabilisers once at the end of the entire circuit. It is evident that measuring the stabilisers can be done by dovetailing an at most depth 4 circuit to the end of our simulation – much like measuring the stabilisers of the Toric code. It is thus a negligible overhead to the cost of simulation $\mathcal{T}_{\mathrm{cost}}$.

However, errors may occur within the decomposition of gates of the form $e^{i\delta h_i}$ into single qubit rotations and two-qubit gates of the form e^{itZZ}. The stabilisers do not generally commute with these one- and two-local gates. In spite of this we can commute a Pauli error which occurs within a decomposition past the respective gates that make up that decomposition. For example, consider $h_i = X_1 Z_2 X_3$. Then we would decompose $e^{i\delta h_i}$ first via $e^{i\delta X_1 Z_2 X_3} \approx e^{i\delta^{1/2} X_1 X_2} e^{-i\delta^{1/2} Y_2 X_3} e^{-i\delta^{1/2} X_1 X_2} e^{i\delta^{1/2} Y_2 X_3}$ and then furthermore using identities such as $e^{i\delta^{1/2} X_1 X_2} = H_1 H_2 e^{i\delta^{1/2} Z_1 Z_2} H_1 H_2$. Suppose a Pauli X error were to occur on the second qubit during one of these steps, say for example instead leading us to perform the circuit $H_1 H_2 e^{i\delta^{1/2} Z_1 Z_2} X_2 H_1 H_2 e^{-i\delta^{1/2} Y_2 X_3} e^{-i\delta^{1/2} X_1 X_2} e^{i\delta^{1/2} Y_2 X_3}$. By commuting the error X_2 to the end of the circuit we can see that this is still $\mathcal{O}(\delta^{1/2})$ close to performing $Z_2 e^{i\delta X_1 Z_2 X_3}$ as $HZ = XH$, which is an error we can detect as previously discussed. As a Pauli Z error is equally likely to occur this doesn't introduce any noise bias. Therefore in cases where δ is sufficiently small, we can use error detection to reduce the fidelity requirements needed in the device by accounting for this additional error term. This is done numerically and shown in the dashed lines of Supplementary Figures 18 and 19.

This means that while the fermionic encodings do not provide error correction, they do allow error detection to some extent; we summarise all first order error mappings in Supplementary Table 2. This means we can numerically simulate the occurrence of depolarising noise throughout the circuit, map the errors to their respective syndromes, and classify the resulting detectable errors, as well as non-detectable phase, and non-phase noise.

This means we can analyse $\mathcal{T}_{\text{cost}}$ with a demonstrably suppressed error, by allowing the non-detectable non-phase noise to saturate a target error bound. The resulting simulation is such that it corresponds to a faithful simulation of the fermionic system, but where we allow fermionic phase error occurs – where we emphasise that since detectable non-phase errors occur roughly with the same probability as non-detectable phase errors we know that, in expectation, only $O(1)$ phase error occurs throughout the simulation; in brief, it is not a very noisy simulation after all.

The resulting required depolarising noise parameters for various FH setups we summarise in Supplementary Figures 16 to 19, and the resulting post-selection probabilities in Supplementary Figure 20.

Numeric Results

We can tighten the preceding analysis in several ways. First of, instead of crudely upper bounding the cost of individual gates we can sum these pulse times exactly. To this end, we use both the explicitly defined Trotter formulae coefficients h_{ij}, and also the exact formulae for the pulse times derived previously.

Secondly, we can use tighter bounds for Trotter error, which take into account the commutation relations between pairs of interactions across Trotter layers H_i, the coefficients defined in Supplementary Lemma 12. Additionally, we obtain a bound which rewrites the Trotter error as a Taylor series, and then bound the Taylor remainder using methods which

Location	Syndrome	Effect
Vertex	X	detectable
	Y	detectable
	Z	detectable
Face (Ancilla)	X	detectable
	Y	detectable
	Z	phase noise

Supplementary Table 2: Error mapping from first order physical noise to the encoded fermionic code space, under the W3 encoding, by [18]. All but Z errors on the faces are detectable; the latter result in fermionic phase noise.

usually bound the total Trotter error; which bound is tighter depends on the order p of the formula, and the target simulation time. As explained in Supplementary Lemma 24, a tighter Trotter error allows us to choose a larger δ while achieving the same error ϵ_t, reducing the total cost of the simulation.

Finally, as explained in the Supplementary Method entitled "Trivial Stochastic Error Bound", a certain simulation circuit size will determine the amount of stochastic error present within the circuit. We assume that the depolarising noise precedes each Trotter layer; in the error-per-time model we assume that the noise parameter scales with the pulse length; in the more traditional error-per-gate model we assume that the noise is independent of the pulse length.

Furthermore, instead of taking the analytic Trotter bounds, we can also numerically calculate the optimal Trotter pulse length by calculating $\epsilon_p(T, \delta)$ from Supplementary Equation (43) explicitly, and maximizing δ till ϵ_p saturates an upper target error rate. Naturally, this is computationally costly; so we perform this calculation for FH lattices up to a size 3×3, and extrapolate these numbers by qubit count to the desired lattice lengths up to 10×10; the dependence of δ_0 on the target time and number of qubits was extracted from the asymptotic Trotter bound in Supplementary Lemma 24, i.e.

$$\delta_0 = \left(a_0 + \frac{b_0}{T^{1/p}}\right)\left(a_1 + \frac{b_1}{\Lambda^{(p+1)/p}}\right) \tag{161}$$

for four fit parameters a_0, a_1, b_0, b_1; all other dependencies are assumed constant. We show such numerically fitted data in Supplementary Figure 15; a similar analysis was made for Trotter orders $p = 1, 2, 4, 6$, and target error rates $\epsilon_t = 0.1, 0.05, 0.01$. These numerical bounds are much tigher than the analytical bounds, but can still be assumed to overestimate the actual error: the operator norm distance between Trotter evolution and true evolution likely overestimates the error that would occur if starting from a particular initialized state.

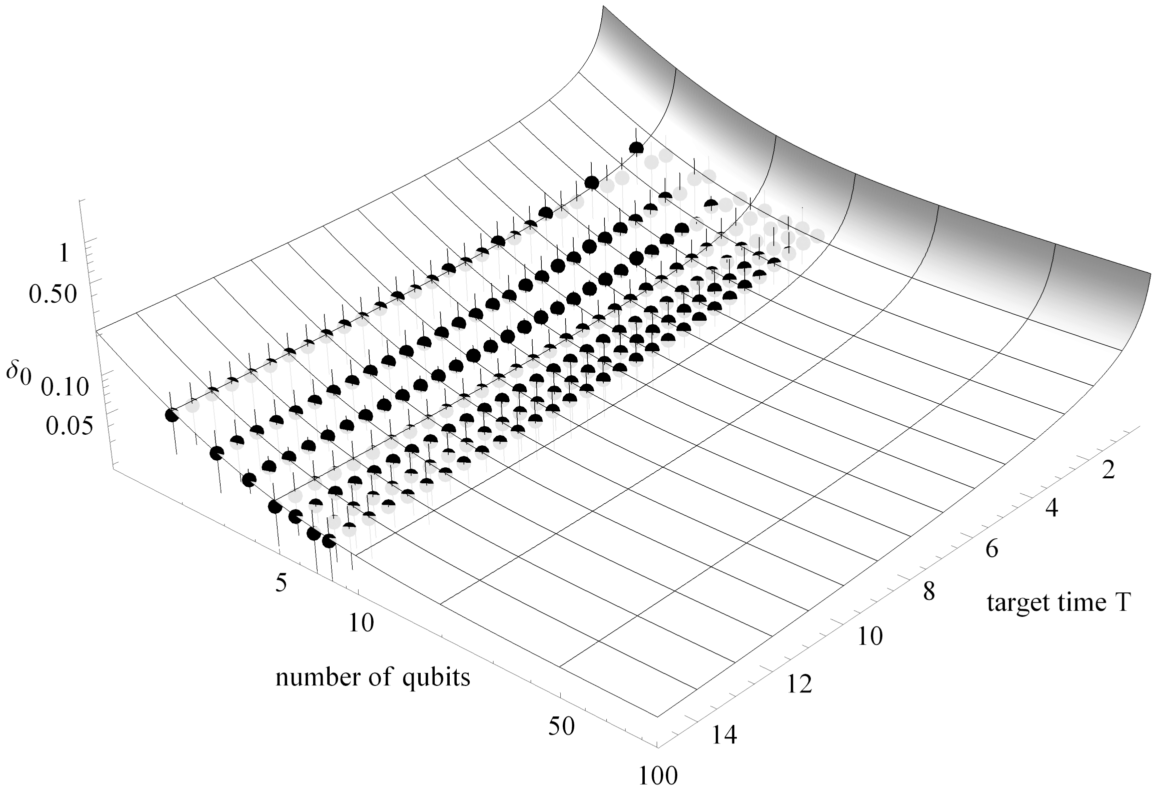

Supplementary Figure 15: Extraploated optimal Trotter error step size δ_0. The data points stem from the simulation of FH lattices up to size 3×3, for a target error rate $\epsilon_t = 0.1$, Trotter order $p = 2$, and for times $T = 0.5, 1.0, \ldots, 15.0$. The fitted surface follows the formula in Supplementary Equation (161).

We found that randomizing the Trotter layer order does not yield any advantage over keeping it fixed, and similarly we did not obtain an advantage by permuting the fixed order further.

Supplementary Table 3 compares these numerics for the case of a lattice with $L = 5$ and for a sufficiently long time in terms of units set by the lattice spacing as we assume $v = 1$. So for any L we choose $T = \lfloor \sqrt{2} L \rfloor$. We choose an analytic error of $\epsilon_t = 0.1$ as there is no point making the analytic error smaller than the experimental error present in NISQ-era gates. As the compact encoding results in the smallest run-time we investigate how $\mathcal{T}_{\text{cost}}$ varies with ϵ_t for $L = 3$, 5 and 10 below. In these numerics we choose the order p which minimise $\mathcal{T}_{\text{cost}}$ at each value of T.

	Trotter Bounds	Standard	Subcircuit
VC	analytic	95,409	17,100
	numeric	4,234	1,669
compact	analytic	77,236	1,686
	numeric	3,428	259

Supplementary Table 3: Per-time. A comparison of the run-time $\mathcal{T}_{\text{cost}}$ for lattice size $L \times L$ with $L = 5$, overall simulation time $T = 7$ and target Trotter error $\epsilon_{\text{target}} = 0.1$, with $\Lambda = 5$ fermions and coupling strengths $|u|, |v| \leq r = 1$. Obtained by minimising over product formulas up to 4th order. $\mathcal{T}_{\text{cost}} = \mathcal{T}_{\text{cost}}(\mathcal{P}_p(\delta_0)^{T/\delta_0})$ for per-time error model. In either gate decomposition case—standard and sub-circuit—we account single-qubit rotations as a free resource; the value of $\mathcal{T}_{\text{cost}}$ depends only on the two-qubit gates/interactions. Two-qubit unitaries are counted by their respective pulse lengths. Here compact and VC denote the choice of fermionic encoding.

	Trotter Bounds	Standard	Subcircuit
VC	analytic	121,478	95,447
	numeric	5391	4236
compact	analytic	98,339	72,308
	numeric	4,364	3,209

Supplementary Table 4: Per-gate. A comparison of the run-time $\mathcal{T}_{\text{cost}}$ for lattice size $L \times L$ with $L = 5$, overall simulation time $T = 7$ and target Trotter error $\epsilon_{\text{target}} = 0.1$, with $\Lambda = 5$ fermions and coupling strengths $|u|, |v| \leq r = 1$. Obtained by minimising over product formulas up to 4th order. $\mathcal{T}_{\text{cost}}$ = circuit-depth for per-gate error model. In either gate decomposition case—standard and sub-circuit—we account single-qubit rotations as a free resource; the value of $\mathcal{T}_{\text{cost}}$ depends only on the two-qubit gates/interactions. Two-qubit unitaries are counted by unit time per gate in the per gate error model. Here compact and VC denote the choice of fermionic encoding.

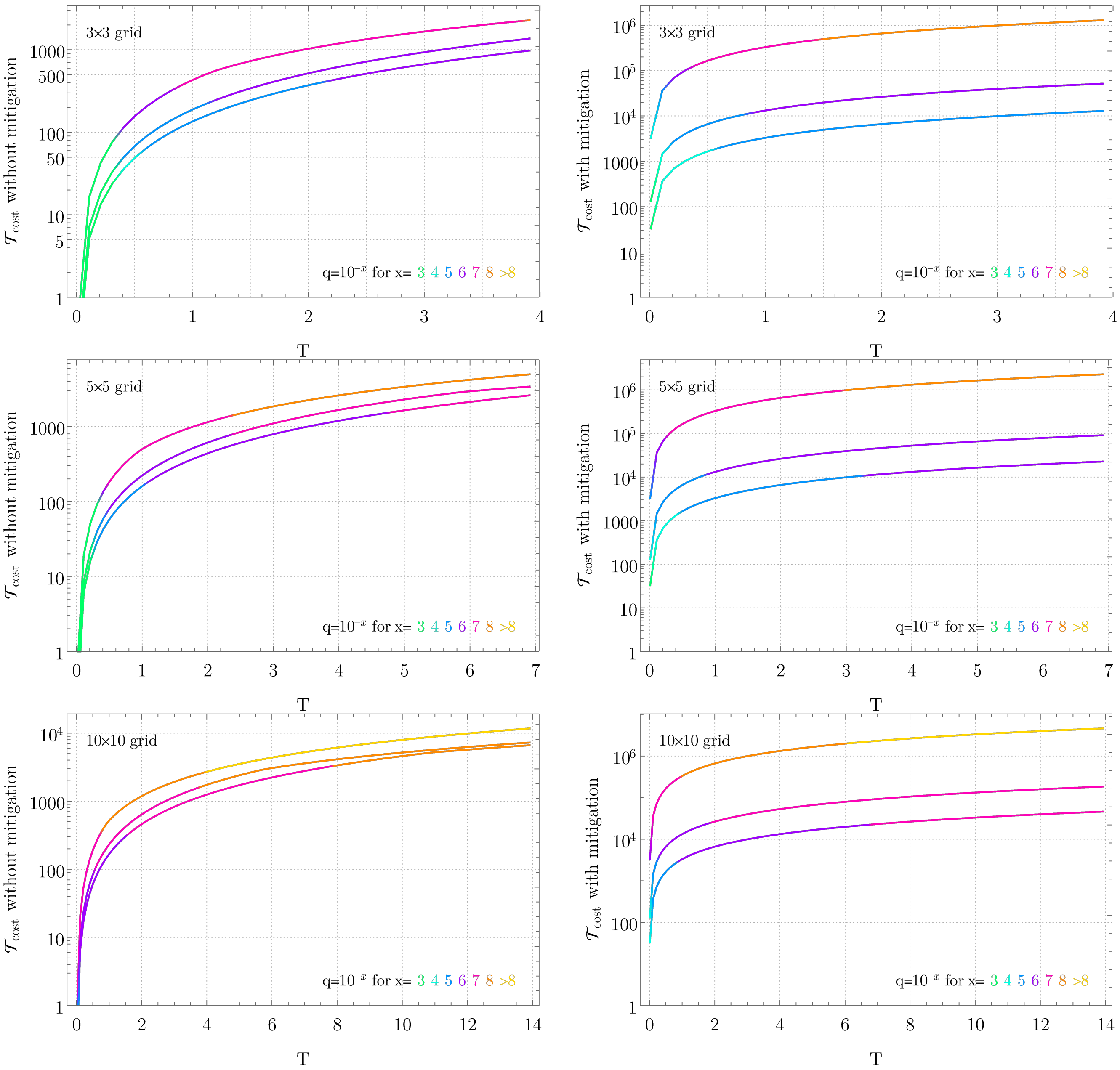

Supplementary Figure 16: $\mathcal{T}_{\text{cost}}$ vs. target time T both without (left) and with (right) error mitigation, for lattice sizes 3×3, 5×5, and 10×10, for the Fermi-Hubbard Hamiltonian H_{FH} from Supplementary Equation (139) in the compact encoding. These plots show a per-gate error model where $\mathcal{T}_{\text{cost}}$ equals circuit depth, and we synthesise local gates with a depth-3 gate decomposition by conjugation when error mitigation is not used (left) and our depth-4 decomposition (see main text) if error mitigation is used (right). In each plot, three lines (top to bottom) represent 1%, 5%, and 10% Trotter error ϵ given in Supplementary Equation (43), where we minimize over the product formula order $p \in \{1, 2, 4, 6\}$ and extrapolate a numerical simulation of FH lattices up to size 3×3 to obtain the optimal Trotter step size δ_0. The line color corresponds to the intervals wherein the *decoherence* error of the circuit is upper-bounded by the *Trotter* error of the simulation, given a specific depolarizing noise parameter q. E.g. the blue sections of the top line within each row indicate that the decoherence error remains below 1%, for $q = 10^{-5}$.

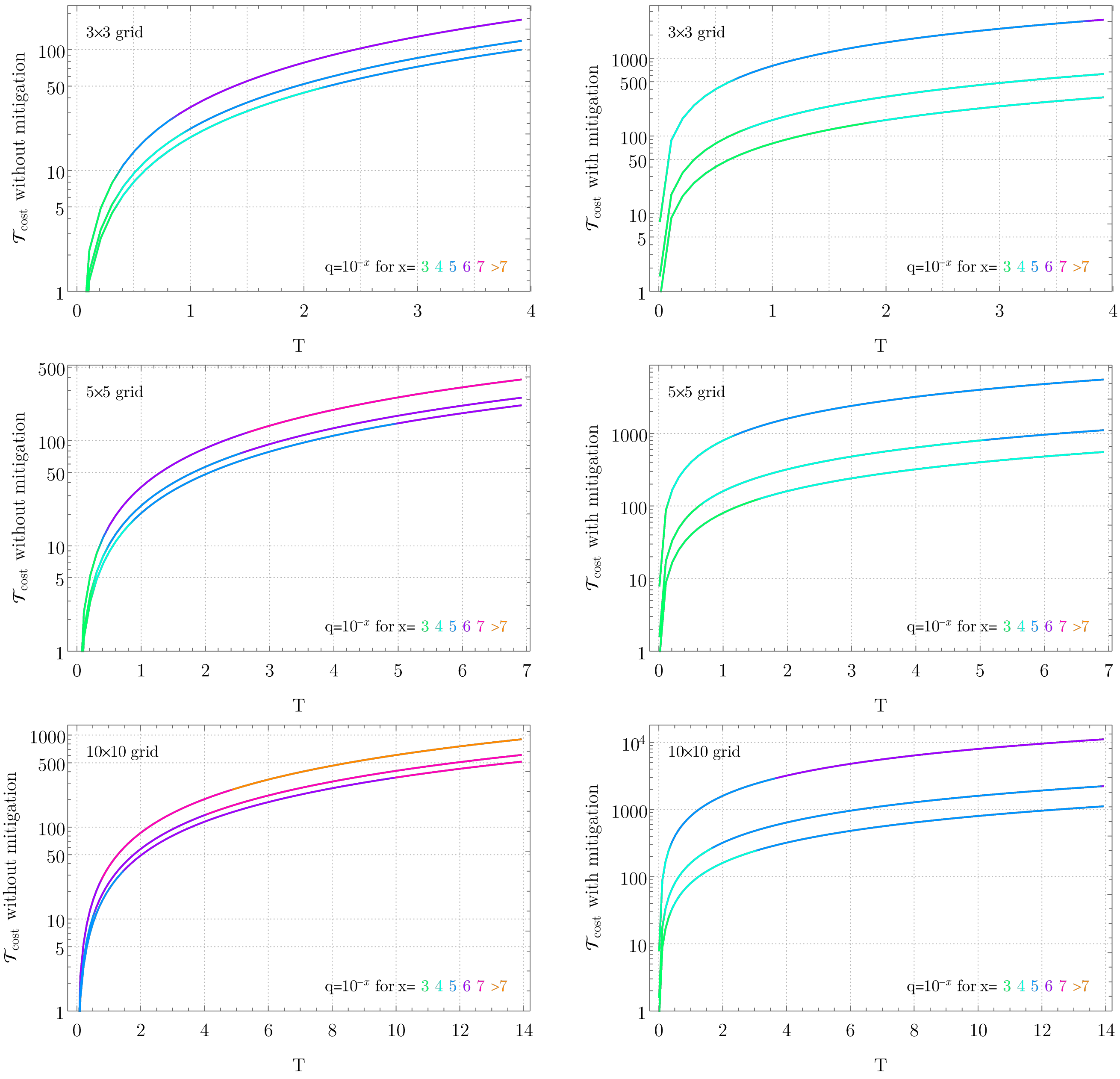

Supplementary Figure 17: $\mathcal{T}_{\text{cost}}$ vs. target time T both without (left) and with (right) error mitigation , for lattice sizes 3×3, 5×5, and 10×10, for the Fermi-Hubbard Hamiltonian H_{FH} from Supplementary Equation (139) in the compact encoding. Assuming the per-time error model where the noise probability is proportional to the gate lengths. Here $\mathcal{T}_{\text{cost}}$ denotes the circuit depth equivalent (i.e. the sum of all pulse times from Supplementary Definition 2; all 3-local gates in the compact encoding are decomposed with the depth-4 method from Supplementary Lemma 7. In each plot, three lines represent 1%, 5%, and 10% Trotter error ϵ given in Supplementary Equation (43), where we minimize over the product formula order $p \in \{1, 2, 4, 6\}$ and extrapolate a numerical simulation of FH lattices up to size 3×3 to obtain the optimal Trotter step size δ_0.

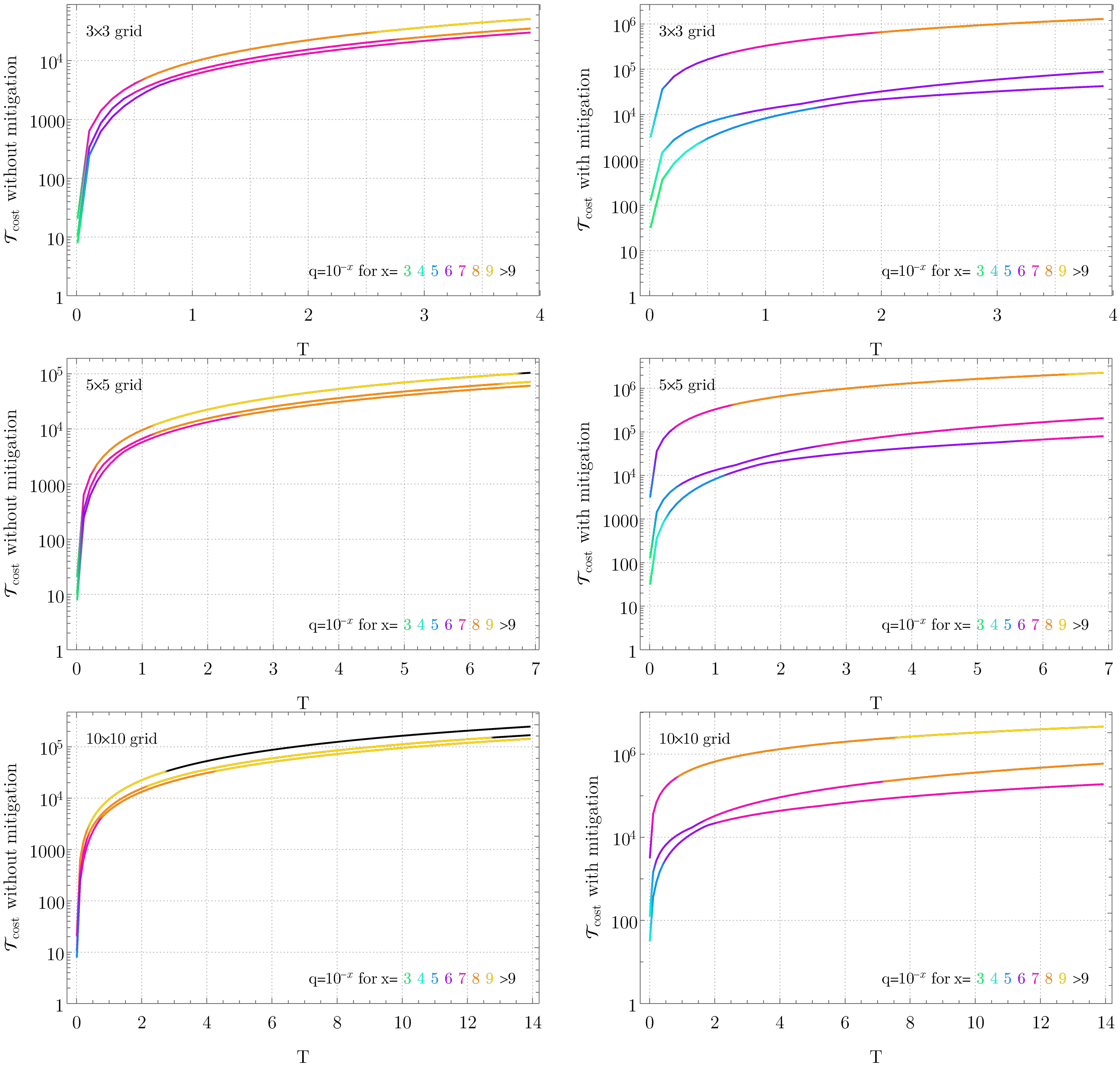

Supplementary Figure 18: Setup as in Supplementary Figure 16 with the per-gate error model, but we use the tightest analytic (instead of numeric) error expression from Supplementary Corollaries 16 and 22 and Supplementary Theorem 20. We use a depth-3 gate decomposition when error mitigation is not used (left) and a depth-4 decomposition if error mitigation is used (right).

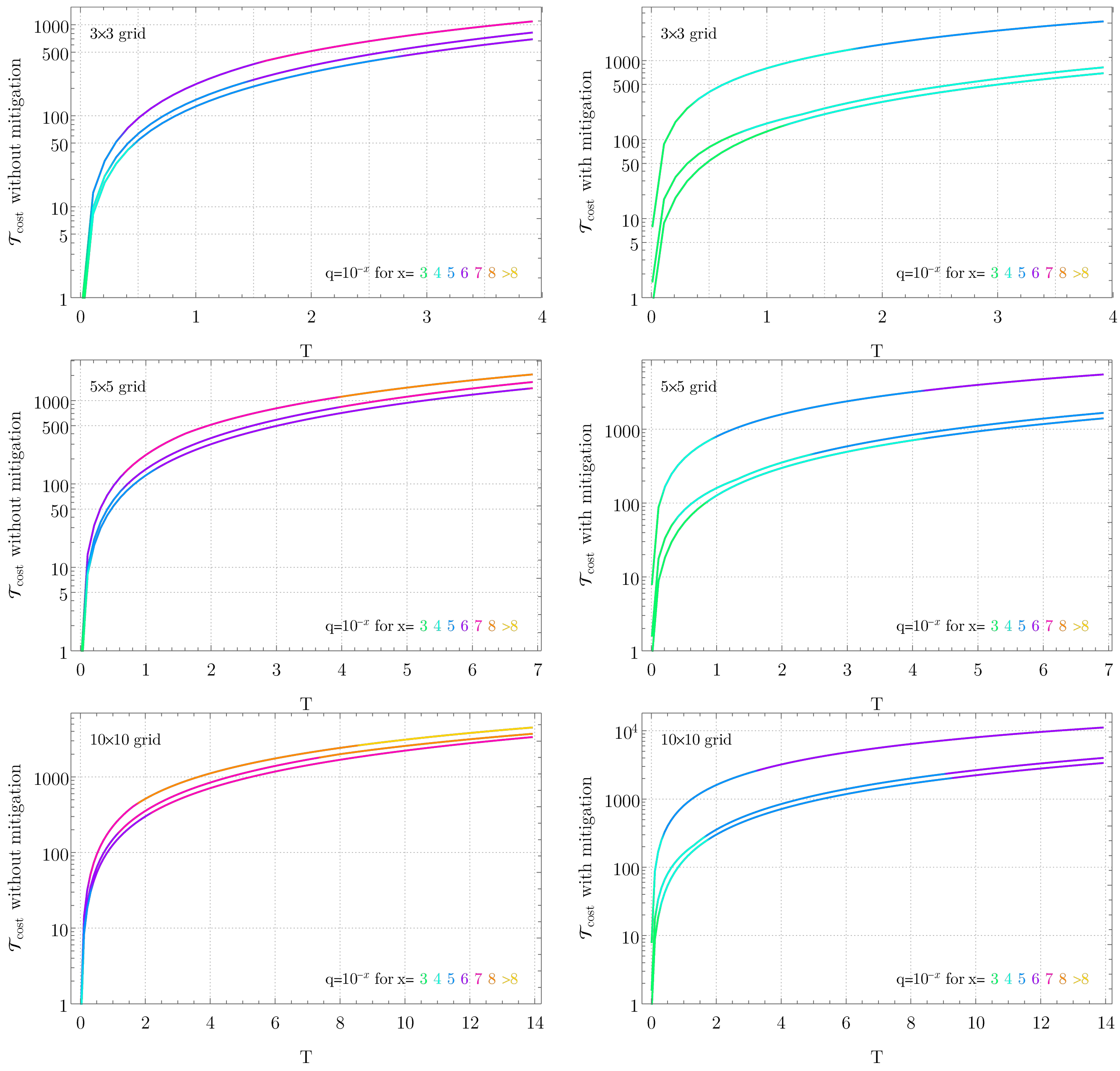

Supplementary Figure 19: Setup as in Supplementary Figure 17 with the per-time error model, but we use the tightest error expression from Supplementary Corollaries 16 and 22 and Supplementary Theorem 20. All 3-local gates in the compact encoding are decomposed with the depth-4 method from Supplementary Lemma 7.

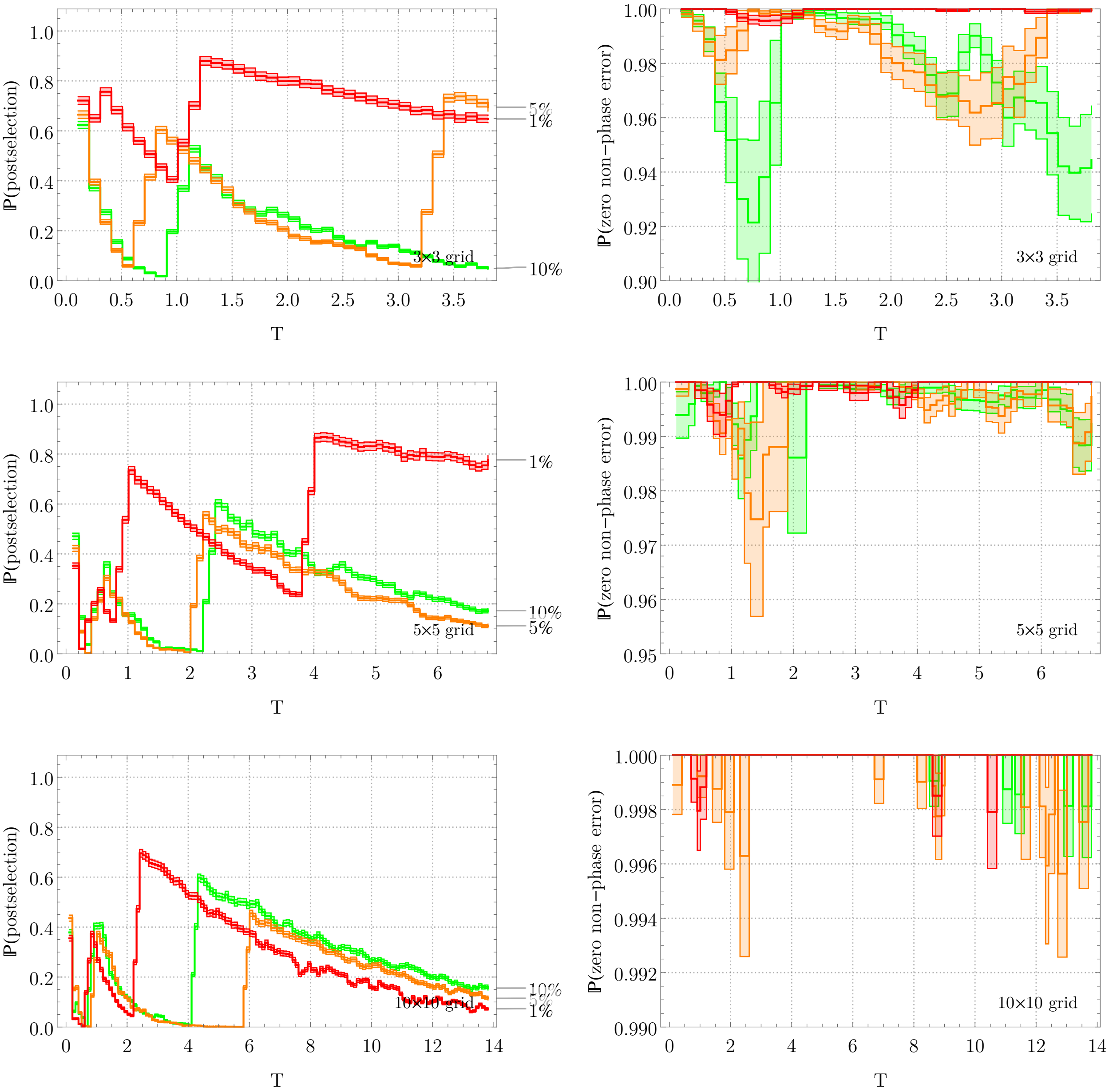

Supplementary Figure 20: Post-selection probabilities (left column) and probability of zero undetectable non-phase error after post-selection (right column) for lattice sizes 3×3, 5×5, and 10×10, for the Fermi-Hubbard Hamiltonian H_{FH} from Supplementary Equation (2) (main text) in the compact encoding, to go alongside Supplementary Figure 19. The choice between 1%, 5% and 10% Trotter error is made according to the colouring shown in Supplementary Figure 19.

Simulating Fermi-Hubbard with Three Trotter Layers

Further Circuit Decompositions

In this section we show that we can actually simulate a 2D spin Fermi-Hubbard model with $M = 3$ Trotter layers as opposed to the previous $M = 5$. First we need to introduce another circuit decomposition in the same spirit as before.

Lemma 27 (Depth 3 Decomposition). Let $U(t) = \mathrm{e}^{\mathrm{i}t(\cos(\theta)h_1+\sin(\theta)h_2)}$ be the time-evolution operator for time t under a Hamiltonian $H_\theta = \cos(\theta)h_1+\sin(\theta)h_2$. If h_1 and h_2 anti-commute and both square to identity, $U(t)$ can be decomposed as

$$U(t) = \mathrm{e}^{\mathrm{i}t_1 h_1}\mathrm{e}^{\mathrm{i}t_2 h_2}\mathrm{e}^{\mathrm{i}t_1 h_1} \tag{162}$$

where the pulse times t_1, t_2 as a function of the target time t are given by

$$t_1 = \frac{1}{2}\tan^{-1}\left(\pm\frac{\cos(t)}{\sqrt{1-\sin^2(\theta)\sin^2(t)}}, \ \pm\frac{\cos(\theta)\sin(t)}{\sqrt{1-\sin^2(\theta)\sin^2(t)}}\right) + \pi c \tag{163}$$

$$t_2 = \tan^{-1}\left(\pm\sqrt{1-\sin^2(\theta)\sin^2(t)}, \quad \pm\sin(\theta)\sin(t)\right) + 2\pi c \tag{164}$$

where $c \in \mathbb{Z}$ and signs are taken consistently throughout.

Proof. Since h_1, h_2 square to identity by assumption, we have

$$\mathrm{e}^{\mathrm{i}t_1 h_1}\mathrm{e}^{\mathrm{i}t_2 h_2}\mathrm{e}^{\mathrm{i}t_1 h_1} = I\cos(2t_1)\cos(t_2) + \mathrm{i}h_1\sin(2t_1)\cos(t_2) + \mathrm{i}h_2\sin(t_2), \tag{165}$$

and

$$\mathrm{e}^{\mathrm{i}t(\cos(\theta)h_1+\sin(\theta)h_2)} = I\cos(t) + \mathrm{i}\sin(t)\left(\cos(\theta)h_1 + \sin(\theta)h_2\right). \tag{166}$$

Equating these and solving for t_1 and t_2 gives the expressions in the Supplementary Lemma.

We then need to establish the overhead associated with implementing this decomposition. We will see that for a target time t, the pulse times in Supplementary Lemma 27 are as $t_i(t) \propto t$.

Lemma 28. Let $H = \cos(\theta)h_1+\sin(\theta)h_2$ be as in Supplementary Lemma 27. For $0 \le t \le \pi/2$ and $0 < \theta < \pi/2$, the pulse times t_1, t_2 in Supplementary Lemma 27 can be bounded by

$$|t_1| \le \frac{t}{2}, \tag{167}$$

$$|t_2| \le t\theta. \tag{168}$$

Proof. Supplementary Lemma 27 gives two valid sets of solutions for t_1, t_2. Choose the following solution:

$$t_1 = \frac{1}{2}\tan^{-1}\left(\frac{\cos(t)}{\sqrt{1-\sin^2(\theta)\sin^2(t)}}, \frac{\cos(\theta)\sin(t)}{\sqrt{1-\sin^2(\theta)\sin^2(t)}}\right) \tag{169}$$

$$t_2 = \tan^{-1}\left(\sqrt{1-\sin^2(\theta)\sin^2(t)}, \sin(\theta)\sin(t)\right). \tag{170}$$

Taylor expanding these functions about $t = 0$ and $\theta = 0$, we have

$$t_1 = \frac{t}{2} + R_1(t, \theta), \tag{171}$$

$$t_2 = t\theta + R_2(t, \theta), \tag{172}$$

Basic calculus shows that the Taylor remainders R_1, R_2 are always negative for the stated range of t, giving the stated bounds.

Regrouping Interaction Terms

Now we apply this and the previous decompositions to simulate H_{FH} as encoded using the compact encoding, using only three Trotter layers: $\{H_0, H_1, H_2\}$. The first of these layers consists of all the on-site interactions:

$$H_0 := \frac{u}{4}\sum_{i=1}^{N}(I - Z_{i\uparrow})(I - Z_{i\downarrow}), \tag{173}$$

and the other two layers are a *mix* of horizontal and vertical hopping terms. Each has the same form, but consists of different sets of interactions as shown in Supplementary Figure 21.

$$H_{1/2} := +\frac{v}{2}\sum_{i<j}\sum_{\sigma\in\{\uparrow,\downarrow\}}\left(X_{i,\sigma}X_{j,\sigma}Y_{f(i,j),\sigma} + Y_{i,\sigma}Y_{j,\sigma}Y_{f(i,j),\sigma}\right) \tag{174}$$

$$+\frac{v}{2}\sum_{i<j}\sum_{\sigma\in\{\uparrow,\downarrow\}} g(i,j)\left(X_{i,\sigma}X_{j,\sigma}X_{f(i,j),\sigma} + Y_{i,\sigma}Y_{j,\sigma}X_{f(i,j),\sigma}\right). \tag{175}$$

Now we show that $\mathrm{e}^{\mathrm{i}\delta H_1}$ can be implemented directly; the case of $\mathrm{e}^{\mathrm{i}\delta H_2}$ follows similarly. As H_1 consists of interactions on disjoint sets of qubits, each forming a square on four qubits in Supplementary Figure 21, we only need to show that we can implement evolution under the interactions making up one of the squares. We will denote these by h_i. We will label an example h_1 as shown in Supplementary Figure 22 and demonstrate that this can be done. Using this labelling, the square of interactions is given by

$$h_1 = \frac{1}{2}(X_1X_2 + Y_1Y_2)Y_a + \frac{1}{2}(X_3X_4 + Y_3Y_4)Y_a \tag{176}$$

$$+\frac{1}{2}(X_1X_4 + Y_1Y_4)X_a - \frac{1}{2}(X_2X_3 + Y_2Y_3)X_a. \tag{177}$$

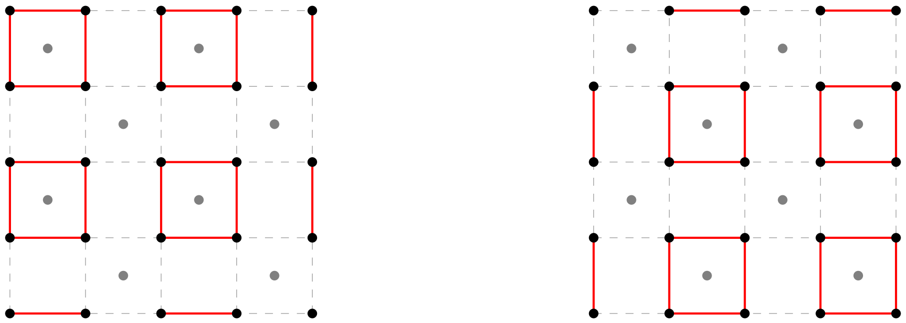

Supplementary Figure 21: The interactions in H_1 (left) and those in H_2 (right). Gray nodes represent ancillary qubits, all non-gray qubits encode fermionic sites of a particular spin.

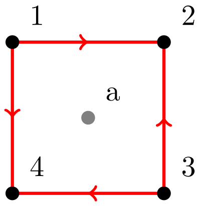

Supplementary Figure 22: The Hamiltonian h_1: a *sum* of each Pauli interaction represented by a red line connecting a pair of qubits. Upward pointing arrows indicate $g(i,j) = -1$ and downward, left and right pointing arrows indicate $g(i,j) = 1$ (See [14]). H_1 is a sum of disjoint Hamiltonians of this form, shown in Supplementary Figure 21

Now we will regroup these interactions in such away that we can use the methods of Supplementary Lemma 27 to decompose $\mathrm{e}^{\mathrm{i}\delta h_1}$. To do this we group the terms as $h_1 = a_1 + a_2 + b_1 + b_2$, where

$$a_1 = \frac{1}{\sqrt{2}}\left(\frac{X_1X_2Y_a - X_aX_2X_3}{\sqrt{2}}\right) \tag{178}$$
$$a_2 = \frac{1}{\sqrt{2}}\left(\frac{Y_1Y_4X_a + Y_aY_4Y_3}{\sqrt{2}}\right) \tag{179}$$
$$b_1 = \frac{1}{\sqrt{2}}\left(\frac{Y_1Y_2Y_a - X_aY_2Y_3}{\sqrt{2}}\right) \tag{180}$$
$$b_2 = \frac{1}{\sqrt{2}}\left(\frac{X_1X_4X_a + Y_aX_4X_3}{\sqrt{2}}\right). \tag{181}$$

We have reordered terms in order to easily verify the following commutation and anti-commutation relations: (i). Every a_i and b_i squares to something proportional to the identity and consists of two anti-commuting Pauli terms. (ii). The only pairs of a_i and b_i which don't commute, instead anti-commute:

$$\{a_1, b_2\} = 0 \tag{182}$$
$$\{a_2, b_1\} = 0. \tag{183}$$

It is easy to verify that all other pairs of a_i and b_i commute. This allows us to implement the target evolution as follows

$$\mathrm{e}^{\mathrm{i}\delta h_1} = \mathrm{e}^{\mathrm{i}\delta(a_1+b_2)}\mathrm{e}^{\mathrm{i}\delta(a_2+b_1)}. \tag{184}$$

Now we only need to show that we can implement $\mathrm{e}^{\mathrm{i}\delta(a_1+b_2)}$; the implementation of $\mathrm{e}^{\mathrm{i}\delta(a_2+b_1)}$ follows similarly. Consider the Hamiltonian

$$a_1 + b_2 = \frac{1}{\sqrt{2}}\left(\frac{X_1X_2Y_a - X_aX_2X_3}{\sqrt{2}}\right) + \frac{1}{\sqrt{2}}\left(\frac{X_1X_4X_a + Y_aX_4X_3}{\sqrt{2}}\right). \tag{185}$$

This meets the criteria to decompose $\mathrm{e}^{\mathrm{i}\delta(a_1+b_2)}$ with two applications of Supplementary Lemma 27. The first application gives the decomposition

$$\mathrm{e}^{\mathrm{i}\delta(a_1+b_2)} = \mathrm{e}^{\mathrm{i}t_1\frac{X_1X_2Y_a - X_aX_2X_3}{\sqrt{2}}}\mathrm{e}^{\mathrm{i}t_2\frac{X_1X_4X_a + Y_aX_4X_3}{\sqrt{2}}}\mathrm{e}^{\mathrm{i}t_1\frac{X_1X_2Y_a - X_aX_2X_3}{\sqrt{2}}} \tag{186}$$

where the pulse times are a function of the target time $t_i = t_i(\delta)$ as defined in Supplementary Lemma 27. Then we apply Supplementary Lemma 27 again, to each of the individual gates in Supplementary Equation (186). The first gate in Supplementary Equation (186) decomposes as

$$\mathrm{e}^{\mathrm{i}t_1\frac{X_1X_2Y_a - X_aX_2X_3}{\sqrt{2}}} = \mathrm{e}^{t_1(t_1)X_1X_2Y_a}\mathrm{e}^{t_2(t_1)X_aX_2X_3}\mathrm{e}^{t_1(t_1)X_1X_2Y_a}, \tag{187}$$

and the others decompose similarly. Now we need only apply Supplementary Lemma 7 to decompose these three qubit unitaries into evolution under two-local Pauli interactions. As Supplementary Lemma 28 shows that up until now the pulse times have remained $\propto \delta$, it is only this final step which introduces a root overhead. Hence the run-time remains asymptotically as Supplementary Corollary 26.

Feasible simulation time as a function of hardware error rate

In this Appendix we analyse the simulation performance from the perspective of hardware error rate, rather than circuit depth. Specifically, we analyse the following question: given a maximum allowable simulation error ϵ_{tar} and a given hardware noise rate q, for how long can we simulate a given Fermi Hubbard Hamiltonian? That is, what is the maximum simulation time T_{tar} such that the total simulation error remains below the target threshold, $\epsilon \leq \epsilon_{tar}$? In Supplementary Table 5 we consider this question for an $L = 5$ Fermi Hubbard model with target error $\epsilon_{tar} = 10\%$.

When error mitigation is unused the only contribution to the total error ϵ is from Trotter error ϵ_t and error due to stochastic noise ϵ_s. That is we have $\epsilon = \sqrt{\epsilon_t^2 + \epsilon_s^2} \leq \epsilon_{tar}$. When mitigation is used there is an additional contribution ϵ_c from commuting errors past short-time gates. That is $\epsilon = \sqrt{\epsilon_t^2 + \epsilon_s^2 + \epsilon_c^2} \leq \epsilon_{tar}$. All Trotter bounds used to produce Supplementary Table 5 are numeric. We are considering a fermion occupation number of $\Lambda = 5$.

In Supplementary Table 5 we consider both per-time and per-gate error models, as well as standard circuit decompositions and subcircuit decompositions. Recall that the former only allows a standard gate set, whereas the latter allows access to a continuous family of two-qubit gates.

In the per-gate error model, the error mitigation techniques described in the Supplementary Method entitled "Trivial Stochastic Error Bound" do not apply, and we choose the decomposition which yields the lowest depth circuit. In both the standard and subcircuit decompositions, this implies using the conjugation technique Supplementary Equation (9) rather than the decomposition techniques of before. For example, when decomposing 3-local terms, using a standard gate set this conjugation decomposition must be applied twice to leave us with only single qubit rotations and gates that are equivalent to CNOT gates (up to single-qubit rotations). Whereas if we are in a per-gate error model but allowing a subcircuit gate-set, then we only need to decompose via conjugation once.

Where error mitigation can be applied in the per-gate model, we decompose all gates via our subcircuit decompositions as otherwise error mitigation is not possible. Similarly, in the per-time error model, we always use these decompositions, as they are always more efficient than conjugation decompositions in that model.

We see that in the per-time error model using the compact encoding, using our subcircuit synthesis with the error mitigation techniques they enable yields the best performance across the hardware error rates considered. For $q = 10^{-6}$ admitting subcircuit gates and allowing for error mitigation takes us from a maximum simulation time of $T_{tar} = 3.17$ to $T_{tar} = 896$. For the VC encoding, error mitigation does not yield an improvement. However subcircuit gates and our subcircuit synthesis techniques do, taking us from $T_{tar} = 2.25$ to $T_{tar} = 3.81$ for $q = 10^{-6}$.

For both encodings smaller improvements are seen for $q = 10^{-5}$ and $q = 10^{-4}$. In the per-gate error model for the compact encoding, we find that our subcircuit decomposition

techniques and error mitigation strategy yields an improvement, but over error rates of $q = 10^{-6}$ and $q = 10^{-5}$. For the VC encoding we see that error mitigation does not help. However for $q = 10^{-6}$ subcircuit gates still yield an improvement, taking us from $T_{tar} = 1.63$ to $T_{tar} = 2.2$.

In cases where error mitigation is used we include the classical post-selection overhead. We have deliberately capped this at a maximum of $\approx 10^4$, to disallow excessive post-selection overhead. In some cases this cap is reached, indicating that our error mitigation techniques could yield further improvement still, but at a potentially unreasonably high post-selection overhead.

error model	encoding	decomp	mitigation	post selection overhead	T_{tar}	δ	$\lceil T_{tar}/\delta \rceil$	$\mathcal{T}_{\text{cost}}$
					3.17	0.133	24	1273
per time	compact	standard	False	n/a	0.74	0.258	3	155
					0.23	0.456	1	27
					28.2	0.054	520	1377
		subcircuit	False	n/a	3.77	0.123	31	123
					0.55	0.299	2	12
				9.1e3	896.	0.005	179e3	144e3
		subcircuit	True	9.8e3	89.8	0.005	18.0e3	14e3
				9.1e3	8.84	0.005	1770	1416
					2.25	0.155	15	956
	VC	standard	False	n/a	0.48	0.318	2	100
					0.23	0.456	1	33
					3.81	0.123	31	803
		subcircuit	False	n/a	1.00	0.226	5	141
					0.23	0.456	1	21
				1.8e4	0.27	1.65e-6	165e3	95e3
		subcircuit	True	8.5e3	0.04	1.65e-6	25.0e3	14e3
				9.8e3	0.00416	1.65e-6	2530	1451
					2.87	0.139	21	1407
per gate	compact	standard	False	n/a	0.51	0.308	2	113
					0.23	0.456	1	34
					3.53	0.127	28	1393
		subcircuit	False	n/a	0.83	0.245	4	170
					0.23	0.456	1	25
				1.2e4	11.1	0.005	2211	146e3
		subcircuit	True	2.1e4	1.08	0.005	216	14e3
				1.6e4	0.11	0.005	22	1465
					1.63	0.180	9	761
	VC	standard	False	n/a	0.48	0.318	2	126
					0.23	0.456	1	42
					2.20	0.157	14	929
		subcircuit	False	n/a	0.50	0.312	2	106
					0.23	0.456	1	33
				2.6e4	0.000932	1.65e-6	567	96e3
		subcircuit	True	5.1e3	0.000135	1.65e-6	82	14e3
				8.0e3	0.0000140	1.65e-6	9	1440

Supplementary Table 5: For $L = 5$ with $\epsilon_{tar} = 10\%$ and $q = 10^{-6},\ 10^{-5}$ and 10^{-4} from top to bottom.

Supplementary References

References

[1] Morten Kjaergaard, Mollie E. Schwartz, Jochen Braumüller, Philip Krantz, Joel I-Jan Wang, Simon Gustavsson, and William D. Oliver. Superconducting Qubits: Current State of Play. *Annual Review of Condensed Matter Physics*, 11(1):369–395, may 2019.

[2] David Poulin, Angie Qarry, Rolando Somma, and Frank Verstraete. Quantum simulation of time-dependent hamiltonians and the convenient illusion of hilbert space. *Phys. Rev. Lett.*, 106:170501, Apr 2011.

[3] Wolfgang Dür, Michael J Bremner, and Hans J Briegel. Quantum simulation of interacting high-dimensional systems: the influence of noise. Technical report, 2007.

[4] Andrew M. Childs, Dmitri Maslov, Yunseong Nam, Neil J. Ross, and Yuan Su. Toward the first quantum simulation with quantum speedup. *Proceedings of the National Academy of Sciences*, 115(38):9456–9461, 2018.

[5] Andrew M Childs and Yuan Su. Nearly optimal lattice simulation by product formulas. Technical report, January 2019.

[6] Andrew M. Childs, Yuan Su, Minh C. Tran, Nathan Wiebe, and Shuchen Zhu. A Theory of Trotter Error. *arXiv:1912.08854*, dec 2019.

[7] S. Lloyd. Universal Quantum Simulators. *Science*, 273(5278):1073–1078, aug 1996.

[8] Man-Hong Yung, James D. Whitfield, Sergio Boixo, David G. Tempel, and Alán Aspuru-Guzik. Introduction to Quantum Algorithms for Physics and Chemistry. In *Introduction to Quantum Algorithms for Physics and Chemistry*, pages 67–106. mar 2014.

[9] Masuo Suzuki. General theory of higher-order decomposition of exponential operators and symplectic integrators. *Physics Letters A*, 1992.

[10] Masuo Suzuki. General theory of fractal path integrals with applications to many-body theories and statistical physics. *Journal of Mathematical Physics*, 32(2):400–407, feb 1991.

[11] Johannes Bausch. On the Efficient Calculation of a Linear Combination of Chi-Square Random Variables with an Application in Counting String Vacua. *Journal of Physics A: Mathematical and Theoretical*, 46(50):505202, aug 2012.

[12] P. Jordan and E. Wigner. Über das Paulische Äquivalenzverbot. *Zeitschrift für Physik*, 47(9-10):631–651, sep 1928.

[13] F. Verstraete and J. I. Cirac. Mapping local Hamiltonians of fermions to local Hamiltonians of spins. *Journal of Statistical Mechanics: Theory and Experiment*, aug 2005.

[14] Charles Derby and Joel Klassen. A compact fermion to qubit mapping. 2020.

[15] Sergey B. Bravyi and Alexei Yu Kitaev. Fermionic quantum computation. *Annals of Physics*, 298(1):210–226, may 2002.

[16] M. Keller, W. Metzner, and U. Schollwöck. Dynamical mean-field theory for pairing and spin gap in the attractive Hubbard model. *Physical Review Letters*, 86(20):4612–4615, may 2001.

[17] Tatsuya Kaneko and Yukinori Ohta. BCS-BEC crossover in the two-dimensional attractive hubbard model: Variational cluster approach. *Journal of the Physical Society of Japan*, 83(2), feb 2014.

[18] Johannes Bausch, Toby Cubitt, Charles Derby, and Joel Klassen. Mitigating errors in local fermionic encodings. 2020.

[19] H. T. Ng. Decoherence of interacting Majorana modes. *Scientific Reports*, 5(1):1–14, jul 2015.

[20] A. Kauch, P. Pudleiner, K. Astleithner, P. Thunström, T. Ribic, and K. Held. Generic Optical Excitations of Correlated Systems: π-tons. *Physical review letters*, 124(4):047401, jan 2020.

[21] Xinyu Zhao, Wufu Shi, J. Q. You, and Ting Yu. Non-Markovian dynamics of quantum open systems embedded in a hybrid environment. *Annals of Physics*, 381:121–136, jun 2017.

[22] Alexey A. Melnikov and Leonid E. Fedichkin. Quantum walks of interacting fermions on a cycle graph. *Scientific Reports*, 6(1):1–13, sep 2016.

[23] L. A. Openov and A. V. Tsukanov. Selective electron transfer between quantum dots induced by a resonance pulse. *Semiconductors*, 39(2):235–242, 2005.

[24] L. Fedichkin and A. Fedorov. Error rate of a charge qubit coupled to an acoustic phonon reservoir. *Phys. Rev. A*, 69:032311, Mar 2004.

[25] Marlan O. Scully and Jonathan P. Dowling. Quantum-noise limits to matter-wave interferometry. *Physical Review A*, 48(4):3186–3190, oct 1993.

[26] Wellington Luiz Ribeiro. *Evolution of a 1D Bipartite Fermionic Chain Under Influence of a Phenomenological Dephasing.* PhD thesis, Universidade Federal do ABC (UFABC), 2014.
